# Psilocybin or Nicotine Patch for Smoking Cessation: A Pilot Randomized Clinical Trial

**DOI:** 10.1001/jamanetworkopen.2026.0972

**Published:** 2026-03-10

**Authors:** Matthew W. Johnson, Gideon P. Naudé, Peter S. Hendricks, Albert Garcia-Romeu

**Affiliations:** 1 Department of Psychiatry and Behavioral Sciences, Johns Hopkins University School of Medicine, Baltimore, Maryland; 2 Department of Psychiatry and Behavioral Neurobiology, University of Alabama at Birmingham, Birmingham

## Abstract

**Importance:**

Annual tobacco-related death estimates are 480 000 in the US and 8 million worldwide, surpassing mortality for other abused substances. Most smoking cessation interventions result in modest long-term success.

**Objective:**

To compare prolonged smoking abstinence rates in smokers receiving psilocybin plus cognitive behavioral therapy (CBT) with those receiving the nicotine patch plus CBT.

**Design, Setting, and Participants:**

In this pilot randomized clinical trial, participants and investigators were unblinded to treatment condition, including an optional crossover after completion of the primary end point. Data were collected from psychiatrically healthy adult smokers from January 20, 2015, to May 8, 2023, at the Johns Hopkins Bayview Medical Center, an academic medical center and teaching hospital in Baltimore, Maryland.

**Intervention:**

The trial randomized cigarette smokers to receive either 1 high dose (30 mg/70 kg) of psilocybin or initiate 8 to 10 weeks of US Food and Drug Administration–approved nicotine patch treatment on the target quit date. Both groups received a 13-week manualized CBT program for smoking cessation.

**Main Outcomes and Measures:**

Biochemically verified prolonged smoking abstinence (primary) and 7-day point prevalence abstinence (secondary) at 6 months after the target quit date were compared between groups using intention-to-treat analysis.

**Results:**

A total of 82 psychiatrically healthy adult smokers (mean [SD] age, 47.6 [12.0] years; 49 [59.8%] male) participated in the study, with 68 (82.9%) completing the 6-month follow-up. At 6-month follow-up, 17 participants receiving psilocybin (40.5%) exhibited biochemically verified prolonged abstinence compared with 4 participants using the nicotine patch (10.0%) (odds ratio, 6.12; 95% CI, 1.99-23.26; *P* = .003), and 22 participants receiving psilocybin (52.4%) exhibited biochemically verified 7-day point prevalence abstinence compared with 10 participants using the nicotine patch (25.0%) (odds ratio, 3.30; 95% CI, 1.32-8.70; *P* = .01). No serious adverse events were attributed to psilocybin or nicotine patch.

**Conclusions and Relevance:**

In this pilot randomized clinical trial, 1 dose of psilocybin with manualized CBT significantly increased long-term abstinence compared with nicotine patch treatment with CBT. Psilocybin abstinence rates were higher than typical treatments, suggesting promise for tobacco smoking cessation.

**Trial Registration:**

ClinicalTrials.gov Identifier: NCT01943994

## Key Points

**Question:**

What tobacco smoking cessation rates does psilocybin, an experimental treatment, yield compared with nicotine patch, an established treatment, with individuals in both groups receiving standardized cognitive behavioral therapy?

**Findings:**

In this pilot randomized clinical trial, 42 participants randomized to receive psilocybin had more than 6 times greater odds of prolonged smoking abstinence 6 months after treatment than 40 participants who received the nicotine patch.

**Meaning:**

These results suggest that psilocybin holds potential in the treatment of tobacco use disorder and, along with other psychedelics, should be investigated further for tobacco and other substance use disorders.

## Teaser

This pilot randomized clinical trial compares prolonged smoking abstinence rates in smokers receiving psilocybin with those receiving the nicotine patch with both groups receiving manualized smoking cessation cognitive behavioral therapy.

## Introduction

Smoking is among the leading causes of premature death, with approximately 8 million deaths annually,^1^ including approximately 480 000 in the US.^2^ These rates are several-fold higher than for alcohol or other substances, including opioids.^3^ More than two-thirds of US smokers want to quit,^4^ indicating an unmet need warranting novel approaches. Available treatments (ie, varenicline, bupropion, nicotine replacement therapy [NRT], and counseling)^5,6^ are more effective than placebo but typically fail within 6 months.^6,7^

Serotonin 2A receptor agonist (classic) psychedelics show preliminary promise for treating psychiatric conditions, including addictions.^8^ Older research showed promising evidence for lysergic acid diethylamide in treating alcohol and opioid use disorders.^9,10^ On the basis of these data suggesting antiaddiction efficacy of serotonin 2A receptor agonists across multiple addictive drugs, an open-label pilot study was conducted by some of the current authors (M.W.J. and A.G.R.) examining psilocybin for tobacco smoking cessation.^11^ Fifteen participants underwent manualized cognitive behavioral therapy (CBT) for smoking cessation and received up to 3 psilocybin administrations (20-30 mg/70 kg). Biologically verified 7-day point prevalence abstinence rates were 80%, 67%, and 60% at 6, 12, and 30 months after the target quit date, respectively.^12^ No serious adverse events (AEs) attributable to psilocybin occurred. Epidemiologic data suggest classic psychedelic use may be associated with lower tobacco use,^13^ and naturalistic research describes classic psychedelic use leading to smoking cessation.^14^

The current randomized clinical trial compared a single psilocybin dose (30 mg/70 kg) with a standard US Food and Drug Administration (FDA)–approved course of nicotine patch, using manualized smoking cessation CBT in both groups similar to that used in the pilot.^11,15^ Nicotine patch was selected because it has widely characterized safety and efficacy.^16^

## Methods

**Figure 1.**
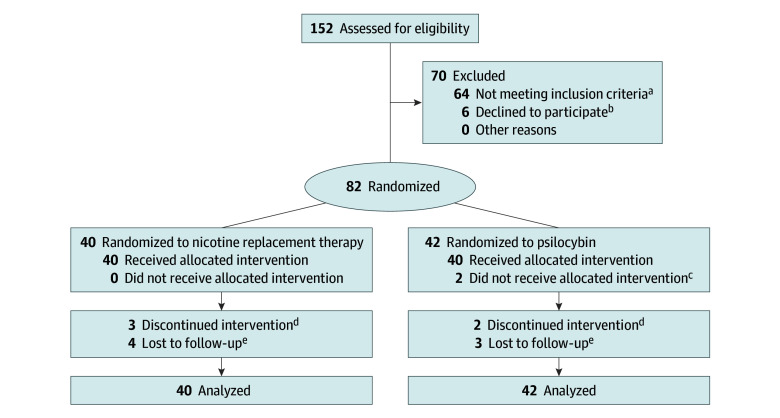
Study Flow Diagram ^a^Reasons for disqualification at screening: cardiovascular risks (high blood pressure or abnormal electrocardiographic findings; n = 13); not responsive (n = 13); smoked fewer than 5 cigarettes per day (n = 8); psychiatric comorbidities (eg, alcohol use disorder; n = 6); scheduling difficulties (n = 5); body mass index outside normal range (n = 3); positive drug screen (n = 3); concerns about time commitment (n = 3); recent or extensive hallucinogen use (n = 3); recent history of substance use disorder (n = 3); family history of psychiatric illness (eg, schizophrenia; n = 2); pregnancy (n = 1); and negative past experience using psilocybin (n = 1). ^b^In addition to those disqualified at screening, 6 individuals were screened and enrolled but dropped out of the study before randomization in week 3 due to life issues or difficulty attending study visits. ^c^Did not receive psilocybin due to COVID-19–related research shutdown (n = 1) and death in family (n = 1). ^d^Participants nonresponsive to contact attempting to schedule upcoming treatment visits. These individuals received the study drug (ie, psilocybin or nicotine patch) but did not complete the behavioral treatment portion. ^e^Participants who completed the treatment portion but were nonresponsive to contact attempting to schedule follow-up visits.

In this pilot randomized clinical trial with a comparative efficacy design, 152 individuals were screened and 70 were disqualified for not meeting criteria (n = 64) or declining participation (n = 6), resulting in a final analytic sample of 82 participants (Figure 1). To characterize sample demographics, we collected self-reported data on participant race and ethnicity via a survey querying US Census racial categories: White, Black or African American, American Indian or Alaska Native, Asian, and Native Hawaiian or Other Pacific Islander, with the addition of “some other race” and “more than one race.” Ethnicity data were collected via the survey question, “Do you identify as Hispanic or Latino?” Sample size was informed by financial rather than statistical considerations because of limited data on effect size from prior research. The study initially targeted a total sample of 30, increasing later to targeted samples of 50, 80, and 100 as more funding became available and to support functional magnetic resonance imaging (fMRI) data validity (not reported here). The sample size of 82 reflects what was viable to complete with the available funding support. See eAppendix 1 in Supplement 1 for supplementary information. This study was approved by an institutional review board at Johns Hopkins University School of Medicine and followed the 1964 Declaration of Helsinki.^17^ Participants provided written informed consent. There was no formal patient or public involvement in the design, conduct, and reporting of the trial. This report follows the Consolidated Standards of Reporting Trials (CONSORT) reporting guideline for randomized trials.^18^ The trial protocol can be found in Supplement 2.

### Participants

Participants were recruited with advertisements seeking “cigarette smokers interested in a novel research approach to quitting smoking” posted at local businesses, on the internet (Facebook and a study website), and in newspaper and radio advertisements and by word-of-mouth referral. Interested individuals underwent screening by online questionnaire and telephone to assess major inclusion and exclusion criteria before in-person screening. Participants received no monetary compensation for procedures reported in this article.

### Eligibility Criteria

Eligible volunteers were daily smokers aged 21 to 80 years with more than 1 previous unsuccessful quit attempt and continued desire to quit smoking. Medical questionnaires, physical examination, electrocardiography, and blood and urine tests were administered to exclude pregnancy, cardiovascular conditions (eg, uncontrolled hypertension), and other medical concerns (see eAppendix 2 in Supplement 1 for full inclusion and exclusion criteria). A Structured Clinical Interview for *Diagnostic and Statistical Manual of Mental Disorders* (Fourth Edition) was conducted to exclude those with current or past psychotic disorders or bipolar I or II disorder, current or past alcohol or drug dependence or severe major depression in the last 5 years, and/or other personal or family history of serious mental health concerns.

### Procedures

Participants received a 13-week CBT intervention based on techniques reported as effective in previous studies^19,20^ and used in the pilot, including use of a daily smoking diary, considering reasons for quitting vs continuing smoking, health and financial drawbacks of smoking, and developing strategies to manage craving and withdrawal. Participants were randomized 1:1 by the principal investigator (M.W.J.) to psilocybin or nicotine patch in their third treatment visit in week 3 using urn stratification, balancing the dichotomous variables of intelligence (high defined as IQ >114), tobacco dependence severity (high defined as Fagerström Test for Cigarette Dependence [FTCD] score >4), age (high defined as aged >51 years), and sex.^21,22,23^ Urn stratification uses a probabilistic algorithm to promote unbiased balanced assignment.^23^

**Figure 2.**
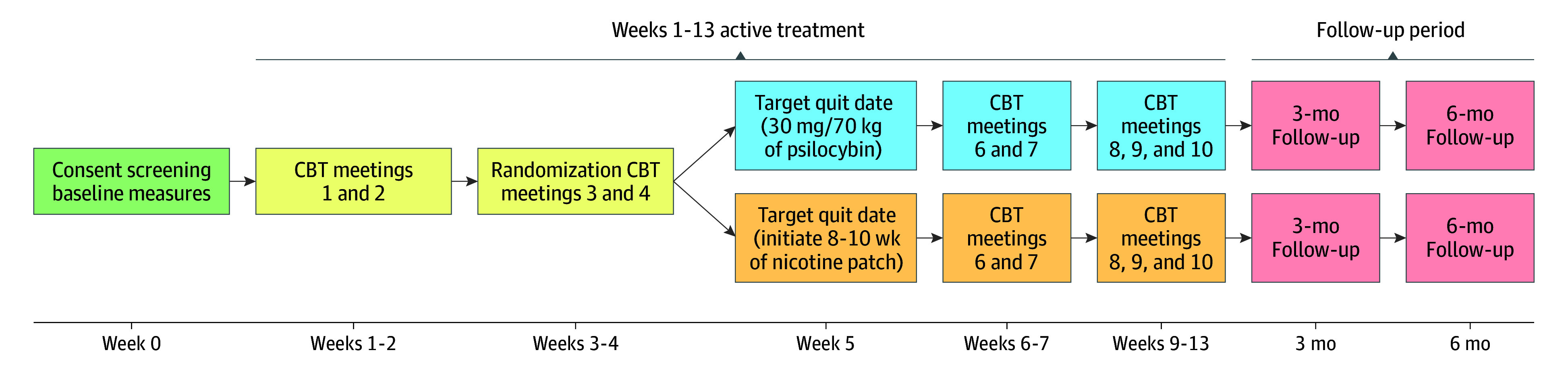
Timeline Flowchart of the Study Treatment Intervention and Follow-Ups The 3- and 6-month follow-ups occurred approximately 3 and 6 months after the target quit date in week 5, respectively. CBT indicates cognitive behavioral therapy.

In the first 4 treatment visits, participants underwent CBT with a target quit date set in week 5. In week 5, one group received 30 mg/70 kg of psilocybin and the other received a course of nicotine patches (Figure 2). Two facilitators who were aware of treatment assignments (a doctoral/master’s-level psychologist [A.G.R.] and a research coordinator with a bachelor’s degree or higher level in mental health) delivered the intervention. All participants were contacted daily via telephone (<5 minutes) or text message in the week after the target quit date to encourage smoking abstinence. Participants had weekly visits with facilitators across weeks 1 to 7 and at 2-week intervals across weeks 9 to 13, with follow-up visits 3, 6, and 12 months after the target quit date. Study visits were in-person until March 2020, when most study visits became virtual due to COVID-19, except psilocybin administration, 3- to 12-month follow-up visits, and nicotine patch pick-up visits. To reduce differential dropout, we gave participants randomized to the nicotine patch group the option of a single crossover psilocybin administration after completing the 6-month follow-up (primary end point). A subsample of participants underwent fMRI before and after treatment. Results from the 12-month follow-up, crossover, and fMRI and potential mechanisms will be published separately.

### Treatment Groups

The participants randomized to the psilocybin group received psychoeducation regarding psilocybin effects during treatment visits 3 and 4 and were administered 30 mg/70 kg of psilocybin at visit 5 (target quit date). The next day, participants attended a debriefing visit with facilitators and met weekly through week 7 and every 2 weeks from weeks 9 to 13 to discuss the psilocybin experience and support smoking abstinence. During preparatory visits, participants were encouraged to view the psilocybin experience as an opportunity for reflection about the role of smoking in their lives and to enhance quitting motivation. During psilocybin dosing, participants typically lay on a couch wearing eyeshades, were asked to focus attention inward, and listened to a music program through headphones.

The participants randomized to the nicotine patch group received standardized psychoeducation, including use instructions and potential AE information. In week 5, these participants began an 8- to 10-week regimen per FDA labeling (>10 cigarettes per day [CPD] smokers received 21 mg/d on weeks 1-6, 14 mg/d on weeks 7-8, and 7 mg/d on weeks 9-10; ≤10 CPD smokers received 14 mg/d on weeks 1-6 and 7 mg/d on weeks 7-8). Like the psilocybin group, participants had counseling visits weekly through week 7 and biweekly in weeks 9 to 13 to discuss treatment and support smoking abstinence.

### Biological Markers of Smoking Abstinence

Exhaled breath carbon monoxide detected smoking in the past day, and urinary cotinine detected smoking in the past week.^23^ Both were assessed at intake and at the 3-, 6-, and 12-month follow-up visits. Carbon monoxide was also assessed at all visits (in weeks 1-7, 9, 11, and 13). Carbon monoxide was assessed using a breath carbon monoxide monitor (Micro Smokerlyzer, Bedfont Scientific Ltd) at in-person visits or a Bluetooth carbon monoxide monitor (mobile iCo Smokerlyzer, Bedfont Scientific Ltd)^24^ at virtual visits. Cotinine samples were collected and analyzed by Friends Medical Laboratory (Baltimore, Maryland) or via AimStep rapid qualitative cotinine tests (during COVID-19).

### Self-Report Smoking-Related Measures

A timeline follow-back (TLFB), retrospective, self-report calendar indicating the number of cigarettes smoked each day was completed at each study visit.^25^ The TLFB has shown good validity and reliability as a measure of daily smoking.^26^ The FTCD,^22^ a 6-item questionnaire characterizing dependence severity, the Contemplation Ladder assessing readiness to quit smoking on a 0- to 10-point scale,^27^ and the Questionnaire on Smoking Urges^28^ assessing smoking craving were completed at intake.

### Adverse Events

From the target quit date through the final follow-up, AEs were assessed by asking participants, “How have you been feeling since your last visit?” Staff recorded any new or worsening physical and mental health events self-reported or observed during study visits. AEs were deemed related or unrelated to the study intervention based on temporal proximity, participant attribution, and investigators’ judgment.

### Outcome Measures

The a priori primary study outcome was prolonged smoking abstinence at 6-month follow-up, with 7-day point prevalence abstinence and mean post–target quit date CPD as a priori secondary outcomes. Prolonged abstinence was defined as no smoking following an initial 14-day grace period after the target quit date.^16^ Seven-day point prevalence abstinence was defined as no self-reported smoking (not even a puff) in the 7 days preceding a visit. For both outcomes, abstinence was determined using carbon monoxide, cotinine, and TLFB. A carbon monoxide level of 5 ppm or less and cotinine levels of less than 200 ng/mL indicated nonsmoking.^29^ In cases where these conflicted due to reported use of other noncombustible nicotine products, participants were coded as smoking abstinent if TLFB and CO both indicated no smoking. Additional analyses counted any noncombustible nicotine product use or positive cotinine as nonabstinent, assessing potential impact on treatment outcomes (eAppendix 1 in Supplement 1). Those randomized to a group were counted as part of that group’s abstinence rates regardless of receiving the intervention.

### Statistical Analysis

Data were collected from January 20, 2015, to May 8, 2023. Intention-to-treat analysis included all randomized participants; those who discontinued treatment or were lost to follow-up were counted as nonabstinent. Statistical tests were performed using R statistical software, version 4.4.1 (R Foundation for Statistical Computing)^30^ and GraphPad Prism, version 10.4.0 (GraphPad Software). Intake demographic and clinical characteristics were evaluated for normality using Shapiro-Wilk tests (where appropriate) and compared between groups using Welch *t* tests, Mann-Whitney *U* tests, or Fisher exact tests. Prolonged and 7-day point prevalence abstinence rates between groups were analyzed using binary logistic regression. These models contained no covariates due to highly comparable group baseline characteristics. Odds ratios (ORs) are reported with 95% CIs. Daily cigarette use between the target quit date and 6-month follow-up was evaluated as part of an exploratory analysis using generalized linear mixed-effects models suitable for count data with overdispersion. The models were fit to daily smoking data, with a random intercept for participant to account for correlation among repeated within-participant observations. Treatment group was included as the primary fixed effect, and baseline CPD was included as a covariate to adjust for pretreatment smoking levels. Analyses were restricted to participants who provided TLFB data extending at least through the 6-month follow-up period (n = 68) to ensure coverage of the full analysis window. No data were imputed. The outcome, based on the TLFB, was modeled as a rate with a log-transformed offset for the number of follow-up days. A second model included a zero-inflation term to account for an excess of zero-use days likely attributable to prolonged abstinence rather than random variability. Detailed model specifications are provided in eAppendix 1 in Supplement 1. All tests were 2-tailed (α = .05).

## Results

Eighty-two participants (mean [SD] age, 47.6 [12.0] years; 49 [59.8%] male and 33 [40.2%] female; 3 [3.7%] Black or African American, 4 [4.9%] East or Southeast Asian, 73 [89.0%] White, and 2 [2.4%] multiracial) were randomized, with 42 randomized to psilocybin and 40 to nicotine patch^31^ (Table 1). Participants smoked a mean of 15.7 CPD and reported a median of 6 previous quit attempts. Demographic and baseline characteristics were comparable between groups. Sixty-eight participants (82.9%) completed the 6-month follow-up (35 in the psilocybin group and 33 in the nicotine patch group). One primary facilitator delivered treatment for 40 psilocybin participants (95.2%) and 35 nicotine patch participants (87.5%). Another primary facilitator delivered the intervention to the remainder of participants (eTables 1 and 3 in Supplement 1). Across the study, psilocybin participants spent a median (range) of 29.6 (9.3-39.8) hours in treatment visits (excluding screening and follow-ups) vs 16.8 (5.5-28.6) hours for patch participants. This difference is primarily accounted for by psilocybin administration that typically lasted 8 to 9 hours and next-day psilocybin debriefing visits. Included in these durations is time spent in occasional meetings with nonfacilitator staff (ie, study investigators) for both groups. These discussions generally involved additional assessment of safety, study feedback, and encouragement for cessation. Median (range) total durations of these meetings were 0.46 (0-1.6) and 0.23 (0-1.6) hours, respectively. Two individuals randomized to psilocybin discontinued treatment before receiving psilocybin. Two participants randomized to psilocybin discontinued treatment 2 weeks after receiving psilocybin and therefore did not complete CBT, leaving a total of 38 individuals (90.5%) randomized to psilocybin who received the drug and completed CBT treatment. Three of these individuals were subsequently lost to follow-up. Two individuals randomized to nicotine patch were adjusted to a lower dose due to AEs but completed study treatment. Three others in the patch group discontinued treatment early, only 1 of whom received the full course of patches. Thus, of the 40 participants randomized to the nicotine patch condition, 38 (95.0%) completed a full course of nicotine patches, and 37 (92.5%) completed the CBT. Four of these individuals were subsequently lost to follow-up.

**Table 1.** Demographic and Clinical Characteristics of Study Participants at Study Intake

Characteristic	No. (%) of participants^a^		Total (N = 82)
	Psilocybin group (n = 42)	Nicotine patch group (n = 40)	
Age, mean (SD) [range], y	48.5 (11.9)	46.8 (12.3)	47.6 (12.0) [25-73]
Sex			
Male	23 (54.8)	26 (65.0)	49 (59.8)
Female	19 (45.2)	14 (35.0)	33 (40.2)
Race^b^			
Black or African American	3 (7.1)	0	3 (3.7)
East or Southeast Asian	1 (2.4)	3 (7.5)	4 (4.9)
White	38 (90.5)	35 (87.5)	73 (89.0)
Multiracial	0	2 (5.0)	2 (2.4)
Hispanic ethnicity	2 (4.8)	0	2 (2.4)
Educational level of bachelor’s degree or higher	24 (57.1)	26 (65.0)	52 (63.4)
Shipley Institute of Living scale score, mean (SD)^c^	122.03 (8.96)	122.12 (7.80)	122.07 (8.36)
CPD, mean (SD) [range]	15.92 (6.93)	15.51 (7.34)	15.72 (7.09) [5-40]
FTCD, mean (SD) [range]	4.74 (1.84)	4.40 (1.81)	4.57 (1.82) [0-9]
Contemplation Ladder score, mean (SD) [range]	9.48 (0.94)	9.35 (0.89)	9.41 (0.92) [7-10]
QSU score, mean (SD) [range]			
Total	118.62 (15.04)	116.90 (17.06)	117.78 (15.98) [68-164]
Factor 1: reward	45.45 (6.93)	44.48 (6.98)	44.98 (6.93) [29-60]
Factor 2: relief	49.79 (10.96)	49.63 (10.57)	49.71 (10.71) [29-81]
Carbon monoxide, mean (SD) [range]	18.45 (8.02)	16.10 (7.78)	17.30 (7.94) [6-44]
Urinary cotinine, mean (SD) [range], µg/L	1417.43 (757.01)	1370.05 (723.09)	1393.74 (735.90) [259-3660]
Previous quit attempts, median (IQR)	6 (4-10)	5 (3-7)	6 (4-10)
Lifetime classic psychedelic use^d^	27 (64.3)	26 (65.0)	53 (64.6)
No. of classic psychedelic uses, median (IQR) [range]	6 (1-15)	4 (2-8)	5 (2-11) [1-20]

Abbreviations: CPD, cigarettes smoked per day; FTCD, Fagerström Test for Cigarette Dependence; QSU, Questionnaire on Smoking Urges.

SI conversion factors: To convert cotinine to nanomoles per liter, multiply by 5.675.

^a^ Unless otherwise indicated.

^b^ Participant race and ethnicity were self-reported via a survey querying US Census racial categories: Asian, American Indian or Alaska Native, Black or African American, Native Hawaiian or Other Pacific Islander, and White with the addition of “some other race” and “more than one race.” Ethnicity data were collected via a survey asking, “Do you identify as Hispanic or Latino?”

^c^ Shipley Institute of Living scale scores were transformed to age-adjusted Wechsler Adult Intelligence Scale–Revised equivalent intelligence scores.^31^

^d^ Previous classic psychedelic uses reported only for individuals with at least 1 previous experience with a classic psychedelic (eg, psilocybin, lysergic acid diethylamide, dimethyltryptamine, or mescaline).

### Adverse Events

No serious study-related AEs occurred. A total of 70 participants (85.4%) reported an AE during the study (Table 2). More AEs were recorded on the target quit date for the psilocybin group (35 [87.5%]) vs the nicotine patch group (11 [27.5%]). These AEs largely consisted of anticipated, modest elevations in blood pressure and heart rate that were monitored in the psilocybin group but not assessed in the nicotine patch group (eTable 2 in Supplement 2). During psilocybin administration, the mean (SD) peak systolic blood pressure was 160.2 (2.3) mm Hg, the mean (SD) peak diastolic blood pressure was 100.9 (1.9) mm Hg, and the mean (SD) peak heart rate was 87.6/min (2.3/min) (eFigure 1 in Supplement 1). In one psilocybin administration, 0.4 mg of sublingual nitroglycerin was physician administered to manage hypertension, with no further sequelae. Otherwise, the most common AEs during the target quit date were headaches, which were expected (20 [50.0%] in the psilocybin group vs 3 [7.5%] in the nicotine patch group) (eTable 2 in Supplement 1).

**Table 2.** Summary of Adverse Events During the 6-Month Trial Period and on the Target Quit Date

Adverse event	6-mo Trial period^a^				Target quit date			
	Psilocybin, No. (%) (n = 40)^b^	NRT, No. (%) (n = 40)	RR (95% CI)	*P* value^c^	Psilocybin, No. (%) (n = 40)^b^	NRT, No. (%) (n = 40)	RR (95% CI)	*P* value^c^
Any adverse event^d^	37 (92.5)	33 (82.5)	1.12 (0.94-1.37)	.31	35 (87.5)	11 (27.5)	3.18 (2.0-5.49)	<.001
Serious adverse event	0	1 (2.5)	0.0 (0.0-3.8)	>.99	0	0	NC	>.99
Related adverse event^e^	35 (87.5)	23 (57.5)	1.52 (1.16-2.11)	.005	34 (85.0)	10 (25.0)	3.4 (2.06-6.06)	<.001
Adverse event reported in ≥2 participants during the trial								
Depression	12 (30.0)	9 (22.5)	1.33 (0.65-2.79)	.61	1 (2.5)	0	NC	>.99
Headache	22 (55.0)	4 (10.0)	5.5 (2.26-14.37)	<.001	20 (50.0)	3 (7.5)	6.67 (2.38-20.04)	<.001
Elevated blood pressure^f^	24 (60.0)	0	NC	<.001	24 (60.0)	0	NC	<.001
Respiratory infection	7 (17.5)	9 (22.5)	0.78 (0.33-1.83)	.78	0	0	NC	>.99
Anxiety	9 (22.5)	12 (30.0)	0.75 (0.36-1.55)	.61	1 (2.5)	0	NC	>.99
Insomnia	3 (7.5)	8 (20.0)	0.38 (0.11-1.19)	.19	0	3 (7.5)	0.0 (0.0-1.23)	.24
Gastrointestinal illness or diarrhea	8 (20.0)	4 (10.0)	2.0 (0.70-5.89)	.35	1 (2.5)	0	NC	>.99
Rash	0	7 (17.5)	0.0 (0.0-0.52)	.01	0	1 (2.5)	0.0 (0.0-3.77)	>.99
Irritability	4 (10.0)	2 (5.0)	2.0 (0.45-9.01)	.68	1 (2.5)	0	NC	>.99
Back pain	5 (12.5)	3 (7.5)	1.67 (0.47-6.02)	.71	2 (5.0)	0	NC	.49
Nausea	3 (7.5)	3 (7.5)	1.0 (0.24-4.13)	>.99	3 (7.5)	0	NC	.24
Visual disturbance	6 (15.0)	0	NC	.03	2 (5.0)	0	NC	.49
Vivid dreams	0	6 (15.0)	0.0 (0.0-0.60)	.03	0	4 (10.0)	0.0 (0.0-.91)	.12
Fatigue	3 (7.5)	2 (5.0)	1.5 (0.31-7.24)	>.99	3 (7.5)	0	NC	.24
Joint pain	1 (2.5)	4 (10.0)	0.25 (0.04-1.58)	.36	1 (2.5)	1 (2.5)	1.0 (0.17-9.39)	>.99
Migraine	2 (5.0)	1 (2.5)	2.0 (0.27-14.96)	>.99	1 (2.5)	0	NC	>.99
Elevated heart rate^f^	2 (5.0)	2 (5.0)	1.0 (0.18-5.47)	>.99	2 (5.0)	0	NC	.49
Concentration impairment	2 (5.0)	1 (2.5)	2.0 (0.27-14.96)	>.99	0	0	NC	>.99
Fever	0	2 (5.0)	0.0 (0.0-1.86)	.49	0	0	NC	>.99
Ear infection	0	2 (5.0)	0.0 (0.0-1.86)	.49	0	0	NC	>.99

Abbreviations: NC, not calculable; NRT, nicotine replacement therapy; RR, relative risk.

^a^ Six-month trial period data are inclusive of target quit date data.

^b^ Only individuals who received the study intervention are included.

^c^ Fisher exact test.

^d^ For a full listing of adverse events, see eTable 2 in Supplement 2.

^e^ Relationship of adverse event to the therapeutic intervention was determined by the study team based on temporal proximity, participant attribution, and clinical judgment. Events deemed probably or definitely related are counted here.

^f^ Blood pressure and heart rate were monitored regularly throughout target quit date in the psilocybin arm but not assessed in NRT arm except at baseline screening. Elevated blood pressure is noted if systolic blood pressure was greater than 160 mm Hg or diastolic blood pressure was greater than 100 mm Hg; heart rate is noted as elevated if greater than 110/min or when the participant noted “heart racing.”

### Smoking Cessation Outcomes

**Figure 3.**
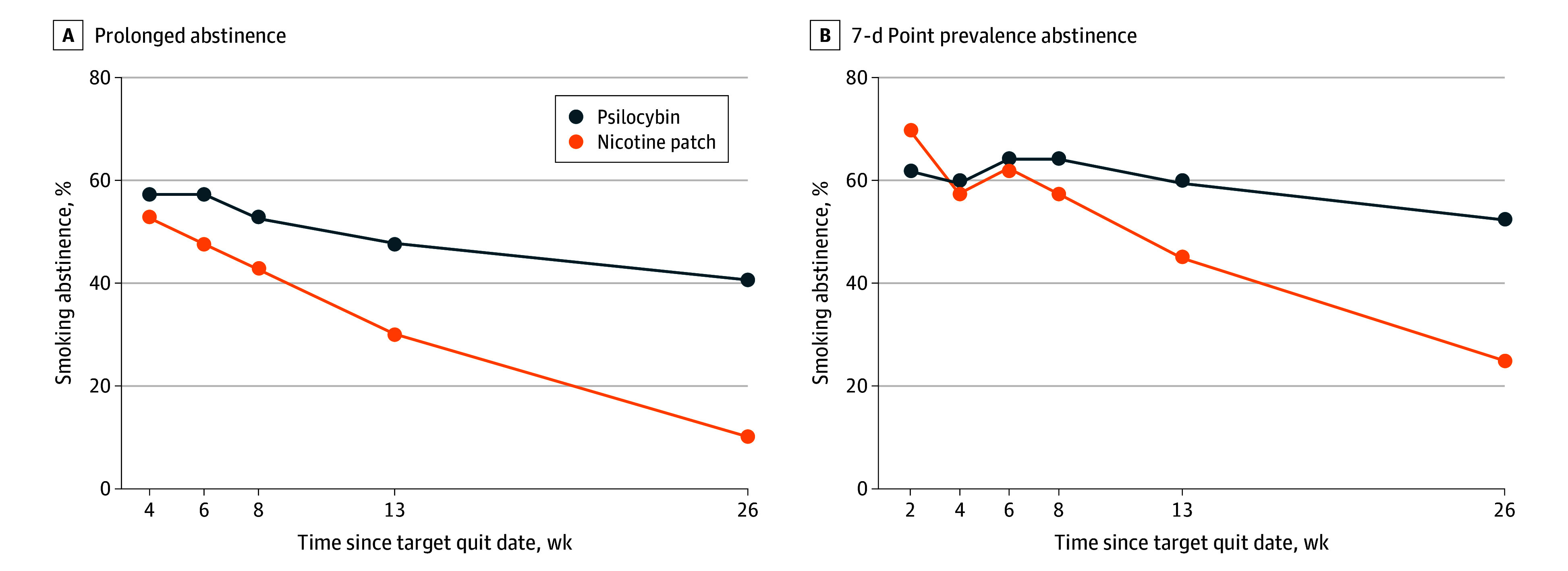
Smoking Abstinence Rates Between the Psilocybin and Nicotine Patch Groups A. Prolonged abstinence, which allowed for an initial 2-week grace period. B. Seven-day point prevalence abstinence in the week preceding each study visit. The number of individuals who completed each time point was 76 at week 2, 75 at week 4, 74 at week 6, 74 at week 8, 74 at week 13, and 68 at week 26. However, consistent with intention-to-treat analysis, all proportions reported here are based on the total randomized sample (N = 82), and those who did not complete a given time point were counted nonabstinent.

On the day after the target quit date, 38 psilocybin participants (90.5%) and 32 nicotine patch participants (80%) self-reported achieving 24 hours of abstinence. At 6-month follow-up, 17 participants (40.5%) in the psilocybin group exhibited biochemically verified prolonged abstinence compared with 4 participants (10.0%) in the nicotine patch group. At 6-month follow-up, 22 participants (52.4%) receiving psilocybin exhibited biochemically verified 7-day point prevalence abstinence compared with 10 participants (25.0%) using the nicotine patch (Figure 3). At 6 months, logistic regression indicated that the psilocybin group had more than 6 times greater odds of prolonged abstinence (OR, 6.12; 95% CI, 1.99-23.26; *P* = .003) and more than 3 times greater odds of 7-day point prevalence abstinence (OR, 3.30; 95% CI, 1.32-8.70; *P* = .01). Generalized linear mixed-effects models of daily cigarette use between the target quit date and 6-month follow-up, conducted as an exploratory analysis, indicated a significant treatment effect (incidence rate ratio, 0.04; 95% CI, 0.004-0.27; *P* = .002), with lower model-predicted use in the psilocybin group. A zero-inflated model provided better fit (Akaike Information Criterion change, 4387; χ^2^_1_ = 4389; *P* < .001), supporting a distinct abstinence-related process. Model-predicted CPD was 1.69 (95% CI, 1.63-1.75) for psilocybin and 3.64 (95% CI, 3.56-3.73) for nicotine patch, reflecting a 53.7% difference between groups. Model-predicted and mean (SD) observed values (1.79 [3.32] in the psilocybin group and 3.73 [4.18] in the nicotine patch group) were closely aligned. Full model results are reported in eAppendix 1 in Supplement 1. Additionally, 53 participants (64.6%) reported prior psychedelic use, which is far higher than recent nationally representative data exhibiting a prevalence of 13.8%.^32^ Exploratory analysis suggested prolonged abstinence did not differ based on prior lifetime psychedelic use (eAppendix 1 in Supplement 1).

## Discussion

A single psilocybin dose combined with manualized CBT yielded significantly greater smoking abstinence than the nicotine patch paired with the same CBT. At 6 months, the psilocybin group had more than 6 times greater odds of showing prolonged abstinence (primary outcome) and more than 3 times greater odds of showing 7-day point prevalence abstinence (secondary outcome). Participants in the psilocybin group smoked a mean of approximately 50% fewer CPD between the target quit date and 6-month follow-up.

Psilocybin appeared safe using established guidelines,^33^ with no serious AEs. Study-related AEs were clinically nonsignificant, well-managed, and largely expected psilocybin effects, such as transiently increased blood pressure, headache, and nausea. Psychedelic therapy requires few administrations, largely limiting AEs to supervised sessions. In contrast, typical smoking cessation medications are used daily for multiple weeks,^34^ resulting in potential for delayed AEs and adherence issues.^35^

The nicotine patch group showed abstinence rates consistent with the literature.^5,16^ A recent meta-analysis^36^ found that standalone NRT resulted in 6-month prolonged abstinence rates of approximately 8% and 7-day point prevalence abstinence rates of 20%. The current study found a 10% prolonged abstinence rate and a 25% 7-day point prevalence abstinence rate, suggesting comparison condition credibility. Whereas nicotine patches confer an estimated OR of 1.37 compared with placebo for achieving smoking abstinence,^5^ in this study the psilocybin group had an OR of 6.1 for 6-month prolonged abstinence compared with nicotine patch participants, although these results require further validation due to the small sample size.

The results of this study add to the increasing evidence that psychedelic treatment may have general antiaddiction efficacy across various addictive drugs.^9,10,37,38,39^ Psilocybin’s lack of direct interaction with nicotinic acetylcholine receptors (or receptors mediating the effects of other addictive drugs) highlights psychedelic therapy as a unique approach wherein the pharmacotherapy does not directly alter drug reinforcement or withdrawal but may instead act via higher-order psychological systems, such as changes in self-concept^40^ and enhanced psychological flexibility.^41,42^ Such mechanisms may also account for transdiagnostic benefits of psychedelic therapies (eg, for depression and anxiety).^43^ These psychological changes are likely associated with corresponding biological processes, just as there are presumably biological changes associated with successful psychotherapy. However, these biological processes are probably of a different nature and more difficult to characterize than those of traditional pharmacotherapies.^44^

### Limitations

This study has several limitations. In this nonblinded study, expectancy could have contributed to positive outcomes. One study found that expectancy predicted antidepressant response to escitalopram but not psilocybin.^45^ We judged this unblinded comparative efficacy design, the reference standard for psychotherapy research, to be the best approach to assessing outcomes based on research showing extremely poor blinding integrity across double-blind psychedelic trials.^15^ One double-blind study of psilocybin for alcohol dependence found that 94% to 95% of participants correctly guessed treatment assignment.^37^ Other psilocybin studies^46,47^ have used low-dose psilocybin or other drugs as comparators, although the success of these in maintaining functional blinding has not been demonstrated. Future double-blind studies in larger samples could help improve precision in estimated generalizable outcomes.

Another limitation is sample generalizability. The sample was low in ethnoracial diversity. Moreover, the sample was highly educated with high intelligence, which could result in greater success, although the 2 groups were well matched on these characteristics, suggesting they did not contribute to differential efficacy. The relatively high level of study retention (>82% overall) suggests a highly motivated sample interested in psychedelic treatment, although it may also reflect clinical interest and feasibility of psychedelic treatments. Additionally, 64.6% of participants reported prior psychedelic use, far higher than recent nationally representative data exhibiting a prevalence of 13.8%.^32^ This finding suggests potential recruitment bias and nongeneralizability. However, exploratory results indicated efficacy was unrelated to prior psychedelic use. Nonetheless, the high rates of prior psychedelic use may indicate hesitancy among individuals without such history to engage in psychedelic-assisted therapies, possibly limiting uptake and use of these treatments.

Because both groups received CBT, this study could not inform the contribution or necessity of psychotherapy.^48^ Future research should address the role of psychotherapy in psilocybin treatment of addictions, including potential synergistic effects, and examine manualized CBT treatment adherence, which was not systematically assessed in this study. It is feasible that psilocybin would be efficacious with less intensive therapeutic support, improving scalability and accessibility. Factorial designs could investigate this. The initial single-arm pilot study^11^ reported 7-day point prevalence abstinence rates of 80% and observed prolonged abstinence in 53.3% of the sample at the 6-month follow-up. In the current trial, the psilocybin group exhibited 7-day point prevalence abstinence rates of 52.4% and prolonged abstinence of 40.5% at the 6-month follow-up. The more intensive intervention used in the single-arm pilot (ie, 15-weekly CBT visits with 2-3 high doses of psilocybin) vs the current randomized pilot (ie, 10 CBT visits during 13 weeks with 1 high dose of psilocybin) could have contributed to greater efficacy. We used 1 psilocybin dose for scalability, for elegance of pre-post comparisons, and because self-report in the pilot suggested the first dose was sufficient in achieving abstinence for most participants. Research in larger samples can generate generalizable outcome data and determine optimal dosing frequency and therapeutic support. Another limitation of the current study is the nicotine patch comparator instead of medications with somewhat greater efficacy (eg, varenicline and combination NRT). However, varenicline requires administration before the target quit date, which constituted a confound for pre-post fMRI analyses not reported in this study. Future research may consider more robust comparators.

Another consideration is that psilocybin treatment is intensive. The smoking cessation field has largely moved toward easily disseminated treatments, such as over-the-counter NRT, brief counseling, and mobile health interventions.^49^ Although these are valuable, their modest outcomes underscore the importance of novel interventions. The morbidity and mortality for tobacco smoking suggest intensive treatments are appropriate, consistent with intensive treatments for other high-morbidity conditions, such as depression and posttraumatic stress disorder. Future work investigating shorter-acting psychedelics could reduce time and cost. Regarding treatment intensity, the psilocybin group received greater contact time, including time with facilitators and study investigators, potentially contributing to increased efficacy. Future studies should equalize contact time between groups and minimize unnecessary interactions. The extended duration of enrollment and data collection was due to not only COVID-19 but also modest grant support, which limited staffing and precluded participant compensation. Nevertheless, findings support accelerating development of psychedelic therapies for substance use disorders, including tobacco. Key questions, such as optimizing treatment parameters, cost-effectiveness, and scalability, remain to be examined.

## Conclusions

In this pilot randomized clinical trial, administration of 1 dose of psilocybin with manualized CBT compared with nicotine patch treatment with CBT significantly increased long-term abstinence. The current trial’s results suggest that psilocybin is a promising candidate for smoking cessation that should move forward in the FDA process toward potential approval.

## Notes

Supplement 1. **eAppendix 1. **Supplementary Methods and Results **eAppendix 2. **Study Inclusion and Exclusion Criteria **eTable 1.** Relative Assignment of Primary and Co-Facilitators Between Groups and by Abstinence Status **eTable 2.** Adverse Events (AEs) Occurring From Target-Quit-Date (TQD) Through 6-Month Follow-Up **eTable 3. **Summary of Study Treatment Visits (Excluding Screening and Follow-Ups) Compared by Treatment Group Including Total Contact Duration in Minutes **eFigure 1.** Mean (SEM) Heart Rate, Systolic, and Diastolic Blood Pressure Readings at Baseline (Pre-Drug Administration) and 30- to 60-Minute Intervals Post-Dosing **eFigure 2. **Smoking Abstinence Rates Between the Psilocybin and Nicotine Patch Groups With 95% Confidence Intervals

Supplement 2.
Trial Protocol

Supplement 3.
Data Sharing Statement
