# Supplementary material for: Psilocybin or Nicotine Patch for Smoking Cessation: A Pilot Randomized Clinical Trial
Source: JAMA Netw Open. 2026 Mar 10;9(3):e260972. doi: 10.1001/jamanetworkopen.2026.0972 (PMC12976795; doi:10.1001/jamanetworkopen.2026.0972)
Supplement: Supplement 1. — eAppendix 1. Supplementary Methods and Results eAppendix 2. Study Inclusion and Exclusion Criteria eTable 1. Relative Assignment of Primary and Co-Facilitators Between Groups and by Abstinence Status eTable 2. Adverse Events (AEs) Occurring From Target-Quit-Date (TQD) Through 6-Month Follow-Up eTable 3. Summary of Study Treatment Visits (Excluding Screening and Follow-Ups) Compared by Treatment Group Including Total Contact Duration in Minutes eFigure 1. Mean (SEM) Heart Rate, Systolic, and Diastolic Blood Pressure Readings at Baseline (Pre-Drug Administration) and 30- to 60-Minute Intervals Post-Dosing eFigure 2. Smoking Abstinence Rates Between the Psilocybin and Nicotine Patch Groups With 95% Confidence Intervals [file jamanetwopen-e260972-s001.pdf]

Supplemental Online Content

Johnson MW, Naudé GP, Hendricks PS, Garcia-Romeu A. Psilocybin or nicotine patch for smoking cessation: a pilot randomized clinical trial. *JAMA Netw Open*. 2026;9(3):e260972. doi:10.1001/jamanetworkopen.2026.0972

eAppendix 1. Supplementary Methods and Results
eAppendix 2. Study Inclusion and Exclusion Criteria
eTable 1. Relative Assignment of Primary and Co-Facilitators Between Groups and by Abstinence Status
eTable 2. Adverse Events (AEs) Occurring From Target-Quit-Date (TQD) Through 6-Month Follow-Up
eTable 3. Summary of Study Treatment Visits (Excluding Screening and Follow-Ups) Compared by Treatment Group Including Total Contact Duration in Minutes
eFigure 1. Mean (SEM) Heart Rate, Systolic, and Diastolic Blood Pressure Readings at Baseline (Pre-Drug Administration) and 30- to 60-Minute Intervals Post-Dosing
eFigure 2. Smoking Abstinence Rates Between the Psilocybin and Nicotine-Patch Groups With 95% Confidence Intervals

This supplemental material has been provided by the authors to give readers additional information about their work.

© 2026 Johnson MW et al. *JAMA Network Open*.

eAppendix 1. Supplementary Methods and Results

Smoking Cessation Outcomes with Other Non-Combustible Nicotine Product Use Coded as Non-Abstinent

When coding other forms of non-combustible nicotine product use as non-abstinent, prolonged abstinence at the 6-month follow-up was still shown by 17 participants (40.5%) in the psilocybin group and went from 4 to 3 participants (7.5%) in the nicotine patch group. Under this criterion, 7-day point-prevalence abstinence went from 22 to 21 participants (50.0%) in the psilocybin group and from 10 to 9 participants (22.5%) in the nicotine patch group. Logistic regression indicated participants in the psilocybin group with no other forms of nicotine use had over 8 times greater odds of prolonged abstinence at the 6-month follow-up (odds ratio [OR], 8.39; 95% CI, 2.50–38.70; P = .002) and over 3 times greater odds of 7-day point-prevalence abstinence (OR, 3.44; 95% CI, 1.35–9.32; P = .01).

Other nicotine use resulting in non-abstinent classification included e-cigarettes/vaping (*n* = 2) and lozenges (*n* = 1).

Prolonged Abstinence by Prior Psychedelic Use Within Each Treatment Group

There were no statistically significant differences in 6-month prolonged abstinence by prior psychedelic use in either treatment group. Prolonged abstinence values represent the number and percentage of participants who maintained abstinence at the 6-month follow-up. Subgroups are defined by any prior psychedelic use. In the psilocybin group, abstinence rates were 33.3% (5 of 15) among participants without prior use and 44.4% (12 of 27) among those with prior use (OR, 1.60; 95% CI, 0.43 to 5.96; P = .53). In the nicotine replacement therapy group, 7.1% (1 of 14) of participants without prior use maintained abstinence compared with 11.5% (3 of 26) of those with prior use (OR, 1.70; 95% CI, 0.16 to 18.02; P > .99). Odds ratios

© 2026 Johnson MW et al. *JAMA Network Open*.

were calculated from contingency tables, and P values were derived from Fisher's exact tests comparing prolonged abstinence by prior use status within each treatment group.

Exploratory Analysis of Daily Cigarette Use Between Target-Quit-Date and 6-Month Follow-up

eMethods

Daily cigarette use between the target-quit-date (TQD) and 6-month follow-up was evaluated in an exploratory analysis using two generalized linear mixed-effects models (GLMMs), each assuming a negative binomial distribution with a log link to account for overdispersed count data. The outcome was daily cigarette use based on the Timeline Follow-Back (TLFB), modeled as a rate with a log-transformed offset for each participant's total number of follow-up days. Analyses were restricted to participants (n = 68) who provided TLFB data extending at least through the 6-month follow-up period, to ensure coverage of the full analysis window. Treatment group (psilocybin vs. nicotine patch) was entered as the primary fixed effect, with baseline cigarettes per day (CPD) included as a covariate to adjust for pretreatment smoking levels. A random intercept for participant ID was included to account for within-subject correlation across repeated observations.

The second model extended the first by adding an intercept-only zero-inflation component to account for excess zeros likely due to sustained abstinence rather than random non-use. Both models were estimated using the glmmTMB package (v1.1.10) in R and compared using Akaike Information Criterion (AIC) and likelihood ratio testing. Model-predicted CPD was estimated as the mean of the fitted values across all participant days, yielding group-level means that account for both within- and between-subject variability. Group-level 95% confidence intervals (CIs) for model-predicted CPD and percent reduction were obtained via nonparametric bootstrapping (10 000 replicates) using the boot package (v1.3-31) in R, applying

© 2026 Johnson MW et al. *JAMA Network Open*.

bias-corrected and accelerated (BCa) adjustment. All tests were 2-tailed with a type I error rate of .05.

eResults

A total of 68 participants were included in the daily cigarette use analysis based on availability of TLFB data through the 6-month follow-up. The first negative binomial GLMM revealed a significant effect of treatment group (incidence rate ratio [IRR], 0.03; 95% CI, 0.003 to 0.25; P = .001) on daily cigarette use. Model-predicted CPD was 52.2% lower for the psilocybin group (1.83; 95% BCa CI, 1.75 to 1.91) compared with the nicotine patch group (3.82; 95% BCa CI, 3.72 to 3.93). The participant-level random intercept had a standard deviation (SD) of 4.0, indicating substantial individual variability. The second, zero-inflated model provided significantly better fit than the standard negative binomial GLMM (ΔAIC = 4387; χ^2_1 = 4389; *P* < .001). The zero-inflation term was also significant (b = -0.55; 95% CI, -0.60 to -0.50; *P* < .001), consistent with a distinct process (e.g., prolonged abstinence) contributing to non-smoking days rather than random variability. The treatment effect remained significant in this model (IRR, 0.04; 95% CI, 0.004 to 0.27; *P* = .002). Model-predicted CPD was 53.7% lower for psilocybin (1.69; 95% BCa CI, 1.63 to 1.75) than for nicotine patch (3.64; 95% BCa CI, 3.56 to 3.73). The random intercept variance was similar to the non-zero-inflated model (SD = 4.01). Predicted and mean (SD) observed values were closely aligned (psilocybin: 1.79, SD = 3.32; nicotine patch: 3.73, SD = 4.18) were closely aligned.

© 2026 Johnson MW et al. *JAMA Network Open*.

eAppendix 2. Study Inclusion and Exclusion Criteria

General Inclusion Criteria:

- 21 to 80 years old
- Have given written informed consent
- Read, write, and speak English
- Be a daily smoker with multiple (i.e., ≥ 2) unsuccessful previous quit attempts, and report a continued desire to quit smoking
- Agree to abstain from smoking for the psilocybin session from 1 hour before psilocybin administration until at least 30 hours afterwards
- Agree to consume approximately the same amount of caffeine-containing beverage (e.g., coffee, tea) that he/she consumes on a usual morning, before arriving at the research unit on the morning of drug session day. If the volunteer does not routinely consume caffeinated beverages, he or she must agree not to do so on session day
- Agree to refrain from using any psychoactive drugs, including alcoholic beverages, within 24 hours of psilocybin administration. Exceptions include caffeine and nicotine
- Be healthy as determined by screening for medical problems via a personal interview, a medical questionnaire, a physical examination, an electrocardiogram (ECG), and routine medical blood and urinalysis laboratory tests
- Agree to abstain from smoking for 24-hours prior to study visits in week 2 and 5

General medical exclusion criteria:

- Women who are pregnant (positive pregnancy test) or nursing, or are not practicing an effective means of birth control
- Cardiovascular conditions: uncontrolled hypertension with resting blood pressure systolic >150 or diastolic >95, angina, a clinically significant ECG abnormality (e.g., atrial fibrilation), TIA in the last 6 months stroke, peripheral or pulmonary vascular disease
- Epilepsy with history of seizures
- Insulin-dependent diabetes; if taking oral hypoglycemic agent, then no history of hypoglycemia
- Currently taking psychoactive prescription medication on a regular basis
- Currently taking on a regular (e.g., daily) basis any medications having a primary centrally-acting pharmacological effect on serotonin neurons or medications that are MAO inhibitors.

© 2026 Johnson MW et al. *JAMA Network Open*.

For individuals who have intermittent or PRN use of such medications, psilocybin sessions will not be conducted until at least 5 half-lives of the agent have elapsed after the last dose.

- Have HIV or Syphilis.
- Have any current neurological illnesses including, but not limited to, seizure disorders, frequent migraines or on prophylaxis, multiple sclerosis, movement disorders, history of significant head trauma, or CNS tumor.
- BMI <16 or ≥40 as determined by medical examination.
- Recent (within past 12 months) or extensive history of hallucinogen use (>20 lifetime uses).
- Smoke <5 cigarettes daily on average.

Psychiatric Exclusion Criteria:

- Current or past history of meeting DSM-IV criteria for Schizophrenia, Psychotic Disorder (unless substance-induced or due to a medical condition), or Bipolar I or II Disorder.
- Current or past history within the last 5 years of meeting DSM-IV criteria for alcohol or drug dependence (excluding caffeine and nicotine) or severe major depression.
- Have a first or second degree relative with schizophrenia, psychotic disorder (unless substance induced or due to a medical condition), or bipolar I or II disorder.
- Currently meets DSM-IV criteria for Dissociative Disorder, Anorexia Nervosa, Bulimia Nervosa, Major Depression, or Post-traumatic stress disorder.

Inclusion criteria for MRI scanning eligibility only:

- 21 to 65 years old
- Agree to abstain from smoking and alcohol for 24-hours, and caffeine for 12-hours prior to MRI scanning
- Be right-handed

General medical exclusion criteria for MRI scanning eligibility only:

- Not suitable to undergo an MRI session due to certain implanted devices (cardiac pacemaker or neurostimulator, some artificial joints, metal pins, surgical clips or other implanted metal parts), body morphology, or claustrophobia.

© 2026 Johnson MW et al. *JAMA Network Open*.

eTable 1. Relative Assignment of Primary and Co-Facilitators Between Groups and by Abstinence Status

Primary Facilitators	Total<br>Participants, n (%)	Psilocybin<br>Participants, n (%)	Nic. Patch<br>Participants, n (%)	Psilocybin Prolonged Abstinence, n (%)	Nic. Patch Prolonged Abstinence, n (%)	Total Prolonged Abstinence, n (%)
1 *	75 (91.5)	40 (95.2)	35 (87.5)	16 (40)	3 (8.6)	19 (25.3)
2 *	7 (8.5)	2 (4.8)	5 (12.5)	1 (50)	1 (20)	2 (28.6)
**Co-Facilitators**	**Total<br>Participants, n (%)**	**Psilocybin<br>Participants, n (%)**	**Nic. Patch Participants, n (%)**	**Psilocybin Prolonged Abstinence, n (%)**	**Nic. Patch Prolonged Abstinence, n (%)**	**(n) Total Prolonged Abstinence, n (%)**
3	21 (25.6)	12 (28.6)	9 (22.5)	3 (25.0)	0 (0)	3 (14.3)
4	32 (39.0)	15 (35.7)	17 (42.5)	9 (60)	2 (11.8)	11 (34.4)
2	10 (12.2)	6 (14.3)	4 (10)	2 (33.3)	0 (0)	2 (20)
5	8 (9.8)	5 (11.9)	3 (7.5)	2 (40)	0 (0)	2 (25.0)
6/4 [a]	3 (3.7)	1 (2.4)	2 (5.0)	0 (0)	1 (50)	1 (33.3)
5/7 [a]	2 (2.4)	2 (4.8)	NA	0 (0)	NA	0 (0)
3/8 [a]	1 (1.2)	NA	1 (2.5)	NA	0 (0)	0 (0)
9	2 (2.4)	NA	2 (5.0)	NA	1 (50)	1 (50)
10	1 (1.2)	NA	1 (2.5)	NA	0 (0)	0 (0)
11	2 (2.4)	1 (2.4)	1 (2.5)	1 (100)	0 (0)	1 (50)

* Denotes primary doctoral or master's level facilitator.
[a] Six participants (n=3 psilocybin and n=3 nicotine patch) had 2 co-facilitators working with them at various times due to staff turnover and new staff training.

© 2026 Johnson MW et al. *JAMA Network Open*.

eTable 2. Adverse Events (AEs) Occurring From Target-Quit-Date (TQD) Through 6-Month Follow-Up

System Class	Preferred Term	Psilo., n (%)	Psilo., TQD, n	Psilo, Related, n	NRT, n (%)	NRT, TQD, n	NRT, Related, n	Total, n (%)
Cardiovascular	hypertension	36 (85.7)	36	36	0 (0)	0	0	36 (43.9)
Nervous system	headache	22 (52.4)	20	20	4 (10)	3	4	26 (31.7)
Psychiatric	anxiety	9 (21.4)	1	1	12 (30)	0	0	21 (25.6)
Psychiatric	depression	12 (28.6)	1	4	9 (22.5)	0	3	21 (25.6)
Psychiatric	insomnia	3 (7.1)	0	0	8 (20)	3	7	11 (13.4)
Respiratory	Nasopharyngitis	4 (9.5)	0	0	5 (12.5)	0	0	9 (11.0)
Musculoskeletal	back pain	4 (9.5)	2	2	3 (7.5)	0	0	7 (8.5)
Skin and subcutaneous tissue	rash	0 (0)	0	0	7 (17.5)	1	7	7 (8.5)
Psychiatric	irritability	4 (9.5)	1	0	2 (5)	0	0	6 (7.3)
Psychiatric	vivid dreams	0 (0)	0	0	6 (15)	4	6	6 (7.3)
Gastrointestinal	nausea	3 (7.1)	3	3	3 (7.5)	0	2	6 (7.3)
Gastrointestinal	gastroenteritis	6 (14.3)	1	1	0 (0)	0	0	6 (7.3)
General	fatigue	3 (7.1)	3	3	2 (5)	0	2	5 (6.1)
Gastrointestinal	food poisoning	2 (4.8)	0	0	2 (5)	0	0	4 (4.9)
Eye	visual disturbance	4 (9.5)	1	3	0 (0)	0	0	4 (4.9)
Cardiovascular	tachycardia	6 (14.3)	6	6	1 (2.5)	0	1	3 (3.7)
Nervous system	migraine	2 (4.8)	1	1	1 (2.5)	0	0	3 (3.7)
Nervous system	concentration impaired	2 (4.8)	0	2	1 (2.5)	0	1	3 (3.7)
Musculoskeletal	shoulder pain	1 (2.4)	1	0	2 (5)	0	0	3 (3.7)
Musculoskeletal	fracture	2 (4.8)	0	0	1 (2.5)	0	0	3 (3.7)
Gastrointestinal	diarrhea	1 (2.4)	0	0	2 (5)	0	0	3 (3.7)
Psychiatric	emotional distress	1 (2.4)	1	1	1 (2.5)	0	0	2 (2.4)
Musculoskeletal	arthralgia	0 (0)	0	0	2 (5)	1	1	2 (2.4)

© 2026 Johnson MW et al. *JAMA Network Open*.

Musculoskeletal	neck pain	2 (4.8)	1	0	0 (0)	0	0	2 (2.4)
Gastrointestinal	constipation	1 (2.4)	0	0	1 (2.5)	0	0	2 (2.4)
Respiratory	upper respiratory tract infection	0 (0)	0	0	2 (5)	0	0	2 (2.4)
Respiratory	sinusitis	1 (2.4)	0	0	1 (2.5)	0	0	2 (2.4)
Respiratory	lower respiratory tract infection	1 (2.4)	0	0	1 (2.5)	0	0	2 (2.4)
Ear and labyrinth	otitis	0 (0)	0	0	2 (5)	0	0	2 (2.4)
General	fever	0 (0)	0	0	2 (5)	0	0	2 (2.4)
Cardiovascular	palpitations	0 (0)	0	0	1 (2.5)	0	1	1 (1.2)
Cardiovascular	chest discomfort	1 (2.4)	0	0	0 (0)	0	0	1 (1.2)
Psychiatric	nicotine craving	1 (2.4)	1	0	0 (0)	0	0	1 (1.2)
Nervous system	dizziness	1 (2.4)	0	0	0 (0)	0	0	1 (1.2)
Nervous system	tremor	0 (0)	0	0	1 (2.5)	1	1	1 (1.2)
Musculoskeletal	spinal stenosis	1 (2.4)	0	0	0 (0)	0	0	1 (1.2)
Musculoskeletal	knee pain	0 (0)	0	0	1 (2.5)	1	0	1 (1.2)
Musculoskeletal	leg pain	1 (2.4)	1	1	0 (0)	0	0	1 (1.2)
Musculoskeletal	myalgia	0 (0)	0	0	1 (2.5)	0	1	1 (1.2)
Musculoskeletal	meniscus tear	0 (0)	0	0	1 (2.5)	0	0	1 (1.2)
Musculoskeletal	hand pain	1 (2.4)	0	0	0 (0)	0	0	1 (1.2)
Musculoskeletal	arthritis	0 (0)	0	0	1 (2.5)	1	0	1 (1.2)
Gastrointestinal	diverticulitis	0 (0)	0	0	1 (2.5)	0	0	1 (1.2)
Gastrointestinal	cholelithiasis	0 (0)	0	0	1 (2.5)	0	0	1 (1.2)
Gastrointestinal	gastritis	0 (0)	0	0	1 (2.5)	0	0	1 (1.2)
Gastrointestinal	polyp	1 (2.4)	0	0	0 (0)	0	0	1 (1.2)
Gastrointestinal	abdominal pain	1 (2.4)	1	1	0 (0)	0	0	1 (1.2)
Respiratory	pharyngitis	0 (0)	0	0	1 (2.5)	0	0	1 (1.2)
Respiratory	sore throat	0 (0)	0	0	1 (2.5)	0	0	1 (1.2)
Respiratory	dyspnea	1 (2.4)	0	0	0 (0)	0	0	1 (1.2)

© 2026 Johnson MW et al. *JAMA Network Open*.

Respiratory	allergic rhinitis	0 (0)	0	0	1 (2.5)	0	0	1 (1.2)
Respiratory	cough	1 (2.4)	0	0	0 (0)	0	0	1 (1.2)
Ear and labyrinth	vertigo	1 (2.4)	0	0	0 (0)	0	0	1 (1.2)
Eye	photopsia	1 (2.4)	0	0	0 (0)	0	0	1 (1.2)
General	dysgeusia	0 (0)	0	0	1 (2.5)	0	0	1 (1.2)
General	decreased appetite	1 (2.4)	0	0	0 (0)	0	0	1 (1.2)
Injury, poisoning, and procedural complications	hand injury	0 (0)	0	0	1 (2.5)	0	0	1 (1.2)
Injury, poisoning, and procedural complications	knee injury	0 (0)	0	0	1 (2.5)	0	0	1 (1.2)
Oral and dental	trismus	0 (0)	0	0	1 (2.5)	0	0	1 (1.2)
Oral and dental	dental abscess	1 (2.4)	0	0	0 (0)	0	0	1 (1.2)
Oral and dental	dental injury	0 (0)	0	0	1 (2.5)	0	0	1 (1.2)
Surgical and medical	hernia	0 (0)	0	0	1 (2.5)	0	0	1 (1.2)
Reproductive	decreased libido	1 (2.4)	0	0	0 (0)	0	0	1 (1.2)
Pregnancy, puerperium, and perinatal	spontaneous abortion	1 (2.4)	0	0	0 (0)	0	0	1 (1.2)
Investigations	elevated prostate-specific antigen	0 (0)	0	0	1 (2.5)	0	0	1 (1.2)
Infections and infestations	urinary tract infection	0 (0)	0	0	1 (2.5)	0	0	1 (1.2)

Note: Supplemental Table 2 lists frequency and percentage of participants experiencing adverse events for all adverse events occurring during the 6-month study period following the first administration of study medication. Adverse events are classified by MedDRA System Class (SOC) and preferred term (PT). Frequency of AEs that occurred on the TQD are noted here by treatment group. Frequency of AEs that were deemed definitely or probably related to study medications is also noted here.

© 2026 Johnson MW et al. *JAMA Network Open*.

eTable 3. Summary of Study Treatment Visits (Excluding Screening and Follow-Ups) Compared by Treatment Group Including Total Contact Duration in Minutes

Visit (week)	Visit Duration in Minutes		Mann-Whitney U test [a]
	Psilocybin	Nicotine Patch	
Visit 1 (week 1), mean (SD) [b]	194.4 (75.4)	176.5 (78.2)	U = 714, p = 0.25
Visit 2 (week 2), mean (SD) [b]	249.6 (117.2)	218.3 (132.9)	U = 724, p = 0.28
Visit 3 (week 3), mean (SD)	103.1 (21.2)	90.0 (24.8)	U = 539, $\boldsymbol{p}$ **= 0.005**
Visit 4 (week 4), mean (SD)	105.9 (26.2)	85.2 (24.0)	U = 456, $\boldsymbol{p}$ **= 0.0003**
Visit 5 (week 5; TQD with psilocybin or patch administration), mean (SD) [b]	517.7 (40.3)	179.7 (114.8)	U = 0, $\boldsymbol{p}$ **< 0.0001**
Integration (week 5), mean SD) [b]	235.4 (120.7)	n/a	n/a
Visit 6 (week 6), mean (SD)	79.4 (18.6)	66.5 (19.1)	U = 527, $\boldsymbol{p}$ **= 0.013**
Visit 7 (week 7), mean (SD)	67.1 (23.0)	62.0 (21.3)	U = 667, p = 0.57
Visit 8 (week 9), mean (SD)	58.5 (20.0)	59.9 (21.6)	U = 661, p = 0.66
Visit 9 (week 11), mean (SD)	55.3 (14.3)	54.2 (18.7)	U = 644, p = 0.66
Visit 10 (week 13), mean (SD)	59.2 (20.9)	56.2 (19.1)	U = 611, p = 0.43
Additional Contact, mean (SD) [c]	22.2 (4.7)	23.3 (11.5)	U = 5, p = 0.5
Total Contact Duration, mean (SD)	1660.2 (403.4)	1035.5 (360.4)	n/a
Total Contact Duration, median (IQR)	1773 (639)	1005 (605)	U = 225, $\boldsymbol{p}$ **< 0.001**

[a] Mann-Whitney U tests were performed to assess differences in contact time between groups at each study visit and for total contact duration.

[b] A subsample of participants underwent functional magnetic resonance imaging (fMRI) before and after treatment with procedures in weeks 1, 2, and 5 included here in visit duration where applicable (i.e., contributing to increased variance in visit duration in these weeks).

[c] Additional contact refers to meetings requested by participants with primary and co-facilitators for further support with smoking abstinence outside the regularly scheduled study visits. Time spent meeting with investigators is included here in the reported duration of the particular visit when that contact occurred.

© 2026 Johnson MW et al. *JAMA Network Open*.

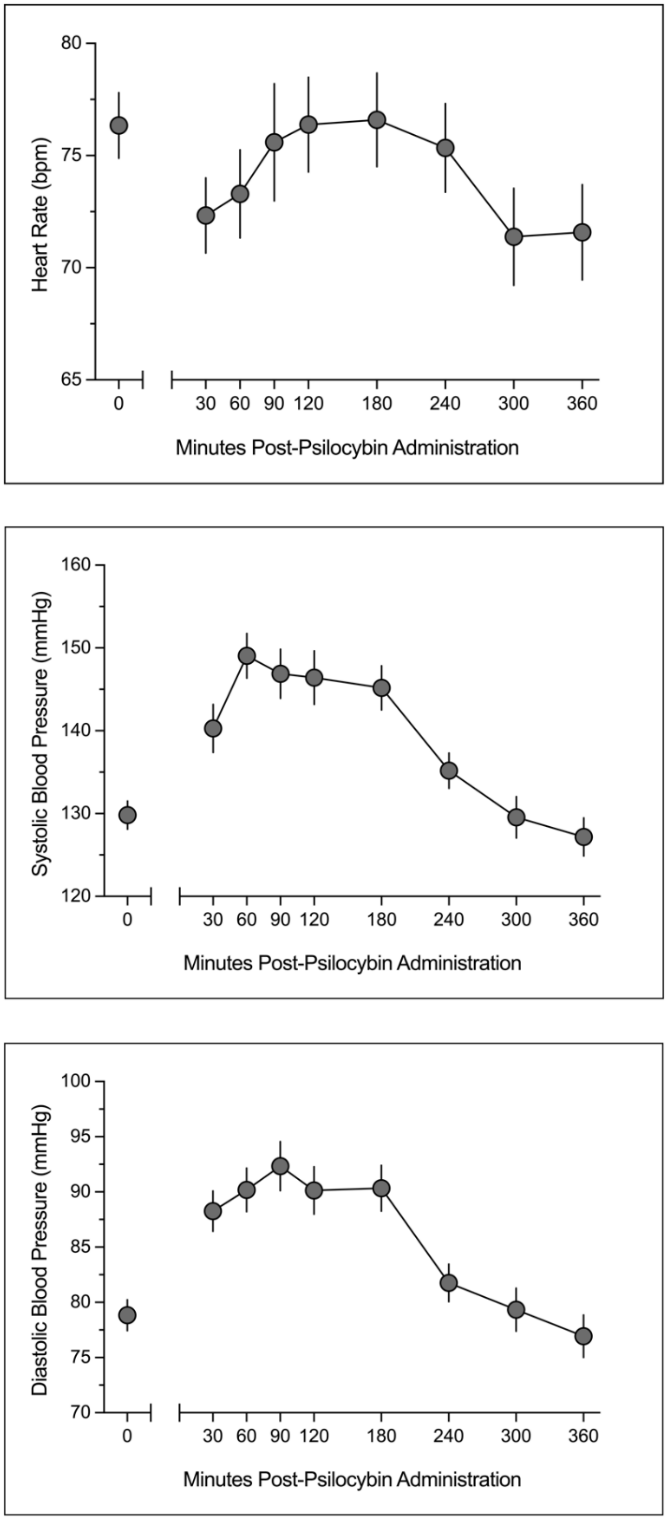

eFigure 1. Mean (SEM) Heart Rate, Systolic, and Diastolic Blood Pressure Readings at Baseline (Pre-Drug Administration) and 30- to 60-Minute Intervals Post-Dosing

© 2026 Johnson MW et al. *JAMA Network Open*.

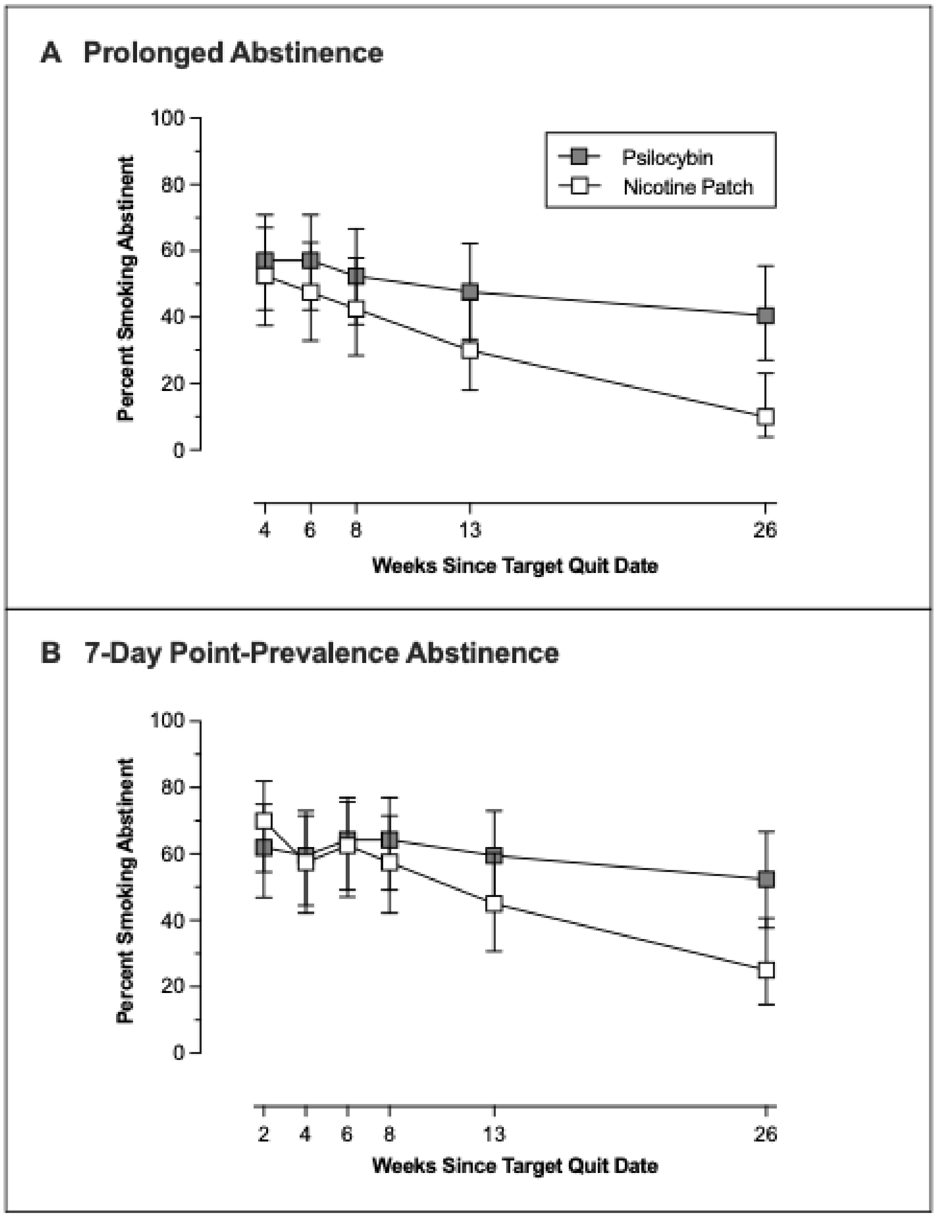

eFigure 2. Smoking Abstinence Rates Between the Psilocybin and Nicotine-Patch Groups With 95% Confidence Intervals

Panel A shows prolonged abstinence which allowed for an initial 2-week grace period. Panel B shows 7-day point-prevalence abstinence in the week preceding each study visit.

Note: Number of individuals who completed each timepoint was (n=76) at week 2, (n=75) at week 4, (n=74) at week 6, (n=74) at week 8, (n=74) at week 13, and (n=68) at week 26. However, consistent with ITT analysis, all proportions reported here are based on the total randomized sample (N=82), and those who did not complete a given timepoint were counted non-abstinent.

© 2026 Johnson MW et al. *JAMA Network Open*.
