# Supplementary material for: Psilocybin or Nicotine Patch for Smoking Cessation: A Pilot Randomized Clinical Trial
Source: JAMA Netw Open. 2026 Mar 10;9(3):e260972. doi: 10.1001/jamanetworkopen.2026.0972 (PMC12976795; doi:10.1001/jamanetworkopen.2026.0972)
Supplement: Supplement 2. — Trial Protocol [file jamanetwopen-e260972-s002.pdf]

Psilocybin-facilitated Smoking Cessation Treatment: A Pilot Study

Johns Hopkins Medicine - eForm A

- **Use the section headings to write the eForm A, inserting the appropriate material in each. If a section is not applicable, leave heading in and insert N/A.**
- **When submitting eForm A (new or revised), enter the date submitted to the field at the top of eForm A.**

****

1. Abstract

a. Provide no more than a one page research abstract briefly stating the problem, the research hypothesis, and the importance of the research.

One of the most promising lines of investigation for the therapeutic use of hallucinogens in the 1960s and 1970s was in the treatment of drug dependence. However, these studies did not involve the rigor and controls expected of modern clinical psychopharmacology research, making the efficacy of this approach difficult to judge. We propose to examine psilocybin administration combined with a structured smoking cessation program in nicotine dependent individuals to provide preliminary data on the efficacy of this combined treatment in smoking cessation. Prior work in our laboratory has shown that under carefully prepared and supportive conditions, psilocybin administration can facilitate highly salient experiences with enduring personal meaning and spiritual significance (Griffiths et al., 2006, 2008, 2011). It is plausible that embedding such highly meaningful experiences into a drug dependence cessation attempt may provide an enduring motivation for remaining abstinent. Cigarette smoking is a good model system for studying drug dependence because users are less likely to be challenged by the many social and economic impairments that often accompany dependence on other drugs such as cocaine, heroin, or alcohol. More specifically, we propose to conduct a pilot study in which psilocybin is administered under highly supportive conditions to individuals who are nicotine-dependent cigarette smokers, who have had multiple unsuccessful quit attempts, and who continue to desire to quit smoking. Other than nicotine dependence, participants will be healthy.

In total, 115 participants will complete this pilot study. Fifteen individuals have already undergone the initially approved protocol with no clinically significant adverse events. A further 100 participants will be randomized to a psilocybin condition or a nicotine replacement therapy (NRT; i.e., transdermal nicotine patch) condition, 60 of whom will be studied with functional neuroimaging procedures. The NRT condition will serve as an important control for evaluating relative efficacy of the psilocybin condition, as well as examining differences in neuroimaging results. After screening and enrollment, individuals who are eligible for MRI scanning (n=60) will be invited to complete an optional preliminary sub-study protocol consisting of a 6-8 hour MRI scan, blood draw, and questionnaire assessment at the National Institute on Drug Abuse (NIDA) Intramural Research Program on the Johns Hopkins Bayview Campus. After completing the sub-study protocol, or opting out, participants will begin a 13-week cognitive-behavioral smoking cessation intervention, with Target Quit Date (TQD) set in approximately week 5. Participants who are eligible for and willing to participate in MRI scanning (n=60), will have MRI scans conducted in weeks 2 and 5, and at 3-month follow-up for those participants who have maintained smoking abstinence to that point. Individuals who do not meet MRI scanning inclusion criteria, but are otherwise eligible to enroll (n=40), will complete only the treatment-related portions of the study.

During the third treatment meeting in week 3, participants will be randomized into one of two treatment conditions (i.e., psilocybin or NRT). In one condition a single high-dose (30mg/70kg) of psilocybin will be administered in the context of a 13-week smoking cessation treatment protocol. In the other condition an 8 to 10 week regimen of NRT (patch) will be administered within the same 13-week treatment protocol. Participants in both conditions will meet weekly with study staff during weeks 1-7, and at two-week intervals during weeks 8-13 (i.e., in weeks 9, 11, and 13) of the 13-week treatment period. All participants will be asked to complete follow-up meetings at 3, 6, and 12 months post-TQD to assess smoking status and complete questionnaires. Additionally, in order to prevent differential dropout driven by individuals who hope to receive the novel psilocybin treatment, participants randomized to the NRT condition will be offered an optional psilocybin (30 mg/70 kg) session after completing their 6-month follow-up.

A number of measures that assess the psychological process of smoking cessation will be utilized at intake and repeatedly after the psilocybin session or initiation of NRT. In order to explore the contribution of spiritual experience to smoking cessation success in the psilocybin treatment condition, questionnaires that assess the spiritual nature of the psilocybin session experiences will be given after the psilocybin session. In order to assess the hypothesis that spiritual experience or smoking cessation is associated with changes in immune function or stress hormones, blood samples at baseline and 1-week post-TQD will be collected from both groups for cytokine and hormonal analyses when possible. Diurnal saliva samples of cortisol will also be assessed at baseline, and 1-week post-TQD. In order to examine neural mechanisms associated with psilocybin-facilitated smoking cessation, MRI data will be compared within-subjects and between treatment groups in a subset of up to 60 participants who complete MRI scanning procedures.

2. Objectives (include all primary and secondary objectives)

Primary objective: This is a pilot study to provide preliminary evidence on the efficacy of psilocybin administration combined with a structured smoking cessation program for the treatment of nicotine dependence. In order to differentiate the role of psilocybin from the role of cognitive-behavioral therapy and support from study staff, a randomized control group will be utilized. Participants in the control condition will receive a matched cognitive-behavioral intervention in conjunction with NRT. The results should also provide information about clinical trends that may be compared with previously published smoking cessation research that utilized cognitive-behavioral therapy or NRT (e.g., Fiore et al., 1994; Stapleton et al., 2013; Sykes & Marks, 2004). Such trends will indicate whether a larger-scale, rigorously controlled experimental study is warranted.

Secondary objective: In addition to providing preliminary information on efficacy, the proposed pilot study will also provide valuable clinical experience for us to continue developing a psilocybin-based clinical intervention for treatment of drug dependence (e.g., smoking cessation). Such clinical experience will consist of both qualitative data from participant interviews and a battery of quantitative outcome measures, most of which are well-validated instruments with demonstrated clinical relevance. These measures will provide data that indicate which factors are theoretically relevant to the proposed intervention, and may assist in the design of further controlled trials if follow up investigation is warranted. In addition to the assessment of mystical-type experience (implicated as important to the attribution of behavioral change in previous research; Garcia-Romeu et al., 2015; Griffiths et al., 2006), relevant factors to be studied include self-efficacy, personal values, temporal perspective, and mood, (which are

known to relate to nicotine dependence or smoking cessation). Outcome measures also include blood and saliva hormonal markers of stress. Blood samples will additionally allow for the assessment of cytokine markers of immune function, which will provide an opportunity to examine the hypothesized link between mystical-type experience and improved immune function (Roberts, 2006). Finally, the collection of MRI data will allow for a preliminary examination of underlying neural mechanisms that may play a role in psilocybin-facilitated smoking cessation, including the intermediate and persisting effects of psilocybin on resting state functional connectivity, and potential brain-based factors that may be useful predictors or mediators of treatment outcome.

3. Background (briefly describe pre-clinical and clinical data, current experience with procedures, drug or device, and any other relevant information to justify the research)

Research Utilizing Classical Hallucinogens in the Treatment of Substance Dependence: A major focus of clinical hallucinogen research in the 1960s and early 1970s was hallucinogen-facilitated treatment of alcoholism and other forms of substance dependence (e.g., Chwelos et al., 1959; Smart et al., 1966; Holister et al., 1969; Ludwig et al., 1969; Kurland et al., 1971; Savage and McCabe, 1973). While some studies psychologically prepared patients and utilized supportive conditions (e.g., Chwelos et al., 1959; Kurland et al., 1971; Savage and McCabe, 1971), others drastically departed from this model (and from the procedures used previously in our laboratory and proposed for the current study), by administering high doses to unprepared, physically-restrained patients (e.g., Smart et al., 1966). Results across studies were ultimately inconclusive due to such variations in methods and a lack of modern controls and experimental rigor (Abuzzahab and Anderson, 1971; McGlothlin and Arnold, 1971; Halpern, 1996; Mangini, 1998). Despite suggestive preliminary findings, follow up with rigorous, well-controlled studies utilizing high levels of patient preparation and interpersonal support was never conducted. Human research with hallucinogens in the United States became virtually dormant in the early 1970s, soon after much of the initial research on hallucinogen-facilitated treatment of substance dependence was published. Importantly, clinical research with hallucinogens became dormant not due to scientific or medical concerns, but due to the withdrawal of funding and professional support in reaction to the drug excesses of the 1960s and the reckless and irresponsible behavior of a very small number of clinical hallucinogen investigators.

Previous Research with Psilocybin by Our Research Team: In a study that was amongst the first to investigate classical hallucinogens in humans for several decades, our laboratory recently conducted and reported a study that safely administered psilocybin under highly-supportive and prepared conditions (Griffiths et al., 2006). These conditions of substantial interpersonal support and preparation for drug effects were similar to the methods utilized by early reports of successful hallucinogen-facilitated substance dependence treatment (e.g., Chwelos et al., 1959; Kurland et al., 1971; Pahnke et al., 1970; Savage and McCabe, 1973). The study in our laboratory with healthy volunteers found that psilocybin administration occasioned mystical-type experiences (as measured with well-validated scales developed in the psychology of religion) that had enduring personal meaning and spiritual significance in the majority of volunteers. Moreover, follow up work indicated that even 14 months after the original study, the majority of participants considered the experience to be among the five most personally meaningful or spiritually significant experiences in their lives (Griffiths et al., 2008). It is plausible that embedding such highly meaningful experiences into a drug dependence cessation attempt may provide an enduring motivation for remaining abstinent. Indeed, several studies have suggested

that increased levels of spirituality are associated with improved treatment outcomes in substance dependence recovery (e.g., Coyle et al., 2006; Piderman et al., 2007; Piedmont, 2004; Zemore, 2007). Unlike other forms of drug dependence, spirituality has not traditionally been addressed in smoking cessation programs. However, a recent survey study in smokers highlighted the importance of studying this domain in nicotine dependence, showing that 78% of smokers indicated that spiritual resources would be helpful in quitting smoking (Gonzales e al., 2007).

Our laboratory's work in healthy volunteers also suggests the possibility that ratings of personal and spiritual meaning may be accompanied by observable, positive behavioral change (Griffiths et al., 2006, 2008, 2011). Ratings by community observers (blinded to drug conditions) approximately 2 months after each session in the original study indicated that, compared to the active placebo methylphenidate, psilocybin was associated with significant increases in positive mood and behavior in the participants. Moreover, at the 14-month follow up, 61% of participants rated that the experience was associated with moderate to extreme positive behavior change. Although participants were not drug dependent, these ratings from participants and from blinded community observers suggest the feasibility of positive behavioral change in the domain of drug dependence. In the current protocol, cigarette smoking was selected as a good model system for studying drug dependence because users are less likely to be challenged by the many social and economic impairments that often accompany dependence on other drugs such as cocaine, heroin, or alcohol. Furthermore, current pilot results from this protocol suggest both feasibility and safety, with 80% of the current sample (*N*=15) showing biologically verified smoking abstinence at 6-month follow-up, and no adverse events stemming from study treatment throughout a total of 42 psilocybin sessions (Johnson et al., 2014).

Other Research Suggesting the Potential Efficacy of Hallucinogen-facilitated Treatment in Drug Dependence: Various anthropological reports have suggested that hallucinogens use within the ritualized practices of indigenous cultures may be efficacious in the treatment of a variety of forms of drug dependence, with reported high rates of recovery and prolonged abstinence. Such reports include those of peyote (which contains the classical hallucinogen mescaline) ceremonies within the Native American Church in the United States (Albaugh & Anderson, 1974; Bergman, 1971; Blum et al., 1977; Calabrese, 1997; Garrity, 2000; Menninger, 1971; Pascarosa et al., 1976; Roy, 1973) and ayahuasca (which contains the classical hallucinogen N,N-dimethyltryptamine or DMT) ceremonies within certain Amazonian societies (Dobkin de Rios et al., 2002). It is not possible to differentiate the role of hallucinogen exposure from the larger context of spiritual guidance and support provided in these settings. Nonetheless, these anecdotal reports are suggestive, and if the hallucinogen does play a critical role in the treatment of drug dependence in such settings, these reports of indigenous cultures may highlight the importance of meaningful structure and a supportive environment in shaping the efficacy of hallucinogens in drug dependence treatment, a point emphasized by several earlier clinical researchers investigating LSD in the treatment of drug dependence (e.g., Chwelos et al., 1959; Kurland et al., 1971; Savage and McCabe, 1973).

Other recent descriptive research suggests that naturalistic examples of dramatic positive behavioral change (such as overcoming intractable drug dependence) are sometimes associated with spontaneously occurring, transformative psychological experiences, frequently of a mystical-type variety (e.g., Miller, 2004; Miller & C'de Baca, 2001). The most well-known of such experiences may be that of Alcoholics Anonymous founder Bill Wilson, whose spontaneously-occurring mystical-type experience led him to stop drinking and provided the inspiration for founding Alcoholics Anonymous (Alcoholics Anonymous, 1984). Such accounts raise the question of whether hallucinogen-facilitated mystical-type experiences, like

spontaneously occurring mystical-type experiences, may be efficacious in overcoming drug dependence. These accounts are also consistent with the observations of early investigators who noted that mystical-type or "peak" experiences in hallucinogen trials for drug dependence may have mediated successful clinical responses (e.g., Kurland et al., 1971; Savage and McCabe, 1973).

General History of Psilocybin in Research: Modern research on psilocybin began with the work of Richard Evans Schultes at Harvard in the 1930s, whose doctoral dissertation documented the use of a species of psychoactive mushrooms among indigenous tribes in Mexico (Schultes 1940). Academic interest in the psychopharmacology of these mushrooms led to their analysis by the medicinal chemist Albert Hofmann and colleagues at Sandoz Laboratories in Basel, Switzerland, where psilocybin and psilocin were first isolated, identified, and reported in 1958 (Hofmann et al. 1958; Hofmann and Troxler 1959; Hofmann 1983).

Sandoz produced two synthetic drugs derived from mushroom-extracted psilocybin, CEY 19 (4-phosphoryloxy-N,N-diethyltryptamine) and CZ-74 (4-hydroxy-N,N-diethyltryptamine), both of which are shorter acting than psilocybin (Baer 1967). Psilocybin itself was made available for research purposes in Europe and the U.S. under the trade name Indocybin. Early research with psilocybin attempted to study the effects of the drug without recognition of the powerful influences of set (psychological state) and setting (interpersonal and physical environment) (e.g. Hollister 1961; Malitz et al. 1960; Rinkel et al. 1960, Rümmele & Gnirss 1961). Subsequent research, which included more preparation and interpersonal support during the period of drug action, found fewer adverse psychological reactions, such as panic reactions and paranoid episodes, and increased reports of positively-valued experiences (Metzner et al. 1965; Pahnke 1969). With few exceptions (e.g. Duke & Keeler 1968; Rynearson et al. 1968; Pahnke 1969) most early studies included no control or comparison groups. Investigations of psilocybin and similar compounds as adjuncts in psychotherapy, notably in "psycholytic therapy," in which a low dose was administered to facilitate the emergence of psychodynamic material, were conducted at several sites in Europe (e.g., Leuner 1962). In the U.S., basic biochemical and psychological research was conducted with psilocybin at such sites as Ohio State University (Landon & Fischer 1970), the University of Minnesota (Rynearson et al. 1968) and the University of Washington (Horita 1963). Research with psilocybin in the psychotherapy of terminal cancer patients and outpatient neurotics was just beginning at the Maryland Psychiatric Research Center in Baltimore when the administrative restructuring and lack of funding rendered that research program dormant in 1976. Renewed research activity with psilocybin began with Strassman's controlled trials in the U.S. (Strassman 2001) and recent studies in Europe (e.g., Hasler et al. 1997, 2004; Vollenweider et al. 1997, 1998, 1999; Gouzoulis-Mayfrank et al. 1999a,b) and the U.S. (Moreno et al., 2006; Griffiths et al., 2006; Grob, 2005).

As reviewed above, psilocybin has been given in numerous clinical studies over the last 40 years. In many studies, maximum doses ranged from about 15 to 36 mg (Malitz et al. 1960; Leary et al. 1963; Metzner et al. 1965; Pahnke 1969; Hasler et al. 1997; Vollenweider et al. 1997, 1998, 1999; Gouzoulis-Mayfrank et al. 1999a,b). The psychological effects of psilocybin include significant alterations in perceptual, cognitive, affective, volitional, and somatesthetic functions, including visual and auditory sensory disturbances, difficulty thinking, mood fluctuations, and dissociative phenomena. Nausea, yawning, restlessness or malaise, sensation of disorientation, nervousness, giddiness, and euphoria have also been reported (e.g. Hollister 1961; Malitz et al. 1960; Rinkel et al. 1960; Vollenweider et al. 1997, 1999; Gouzoulis-Mayfrank et al. 1999b). Physiological effects are minor and have included dilated pupils and increased tendon

reflexes (Isbell 1959; Hollister 1961). Some studies have reported small-magnitude or inconsistent increases in heart rate, blood pressure, and body temperature but these findings are confounded by the mild anxiety reported by participants participating in the studies (Isbell 1959; Malitz et al. 1960; Hollister 1961; Gouzoulis-Mayfrank et al. 1999b). After oral administration, the effects of psilocybin follow an orderly timecourse, with effects peaking at 1.5 to 2 hours and subsiding by 5-7 hours after administration (Malitz et al. 1960; Vollenweider et al. 1997, 1999).

Research on Nicotine Replacement for Smoking Cessation: NRT is a reliable and effective smoking cessation treatment shown to consistently increase rate of quitting by 50 to 70% relative to inert controls (Silagy et al., 2000; Stead et al., 2012), and exhibiting success rates comparable to those of the non-nicotine smoking cessation pharmacotherapy bupropion (Mills et al., 2012; Stapleton et al., 2013). Meta-analyses have found no significant difference in efficacy across NRT methods (e.g., patch, gum, nasal spray, lozenge; Stead et al., 2012), and patches have exhibited the mildest side effects (e.g., rash, skin irritation; Mills et al., 2010). NRT further provides an attractive control treatment as it has been found to exhibit no significant abuse or dependence potential (West et al., 2000). This study will use a transdermal nicotine patch for 8 to 10 weeks according to recommended label usage (For individuals who smoke more than 10 cigarettes per day: 21mg daily for weeks 1-6, 14mg daily for weeks 7-8, 7mg daily for weeks 9-10. For individuals who smoke 10 or less cigarettes per day: 14mg daily for weeks 1-6, 7mg daily for weeks 7-8; FDA label, PDR.net).

4. Study Procedures

a. Study design, including the sequence and timing of study procedures (distinguish research procedures from those that are part of routine care).

Study design: For this revised study protocol we will randomize 80 participants to complete a 13-week cognitive-behavioral therapy treatment protocol involving either a single high-dose (30mg/70kg) administration of psilocybin, or an 8 to 10 week course of NRT (transdermal nicotine patch) for smoking cessation. Participants will be nicotine-dependent cigarette smokers, who have had multiple unsuccessful quit attempts, and who continue to desire to quit smoking. Other than nicotine dependence, participants will be physically and psychologically healthy. In total, 95 participants will complete the study, including 15 individuals who have already completed psilocybin-facilitated treatment before the current protocol modifications, and 80 further individuals to be recruited for the revised study design including NRT control arm. Fifty participants will also undergo MRI data collection before and after treatment to inform neurobiological mechanisms of action. After screening and enrollment, participants will complete several baseline measures, and those who have consented to participate in the MRI scanning component will be invited to participate in an optional NIDA sub-study protocol. Participants will begin a 13-week cognitive-behavioral smoking cessation program consisting of weekly meetings with two trained study staff members for weeks 1-7 of study treatment, and meetings every other week thereafter (i.e., weeks 9, 11, and 13). For the revised study protocol up to 60 participants will undergo an MRI scan in week 2 of study treatment, and will be required to abstain from smoking for 24 hours before all MRI scans as verified by breath carbon monoxide measures. An additional 30 individuals who do not meet MRI scanning inclusion criteria (e.g., are left handed), but are otherwise eligible to enroll, will complete only the treatment-related portions of the study. During the third treatment meeting in week 3, participants will be randomized into one of two treatment conditions (i.e., psilocybin or NRT).

After randomization, the 40 participants assigned to the psilocybin condition will receive specialized preparation for their psilocybin session in treatment meetings 3 and 4. They will then

be administered 30mg/70kg psilocybin on their mutually agreed upon Target Quit Date (TQD) in approximately week 5. These participants will meet with study staff the day after their psilocybin session for debriefing, and 25 of these will additionally complete a 4-6 hour post-psilocybin MRI scan that day. Treatment meetings will continue weekly for two weeks after the psilocybin session, and at 2-week intervals from weeks 9 through 13 to discuss the session experience and to provide support for smoking cessation. Participant smoking status will be assessed repeatedly at five meetings after the psilocybin session using self-report and breath carbon monoxide (CO) samples. Smoking status will also be assessed at three follow up meetings approximately 3, 6, and 12 months after the psilocybin session using self-report, breath, and urine samples. All study meetings will have the option to be completed remotely using a secure online format, including remote breath CO monitoring and mailed in or at home urine sample tests, with the exception of physical examination during initial screening, psilocybin sessions, and MRI scan procedures.

After randomization, the 40 participants in the NRT condition (matched for age, sex, IQ, and smoking severity) will receive specialized preparation for NRT administration. In approximately week 5, these participants will begin a standard 8 to 10 week NRT treatment regimen according to recommended label usage (For individuals who smoke more than 10 cigarettes per day: 21mg daily weeks 1-6, 14mg daily weeks 7-8, 7mg daily weeks 9-10. For individuals who smoke 10 or less cigarettes per day: 14mg daily for weeks 1-6, 7mg daily for weeks 7-8; FDA label, PDR.net). Participants in the NRT condition will have brief weekly counseling meetings and provide exhaled CO samples to test for smoking status in weeks 6, 7, 9, 11, and 13. These individuals will also have follow-up meetings at approximately 3, 6, and 12 months post target-quit date to assess smoking status. Participants randomized to the NRT condition who are also completing the MRI portion of the study (n=25) will complete their second MRI scan in week 5, immediately before initiating use of the nicotine patch. For both treatment conditions, MRI participating volunteers who provide biologically verified (i.e., breath CO), and self-report seven-day point prevalence abstinence at 3 months post-TQD will receive a final follow-up MRI scan.

After completing the 6-month follow-up, participants initially randomized to the NRT condition (n=40) will be given the option to undergo a single high-dose (30mg/70kg) psilocybin session. If they choose to take part, they will receive two weekly preparation meetings, one psilocybin session and next-day integration meeting, and two weekly follow-up meetings. For individuals still smoking at that time, the session will serve as an additional quit attempt and provide further data on psilocybin-facilitated smoking cessation. For participants who are no longer smoking, the session will focus on promoting long-term abstinence. The rationale for offering an optional psilocybin session to all participants in the NRT condition is twofold. Firstly, we hope to reduce differential dropout among participants randomized to the NRT condition, and encourage them to complete study treatment through 6-month follow-up. Secondly, in offering this optional session to those who were both successful and unsuccessful in quitting smoking using NRT, we can avoid encouraging poor treatment compliance in the NRT condition (i.e., intentionally failing to quit smoking in order to receive additional treatment with psilocybin). For participants from the NRT condition who choose to take part in the optional psilocybin session, the remaining long-term follow-up meeting will be shifted to take place at 6 months after their psilocybin session (in place of their original 12-month follow-up). For participants from the NRT condition who decline to take part in the optional psilocybin session, study follow-ups will proceed as originally planned at 12 months after their originally assigned Target-Quit Date.

Volunteer Accrual and Screening: Potential participants will learn of the protocol by posted, and internet-posted notices, newspaper advertisements, and word of mouth referral. Volunteers will be screened via a secure online screening questionnaire hosted on a Johns Hopkins approved Qualtrics account and/or telephone to determine whether they meet major inclusion/exclusion criteria, and thus whether they are eligible for an in-person screening session. Individuals will not sign consent before telephone screening. It would not be practical to mail individuals a consent form for the telephone screen because we believe that individuals could find this inconvenient and would not respond, and also that some individuals might be unwilling to provide their mailing address. Individuals who do not pass online / telephone screening or choose not to participate will be asked whether they want us to keep their personal contact information. If the volunteer agrees, this information will be added to an existing approved research database established with application number: BPRU00-01-31-02. Identifiers for those individuals who do not want us to retain their information will be destroyed. The rationale for having the research database is that we want to be able to summarize the reasons that individuals failed screening or choose not to participate in this study, and we want to be able to contact individuals who might qualify for future studies. We anticipate that this protocol could be one of a set of studies with psilocybin and related hallucinogens that would continue for at least the next 5 years. Because the inclusion/exclusion criteria may be changed in the future, it is possible that excluded volunteers may be eligible for studies in the future.

Volunteers who do not fail online / telephone screening will be invited to the BPRU on the Johns Hopkins Bayview Campus for in-person screening. Individuals who complete an online / telephone screening and do not meet the following specific MRI-related study inclusion criteria, will still be eligible to screen and enroll in the treatment portion of the study. This includes individuals who: a) are left-handed; b) may have magnetic/metallic materials in their body; c) suffer from claustrophobia; d) are over 65 years of age; and e) indicate they do not have sufficient free time to complete the MRI related tasks, but would otherwise be available to complete the treatment portion of the study.

Volunteers who fail screening at BPRU but who, in the judgment of the investigators, might be eligible for a future study will be asked to complete IRB-approved HIPAA-IRB Form 3 “Authorization to contact you about future research studies.” Volunteers' files will be kept in a locked room and treated as confidential research material. All data will be collected using the unique identification code numbers assigned to each volunteer upon entry to the study. No individual identifiable information will be released without written authorization. These procedures have been used in the past to protect confidentiality of study data and no instance of loss of confidential information has occurred.

Two hundred volunteers will sign consent to be screened to provide 130 participants who start the study, and 115 participants to participate until completion. Consent will be obtained at BPRU at a scheduled meeting after volunteers have passed telephone screening. Due to COVID-19 space restrictions, participants and study staff may review the consent form remotely or onsite in separate rooms over a secure online platform (e.g., Zoom) to go over study procedures and answer questions before signing consent. Informed consent will be signed at BPRU in-person. Participants will be provided an information sheet about COVID-19 per JH SOM Research Visit Guidelines, as well as information about personal protective equipment (PPE) requirements for their upcoming visit. Participants will be encouraged to socially distance per national and regional guidelines. Written informed consent will be updated to reflect the measures described herein.

Participants will complete a pre-visit COVID-19 symptom questionnaire within 24 hours of all in-person visits, and/or be screened with a symptom questionnaire upon arrival at the

Behavioral Pharmacology Unit (BPRU). The screening questionnaire will assess for possible COVID-19 symptoms including:

- Elevated body temperature
- Presence of cough
- Loss of smell or taste
- Presence of fatigue or general malaise
- Presence of headache
- Presence of body aches
- Adherence to social distancing guidelines
- Known contact with an individual who tested positive for COVID-19 in the past 14 days
- Engagement in high-risk behaviors (e.g. participation in large gatherings without social distancing or use of PPE)

The screening questionnaire may be subject to change based on new information about COVID-19. Body temperature measurement will also occur upon arrival at the BPRU.

Participants will be allowed to take as much time as necessary to decide whether or not to sign consent. Study staff will discuss the consent form with the volunteer after they have read the consent form, as well as providing the recruitment materials for the optional NIDA sub-study protocol to be reviewed at the time of enrollment for individuals who are eligible for MRI scanning. Study staff will ask participants to read a paragraph from the consent form out loud to assess English literacy and will ask questions regarding basic study procedures to assess the individual's understanding of the consent forms. Participants may take the consent forms home to review and return to sign consent if they wish. Non-English speakers and those with language or hearing impairments will not participate in the study. Participants will be physically and psychologically healthy adult volunteers (approximately equal numbers of males and females). Participants completing the MRI scanning portion of the protocol at NIDA must be between the ages of 21 and 65 years old. For those who will not be completing MRI scanning at NIDA (treatment only), volunteers ages 21 to 80 may participate. We expect to locate participants who are both qualified and motivated to adhere to the rigorous conditions of the study. Participants may or may not have used hallucinogens in the past. Our ongoing trial, as well as considerable data from prior studies, documents the safety of administering psilocybin at the proposed dose level to participants without histories of hallucinogen use. However, to attenuate the possibility of non-optimal response in individuals with higher body weight, this study will use an absolute maximum dosage of 50 mg, which is approximately equivalent to absolute doses we have administered safely to heavier participants in previous studies.

Potential participants will be carefully screened to eliminate those with significant medical or psychiatric illnesses. Evaluation will include a history and physical examination, CBC, SMA-24 (or equivalent), ECG, and urine drug test. Participants will also be requested to refrain from illicit drug use during the course of the study and a urine test will be conducted before the administration of psilocybin. Left-handed individuals are ineligible for MRI scanning, but may otherwise complete the study treatment. Pregnant or nursing women are ineligible; female participants will receive a urine pregnancy test at intake and before psilocybin administration, and must use effective methods of contraception during the study. Other medical exclusion criteria are presented in section 5. With regard to psychiatric screening, based on clinical interviews and a structured psychiatric diagnostic interview, participants will be eligible if they are judged to be psychologically stable and do not meet any of the psychiatric exclusion

criteria (as defined by DSM-IV) presented in section 5. Psychiatric screening interviews may take place remotely via secure online platform.

All standard study meetings, meeting content, and duration are summarized here:

**Timeline** [a]	**Psilocybin condition meetings (duration)**	**NRT condition meetings (duration)**
	Screening Day 1/ Consent (6 hrs)	
	Screening Day 2 (2-3 hrs)	
	Optional NIDA sub-study protocol / MRI Scan 1 (6-8 hrs)*	
Week 1	**MRI task training at NIDA (2-3 hrs)***<br>**Treatment meeting 1 (1-2 hrs)**<br>Study orientation<br>Assign Target Quit Date (TQD)<br>Sign contract to quit / smoking diary introduction	
Week 2	**MRI scan 2 (4-5 hrs)***<br>**Treatment meeting 2 (1-2 hrs)**<br>Carbon monoxide demonstration<br>Smoking financial costs<br>Identifying motivation to quit	
Week 3	**Treatment meeting 3 (90 min)**<br>*Randomization to treatment condition*<br>Discuss previous quit attempts<br>Weight gain and quitting smoking<br>Recovery symptoms<br>Discussing psilocybin effects	**Treatment meeting 3 (90 min)**<br>*Randomization to treatment condition*<br>Discuss previous quit attempts<br>Weight gain and quitting smoking<br>Recovery symptoms<br>Discussing NRT effects
Week 4	**Treatment meeting 4 (90 min)**<br>Smoking diary discussion<br>Dealing with urges after TQD<br>Preparing to quit<br>What to expect in psilocybin session	**Treatment meeting 4 (90 min)**<br>Smoking diary discussion<br>Dealing with urges after TQD<br>Preparing to quit<br>How to take NRT
Week 5	**Psilocybin session / TQD (8 hrs)**<br>**MRI scan 3 (4-6 hrs; Next day)***<br>**Treatment meeting 5 (1-2 hrs; Next day)**<br>Participant Narrative of Session	**MRI scan 3 / TQD (4-6 hrs)***<br>**Treatment meeting 5 (1-2 hrs)**<br>Initiation of NRT
Week 6	**Treatment meeting 6 (45 min)**<br>Identifying triggers<br>Overcoming urges to smoke	**Treatment meeting 6 / TQD (45 min)**<br>Identifying triggers<br>Overcoming urges to smoke
Week 7	**Treatment meeting 7 (45 min)**<br>Supporting smoking abstinence	**Treatment meeting 7 (45 min)**<br>Supporting smoking abstinence
Week 9	**Treatment meeting 8 (45 min)**<br>Supporting smoking abstinence	**Treatment meeting 8 (45 min)**<br>Supporting smoking abstinence
Week 11	**Treatment meeting 9 (45 min)**<br>Supporting smoking abstinence	**Treatment meeting 9 (45 min)**<br>Supporting smoking abstinence
Week 13	**Treatment meeting 10 (45 min)**<br>Clinical interview	**Treatment meeting 10 (45 min)**<br>Clinical interview
…3 months post-TQD	**Follow-up meeting (2-4 hrs)**<br>**MRI Scan 4** [b] **(4-6 hours)***	**Follow-up meeting (2-4 hrs)**<br>**MRI Scan 4** [b] **(4-6 hours)***
…6 months post-TQD	**Follow-up meeting (2-4 hrs)**	**Follow-up meeting (2-4 hrs)**
…12 months post-TQD	**Follow-up meeting (2-4 hrs)**	**Follow-up meeting (2-4 hrs)**

* Indicates MRI procedures to be completed only by participants who are eligible for MRI scanning (n=60).
[a] Times listed in the table are approximate.
[b] MRI Scan 4 only for participants showing biologically verified abstinence at 3-month follow-up.

<u>**Meetings Before and After TQD (Psilocybin Condition):**</u> Before the psilocybin session day participants in the psilocybin condition will meet with one or more of the study staff on at least 3 occasions, for a minimum of 4 hours of contact time before the session day. Some flexibility in scheduling will be allowed to account for unexpected absences such as travel commitments or illnesses. The main purpose of the participant-staff meetings is to develop rapport and trust,

which we believe helps minimize the risk of fear or anxiety reactions during the psilocybin session. Additional meetings and contact hours (up to 10 hours) will be scheduled if it is judged necessary to establish rapport and trust. The participant's life history and current situation in life will be reviewed. Intentions and expectations for the psilocybin session will be discussed. Furthermore, a structured cognitive-behavioral smoking cessation intervention will be implemented throughout the course of these meetings to prepare participants for smoking abstinence and potential withdrawal. Throughout the preparatory meetings, participants will be encouraged to view the psilocybin session as providing an opportunity for deep reflection about the role of smoking in their lives. The purpose of the psilocybin session is to enhance motivation for initiating a quit attempt. Procedures for drug administration and the conduct of the session will be similar to procedures used in our previous (Griffiths et al., 2006, 2008, 2011) and ongoing studies with psilocybin (IRB # NA_00001390 in cancer patients; IRB # NA_00020767 in healthy adults), and are described below in the section "Conduct of Psilocybin Sessions."

Participants will meet with study staff for debriefing one or two days after the psilocybin session. Participants will then meet weekly with study staff for 2 weeks after their psilocybin session (i.e., in weeks 6 and 7), and at 2-week intervals until the end of the 13-week study treatment (i.e., in weeks 9, 11, and 13). The general purpose of the meetings following the session is to provide a context for discussing and integrating experiences from the psilocybin session, and to provide support for smoking cessation. Additional contact hours will be scheduled if it is judged that the participant would benefit from further meetings with study staff to discuss experiences from the session or provide additional support for smoking abstinence. Aside from the psilocybin session and MRI scan visits (if applicable), all meetings may be held remotely using a secure online platform, including use of remote CO monitoring and mailed in or at home urine sample testing.

Meetings Before and After TQD (NRT Condition): Before the target-quit date participants in the NRT condition will meet with one or more of the study staff on at least 3 occasions, for a minimum of 4 hours of contact time before drug administration. Some flexibility in scheduling will be allowed to account for unexpected absences such as travel commitments or illnesses. The main purpose of the participant-staff meetings is to prepare participants for NRT transdermal patch administration, and to deliver a cognitive-behavioral smoking cessation intervention identical to that received by participants in the psilocybin condition. During these meetings study staff will answer any questions related to NRT, examine motivations to quit smoking, and encourage preparation for smoking cessation on the target-quit date. Participants in this condition will be provided with NRT patches and instructed how to use the patches, which may be mailed to participants to reduce unnecessary in-person visits. In week 5, participants in the NRT condition will begin a standard 8 to 10 week course of NRT for smoking cessation. For MRI participating volunteers, nicotine patches will be initiated directly after their week 5 MRI scan. All participants in the NRT condition will have five additional counseling meetings with study staff in weeks 6, 7, 9, 11, and 13, where they will provide self-report and breath samples to assess smoking status and complete study questionnaires. The study physician will be available during the study to discuss any potential side effects or medical issues. Participants will complete questionnaires and provide breath and urine samples to evaluate smoking status at approximately 3, 6, and 12 months post-TQD.

Optional Meetings / Psilocybin Session (NRT Condition): After completing the 6-month follow-up, participants initially randomized to the NRT condition will be eligible to undergo an optional high-dose (30mg/70kg) psilocybin session. If they choose to take part, they will receive

two weekly preparation meetings, one high-dose psilocybin session and next-day integration meeting, and two weekly follow-up meetings. For individuals unsuccessful in their smoking cessation attempt using NRT, this session will serve as an additional quit attempt and provide further smoking cessation efficacy data on psilocybin pharmacotherapy in treatment-resistant populations. For participants who successfully quit smoking using NRT, this session will focus on promoting long-term abstinence. For participants from the NRT condition who choose to take part in the optional psilocybin session, their remaining follow-up meeting will be shifted to take place at 6 months after their optional psilocybin session (in place of their original 12-month follow-up). Participants who choose not to take part in this process will not be penalized, and will be asked to complete the study as originally planned through 12-month follow-up.

All optional study meetings, meeting content, and duration are summarized here (for participants in NRT condition only):

**Timeline**	**Optional Meetings / Psilocybin Session**
Week 1B (after 6-month follow-up)	**Psilocybin prep. Meeting 1 (90 min)**<br>Discuss previous quit attempt<br>Discuss psilocybin effects
Week 2B	**Psilocybin prep. Meeting 2 (90 min)**<br>What to expect in psilocybin session
Week 3B	**Psilocybin session (8 hrs)**<br>**Integration meeting 1 (45 min; Next day)**<br>Participant Narrative of Session
Week 4B	**Integration meeting 2 (45 min)**<br>Supporting smoking abstinence
Week 5B	**Integration meeting 3 (45 min)**<br>Supporting smoking abstinence
**…6 months post-psilocybin**	Follow-up meeting (2-4 hrs)

MRI Brain Scanning Sessions: For the revised study protocol, up to 60 participants in the psilocybin and NRT conditions (n=30 per group) will undergo between two and four MRI brain scanning sessions (4-6 hours each), taking place at the NIDA intramural research program (IRP) on the Bayview campus under the supervision of Dr. Amy C. Janes. Dr. Amy C. Janes is Chief of the Neuroimaging Research Branch at the National Institute on Drug Abuse-Intramural Research Program (NIDA-IRP) and will serve as the Principal Investigator of the NIDA-IRP protocol addendum that covers all neuroimaging procedures performed in this study. Only participants demonstrating biologically verified seven-day point-prevalence smoking abstinence at the initial 3-month follow-up will be asked to undergo a final MRI scan at that time. All MRI participating volunteers will be financially compensated for their time on MRI scanning days with funding provided by NIDA IRP. Before MRI scanning, participants will provide a urine sample for a drug urine screen. MRI scanning will not proceed unless the drug screen results are negative, or unless participants pass a neuromotor evaluation showing they are not currently intoxicated, and are cleared to proceed by the NIDA study physician. NIDA IRP has resumed in-person research, and instituted COVID-19 safety measures consistent with those described in the current protocol (i.e., physical distancing, PPE and mask use during all visits, pre-visit symptom checks, thorough equipment cleaning between participants), which will be used for participant and personnel safety at in-person MRI visits. Additionally, NIDA IRP will be providing point-of-care COVID-19 testing for participants at in-person study visits. Only individuals with negative COVID-19 tests will be allowed to proceed with their scheduled visit. Otherwise they will be advised to quarantine according to CDC guidelines and required to provide negative COVID-19 test results before resuming in-person study visits as described below.

The first (optional) MRI scan may occur as part of a voluntary NIDA approved sub-study protocol, which is a large-scale (*N*=2500) investigation of genetic polymorphisms associated with substance dependence. If participants agree to participate in this sub-study, the related MRI scan will be completed after study-screening, and will produce an MRI scan of clinical quality to be examined for incidental findings by a Johns Hopkins Hospital (JHH) neuroradiologist. Participants who decline to complete the NIDA sub-study will receive a clinical MRI scan at their first MRI scanning visit to be examined for incidental findings by a JHH neuroradiologist. The next (required) MRI scan will take place in approximately week 2 of treatment after an initial orientation meeting, and before randomization in week 3. The following (required) MRI scan will occur in approximately week 5 of treatment. For the psilocybin condition this scan will occur one day after psilocybin administration. For the NRT condition this scan will occur immediately before initiating NRT administration. A final, conditional MRI scan will take place at 3-month follow-up only for participants demonstrating biologically verified seven-day point-prevalence smoking abstinence at that time. After completing the 6-month follow-up all participants initially randomized to the NRT condition will be eligible to undergo additional (optional) meetings including a high-dose psilocybin session. However, no further MRI scanning will be conducted after the 3-month time point.

Participants will be asked to abstain from smoking for 24 hours before all MRI scanning sessions, as verified by measures of exhaled breath CO. MRI scans will last roughly 4 to 6 hours, and be conducted at the NIDA IRP building adjacent to the BPRU by a trained MRI operator. A trained study staff member will be present during all MRI scanning sessions. Any incidental findings will be communicated by the JHH neuroradiologist to the NIDA IRP Medical Advisory Investigator, who will transmit incidental findings to Dr. Umbricht, the Hopkins study team physician. Dr. Umbricht will report any incidental findings to participants at an in-person meeting, over the phone, or via mail as deemed appropriate commensurate with the significance of the findings.

Monitor Telephone Contact: In order to provide encouragement for smoking abstinence, and to monitor participant status after psilocybin or NRT administration, participants will receive a brief (<5 min.) telephone call or inquiry via text message from study staff every day (with the exception of days on which a monitor meeting is scheduled) for the first seven days after the TQD. An occasional day may be missed for unavoidable reasons, but efforts will be made to minimize this. Telephone discussions may continue up to 4 weeks after TQD on a less frequent basis (e.g., 3 times per week) if the study team judges that continuing daily support would be helpful. For all participants, the primary study staff member will be available 24 hours for telephone consultation, and in the case of side effects or medical issues, Dr. Annie Umbricht will be available by phone.

Cognitive-Behavioral Therapy for Smoking Cessation: Starting with the first treatment meeting participants will be provided guidance and instructions on a variety of self-help cognitive-behavioral therapy (CBT) techniques shown to be effective in smoking cessation in controlled studies (Perkins et al., 2008; Sykes & Marks, 2001; Marks, 2005). These are easily understood techniques designed to raise the awareness of the individual about the motivations, antecedents, and consequences of smoking. Examples of these techniques include placing a rubber band around one’s pack of cigarettes to serve as a cue to reduce the automatic and habitual nature of smoking, the use of a score card to record when each cigarette is smoked, and the use of easily remembered acronyms to help highlight the unpleasant aspects of smoking each time a cigarette is smoked (e.g., that the cigarette is making the person feel rotten). This program

fits nicely with the proposed design, in that it includes procedures to implement both before a target-quit date, and other procedures to implement after a target-quit date. Study staff will continue to discuss these techniques with the participant during staff-participant meetings throughout the study. For a summary of CBT modules, see the study timeline table in the study design section above.

Conduct of Psilocybin Session: Participants in the psilocybin condition, as well as those in the NRT condition who elect to undergo an optional psilocybin session after the 6-month follow-up, will be instructed to consume a low-fat breakfast before reporting to the laboratory for the psilocybin session. Participants must refrain from smoking during the time of drug action on the psilocybin session day. On the day of the psilocybin session, the last cigarette may be smoked 1 hour before psilocybin administration. Participants will be instructed to abstain from smoking and drinking alcohol for at least the next 24 hours until the post-psilocybin MRI scan has been conducted (when applicable).

Before drug administration, participants will provide a urine sample for a drug urine screen and, for females, for pregnancy testing. Sessions will not proceed unless drug tests are negative for illicit drug use, and pregnancy tests are negative. Psilocybin capsules will be administered with approximately 100 mL water. The study staff, under the supervision of the investigators, will be present in the room and available to respond to participants' physical and emotional needs during the full course of the session (at least 7 hours).

These sessions often require session facilitator(s) and the participant to be in a session room together for an extended period of time and may require periods of physical contact (e.g. for adjusting blood pressure cuff, therapeutic touch etc.). On session days, it is possible that participants may inadvertently remove PPE while under the influence of psilocybin. We will adhere to the following additional safety measures for these sessions:

- COVID-19 testing will be available for participants and facilitators prior to these meetings if there is special concern raised about the possibility of exposure during the final preparation or drug administration day. The availability of testing will be discussed during preparation.

- COVID-19 testing will be performed within 72 hours of each psilocybin session with a negative result, or written proof of such testing with negative result within 72 hours of each psilocybin session must be presented if the participant claims to have been tested. This will be required to move forward with a psilocybin administration session. Participants can alternatively bring proof of full COVID-19 vaccination being completed at least 2 weeks prior to their psilocybin session in lieu of COVID-19 testing. Vaccination status will be verified via official written documentation (e.g., CDC card or healthcare provider records) and copies will be kept on file. Study team members overseeing psilocybin sessions have presently been fully vaccinated.

- Participants will be asked to maintain social distancing procedures and use PPE in public places between the baseline visit and the day after the drug administration session.

- Session facilitators will wear a surgical mask at all times during these two visits. Facilitators will wear a face shield when there is less than 6 feet of space between any two individuals. Face shields may be omitted in cases where participants and staff have been vaccinated for COVID-19.

- Session facilitators will wear gloves when touching the participant (adjusting blood pressure cuff, helping the participant to the bathroom, etc.). Gloves may be omitted in cases where participants and staff have been vaccinated for COVID-19.

- The study team will monitor the data and guidelines on duration of vaccine effectiveness and correlate this to when the vaccine was administered to any given subject when potentially modifying COVID-19 related study safety procedures.

- A washable couch cover will be used and cleaned between participants.

- Headphones will be sanitized between participants, or disposable headphone covers will be used.

During the session, participants will lie on a couch, wear eyeshades, and listen to a program of music through headphones. The participant will be encouraged to focus her or his attention inward. The eyeshades and music are intended to encourage this inward reflection. Heart rate and blood pressure will be measured pre drug administration, at 30, 60, 90, 120 minutes after drug administration, and then hourly until at least 6 hours after drug administration and until drug effects have subsided. Sessions are expected to last 7 to 10 hours. Baseline heart rate and blood pressure will be obtained after the participant has been recumbent for 5 minutes. Around the time of each physiological measurement, monitors will use questionnaires to rate the presence and intensity of behaviors, signs, and reported symptoms, including sleepiness, amount of speech, anxiety, stimulation/arousal, tearing/crying, nausea/vomiting, yawning, restlessness, feelings of unreality, visual changes, euphoria, and peacefulness. Video and audio recordings will be made throughout the session. At the end of the experimental session, participants will complete pencil-and-paper or computer-based instruments designed to assess acute subjective experiences associated with the psilocybin session (see section 7), and will be asked to write a narrative description of the experience of the psilocybin session that evening. On the first meeting with the monitor after the session (1 or 2 days after each session), the study staff will complete questionnaires retrospectively rating various behaviors and experiences observed on the session day and reported by the volunteer during the meeting (e.g. Next-Day Monitor rating).

NRT Condition: Participants in the NRT condition will receive preparation and instructions from study staff on how to use the nicotine transdermal patch, which may be mailed to them to reduce in-person contact. Participants will receive detailed information on NRT and potential side effects prior to patch administration. On the day after their Target-Quit Date in week 5, they will be provided with a 2-week supply of medication, and instructed to begin a standard 8 to 10 week transdermal nicotine patch regimen according to recommended label usage (For individuals who smoke more than 10 cigarettes per day: 21mg daily for weeks 1-6, 14mg daily for weeks 7-8, 7mg daily for weeks 9-10. For individuals who smoke 10 or less cigarettes per day: 14mg daily for weeks 1-6, 7mg daily for weeks 7-8; FDA label, PDR.net). For MRI participating volunteers receiving NRT (n=25), the Target-Quit Date will occur 24 hours prior to the MRI scanning day in week 5, and participants will begin using NRT immediately after that MRI scan is completed. To ensure matching treatment procedures, participants randomized to the NRT condition who are not MRI participating volunteers, and are completing only the treatment portion of the protocol (n=15), will also stop smoking 24 hours prior to their meeting in week 5 (to be confirmed by breath CO). At this meeting they will receive their first 2-week supply of transdermal nicotine patches and initiate NRT use. Participants will receive additional supplies

of appropriate dose patches at 2-week intervals around their regularly scheduled treatment meetings. Participants will be asked to notify study staff immediately in case of adverse events or side effects, and will determine with the study physician whether NRT dosage should be adjusted, or permanently discontinued. In the unlikely event of a severe side effect such as a myocardial infarction/arrhythmia, participants will be advised to immediately discontinue NRT use. Under these circumstances, participants will be asked to continue the study protocol meetings to completion.

After completing the 6-month follow-up, participants initially randomized to the NRT condition will be eligible to undergo an optional high-dose (30mg/70kg) psilocybin session. If they choose to take part, they will receive two weekly preparation meetings, one high-dose psilocybin session and next-day integration meeting, and two weekly follow-up meetings. For individuals who were unsuccessful in their smoking cessation attempt using NRT, this session will serve as an additional quit attempt and provide further smoking cessation efficacy data on psilocybin pharmacotherapy in treatment-resistant populations. For participants who successfully quit smoking using NRT, this session will focus on promoting long-term abstinence. For participants from the NRT condition who choose to take part in the optional psilocybin session, their remaining follow-up meeting will be shifted to take place at 6 months after their optional psilocybin session (in place of their original 12-month follow-up). Data collection at optional meetings, psilocybin session, and follow-ups will be identical to those in the psilocybin condition, including measures of breath CO and questionnaire assessments of smoking abstinence self-efficacy, craving, withdrawal, and subjective effects of psilocybin. Participants who choose not to take part in this process will not be penalized, and will be asked to complete the study as originally planned, by attending the remaining 12-month follow-up meeting.

Baseline and Follow-up Data Collection: Baseline psychological data will be obtained on each volunteer at the time of enrollment. Smoking history will be collected, and several smoking related questionnaires will be administered. Psychiatric history (including family history), psychoactive drug-use history (including alcohol) and information about employment status and current functioning (including mood and psychological and psychosomatic symptoms) will be obtained using standard psychiatric instruments such as the SCID and the BSI. In addition, baseline assessments will be taken on a number of psychological variables that may be impacted by a psilocybin experience, e.g. psychiatric symptoms and mystical-type experience. Re-assessment of several measures will be made throughout the study and at follow up visits. For MRI participating volunteers (N=60), functional neuroimaging (fMRI) data will also be collected in approximately weeks 2 and 5 weeks of treatment, and again at 3-month follow-up for participants demonstrating biologically verified 7 day point-prevalence smoking abstinence at that time point. A thorough description of measures and the time of their assessment is included in section 7.

Assessment of Smoking Cessation: Participants will complete smoking assessments (including biological verification and other measures as shown in section 7) at 1 to 2 week intervals throughout the course of the study. These assessments will include biological verification of smoking status with breath carbon monoxide during the 13-week treatment period. Additionally, urine cotinine samples will be collected at screening, and at 3, 6, and 12-month follow-ups. Participants will have meetings in weeks 6, 7, 9, 11, and 13 after their initial TQD to provide self-report data on smoking behavior, exhaled breath samples, and to complete questionnaires. All participants will have follow-up meetings at 3, 6, and 12 months post-TQD, or at 3 and 6 months post-TQD, and 6 months post-psilocybin administration for participants in the NRT

condition who choose to undergo an optional psilocybin session. Breath samples may be taken remotely using an individually assigned mobile CO breath monitor device, and urine samples may be provided remotely by mail or tested at home.

b. Study duration and number of study visits required of research participants.

The pre-treatment phase of the study, including screening, enrollment, and sub-study protocol completion will require at least two full days (approx. 4-6 hours each) of meetings. Because of the large number of screening measures, screening and intake for either group may require 2 appointments. The treatment phase of the study, including treatment meetings, psilocybin session (when applicable), and MRI scans (when applicable) will require 11 meetings over approximately 13 weeks. For the post-treatment follow-up phase of the study, participants will have meetings at approximately 3, 6, and 12 months post-target quit date for follow-up assessment (2-4 hours each). Additionally, participants from the NRT group who choose to partake in an optional psilocybin session will undergo six supplementary meetings, including two weekly preparatory meetings, one high-dose psilocybin session and next-day integration meeting, and two weekly follow-up meetings to assess psychological and smoking status. However, participation in these optional meetings will not substantially affect overall study duration. Thus, the study will comprise a total of approximately 16-22 study meetings over the course of roughly 14 months. Participants who are not participating in MRI scanning (n=40) will have the same number of study visits as those who undergo NIDA MRI scanning (n=60), though study visits in weeks 1, 2, 5, and potentially 3-month follow-up will be approximately 4-5 hours shorter in duration.

c. Blinding, including justification for blinding or not blinding the trial, if applicable.

Because of the distinctive nature of the two treatment protocols, and the different preparation required for each (e.g., single day-long psilocybin session vs. 8 to 10 week daily nicotine transdermal patch use), blinding will not be utilized.

d. Justification of why participants will not receive routine care or will have current therapy stopped.

Participants will have multiple previous smoking cessation attempts, which may or may not include pharmacological (e.g., nicotine replacement, bupropion) or nonpharmacological therapies. Thus, participants will be those for whom routine care has proven unsuccessful to date. Participants who fail screening or decide not to participate after screening will be directed to American Lung Association and U.S. Department of Health and Human Services sponsored websites (http://www.lung.org/stop-smoking/; http://betobaccofree.hhs.gov/quit-now/) that provide information designed to help individuals quit smoking. Participants can discontinue participation in the study at any time. In the case of adverse events or side effects, participants will be instructed to contact their primary study staff member immediately. Adverse events will be documented, and participants will consult with the study physician before continuing with the study. Participants in the NRT condition reporting a severe side effect such as a myocardial infarction/arrhythmia will be advised to immediately discontinue NRT. However, in such cases, we will request that participants discontinuing NRT complete the study protocol meetings.

e. Justification for inclusion of a placebo or non-treatment group.

This is a proof-of-concept pilot study assessing the feasibility of the proposed intervention. Therefore, no placebo or non-treatment group is included.

f. Definition of treatment failure or participant removal criteria.

Treatment failure is defined by the inability to achieve 7-day point-prevalence abstinence at 6-month follow-up, as confirmed by urine cotinine, breath carbon monoxide, and self-report data. However, all participants will be evaluated throughout the initial 12-month follow up regardless if they have quit smoking. Participants will be removed from the study for non-compliance with study requirements.

g. Description of what happens to participants receiving therapy when study ends or if a participant's participation in the study ends prematurely.

Participants who fail screening or decide not to participate after screening will be directed to American Lung Association and U.S. Department of Health and Human Services sponsored websites (http://www.lung.org/stop-smoking/; http://betobaccofree.hhs.gov/quit-now/) that provide information designed to help individuals quit smoking. Participants who voluntarily discontinue the study protocol prior to drug administration will be directed to the same websites providing information on quitting smoking. Participants who experience adverse effects due to NRT will be asked to continue the study protocol meetings to completion, and will determine with the study physician on a case-by-case basis whether NRT should be permanently discontinued, or if a change in daily dosage may be appropriate. In the event of a severe side effect such as a myocardial infarction/arrhythmia participants will be advised to immediately discontinue NRT administration. In such cases, smoking cessation counseling meetings with study staff would continue as scheduled, and participants will continue to be encouraged to abstain from smoking without pharmacotherapy.

5. Inclusion/Exclusion Criteria

Inclusion criteria:

- 21 to 80 years old
- Have given written informed consent
- Read, write, and speak English
- Be a daily smoker with multiple unsuccessful previous quit attempts, and report a continued desire to quit smoking
- Agree to abstain from smoking for the psilocybin session from 1 hour before psilocybin administration until at least 30 hours afterwards
- Agree to consume approximately the same amount of caffeine-containing beverage (e.g., coffee, tea) that he/she consumes on a usual morning, before arriving at the research unit on the morning of drug session day. If the volunteer does not routinely consume caffeinated beverages, he or she must agree not to do so on session day
- Agree to refrain from using any psychoactive drugs, including alcoholic beverages, within 24 hours of psilocybin administration. Exceptions include caffeine and nicotine

- Be healthy as determined by screening for medical problems via a personal interview, a medical questionnaire, a physical examination, an electrocardiogram (ECG), and routine medical blood and urinalysis laboratory tests
- Agree to abstain from smoking for 24-hours prior to study visits in week 2 and 5

<u>Inclusion criteria for MRI scanning eligibility only:</u>

- 21 to 65 years old
- Agree to abstain from smoking and alcohol for 24-hours, and caffeine for 12-hours prior to MRI scanning*
- Be right-handed*

<u>General medical exclusion criteria:</u>

- Women who are pregnant (positive pregnancy test) or nursing, or are not practicing an effective means of birth control
- Cardiovascular conditions: uncontrolled hypertension with resting blood pressure systolic >150 or diastolic >95, angina, a clinically significant ECG abnormality (e.g., atrial fibrilation), TIA in the last 6 months stroke, peripheral or pulmonary vascular disease
- Epilepsy with history of seizures
- Insulin-dependent diabetes; if taking oral hypoglycemic agent, then no history of hypoglycemia
- Currently taking psychoactive prescription medication on a regular basis
- Currently taking on a regular (e.g., daily) basis any medications having a primary centrally-acting pharmacological effect on serotonin neurons or medications that are MAO inhibitors. For individuals who have intermittent or PRN use of such medications, psilocybin sessions will not be conducted until at least 5 half-lives of the agent have elapsed after the last dose.
- Have HIV or Syphilis.
- Have any current neurological illnesses including, but not limited to, seizure disorders, frequent migraines or on prophylaxis, multiple sclerosis, movement disorders, history of significant head trauma, or CNS tumor.
- Morbidly obese, or severely underweight as determined by medical examination.
- Recent (within past 12 months) or extensive history of hallucinogen use (>20 lifetime uses).

<u>General medical exclusion criteria for MRI scanning eligibility only:</u>

- Not suitable to undergo an MRI session due to certain implanted devices (cardiac pacemaker or neurostimulator, some artificial joints, metal pins, surgical clips or other implanted metal parts), body morphology, or claustrophobia.

<u>Psychiatric Exclusion Criteria:</u>

- Current or past history of meeting DSM-IV criteria for Schizophrenia, Psychotic Disorder (unless substance-induced or due to a medical condition), or Bipolar I or II Disorder.
- Current or past history within the last 5 years of meeting DSM-IV criteria for alcohol or drug dependence (excluding caffeine and nicotine) or severe major depression.
- Have a first or second degree relative with schizophrenia, psychotic disorder (unless substance induced or due to a medical condition), or bipolar I or II disorder.
- Currently meets DSM-IV criteria for Dissociative Disorder, Anorexia Nervosa, Bulimia Nervosa, Major Depression, or Post-traumatic Stress Disorder.

Individuals who do not pass screening or choose not to participate will be asked whether they want us to keep their personal contact information using HIPAA-IRB Form 3. Identifiers for those individuals who do not want us to retain their information will be destroyed. We will retain information because we want to be able to summarize the reasons that individuals failed screening or choose not to participate in this study. Also, because the inclusion/exclusion criteria may be changed in our future studies with psilocybin and related compounds, it is possible that excluded volunteers may be eligible for studies in the future.

6. Drugs/ Substances/ Devices

a. The rationale for choosing the drug and dose or for choosing the device to be used.

Psilocybin dose: On the drug session day, participants will be administered 30mg/70kg psilocybin. Two previous studies in our laboratory on psilocybin suggest that this dose is associated with mystical-type experiences, with some variability across participants (Griffiths et al., 2006; Griffiths et al., 2011). This dose has been safely administered in our laboratory (Griffiths et al., 2006; Griffiths et al., 2011), is in the range of doses examined extensively in previous studies with hallucinogen-naïve individuals (Malitz et al. 1960 Metzner et al. 1965; Pahnke 1963, 1969; Pahnke et al. 1966; Leuner 1981), and is less than that (49mg/70kg) administered safely under a previously authorized IND (Strassman 1992, 1998). Furthermore, to attenuate the possibility of a non-optimal response in individuals with higher body weight, this study will use an absolute maximum dosage of 50 mg, which is approximately equivalent to absolute doses we have administered safely to heavier participants in previous studies with psilocybin.

Transdermal nicotine patch dose: Nicotine replacement therapy (NRT) is widely considered a first-line smoking cessation treatment option that is available over the counter (Silagy et al., 2000; Stead et al., 2012). As such, it was chosen to provide a proven control treatment with known efficacy. NRT patch doses will be administered according to recommended label usage. For individuals who smoke more than 10 cigarettes per day: 21mg daily for weeks 1-6, 14mg daily for weeks 7-8, 7mg daily for weeks 9-10; for individuals who smoke 10 or less cigarettes per day: 14mg daily for weeks 1-6, 7mg daily for weeks 7-8 (FDA label, PDR.net). Patch use will begin in week 5 of treatment, after 24-hours of smoking abstinence.

b. Justification and safety information if FDA approved drugs will be administered for non-FDA approved indications or if doses or routes of administration or participant populations are changed.

N/A; The transdermal nicotine patch is an FDA approved smoking cessation treatment.

c. Justification and safety information if non-FDA approved drugs without an IND will be administered.

N/A – We currently hold an IND for psilocybin and have amended the IND to include the present protocol.

7. Study Statistics

a. Primary outcome variable.

The primary outcome variable will be between group differences of biologically verified (i.e., CO, cotinine) and self-reported smoking abstinence 6 months post-TQD, with a grace period for initiation of abstinence within 14 days of the TQD. This outcome meets success criteria used for FDA evaluation of smoking cessation medications.

b. Secondary outcome variables. Descriptions of Outcome Measures

Measure completed on a daily basis by participant at home (in the evening or at the same time each day, allowing 24 hours between completing measures):

Smoking Diary: Participants will be asked to keep a smoking diary, in which they will record every cigarette smoked, and make a brief note about feelings, antecedents, or consequences of smoking. In the evening or at the same time each day (allowing 24 hours between each entry), participants will review their daily smoking diary, and write a brief description of thought or feelings surrounding their smoking behavior on that day.

Longitudinal measures (Smoking related):

***Carbon monoxide breath and cotinine urine samples as biological measures of smoking*:** These two measures are widely used in smoking cessation studies and provide advantages that complement each other. The carbon monoxide measure, assessed using expired air samples (e.g., Bedfont Smokerlyzer, Bedfont iCO mobile smokerlyzer) can detect very recent smoking and provides immediate results to study staff, but cannot detect smoking that occurred over 24 hours ago. The cotinine assay is collected with urine samples and will not provide results for several days because samples must be sent to an outside laboratory (e.g., Friends Research Institute). However, cotinine assays provide a larger time window of detection, with the ability to detect smoking that has occurred during approximately the last 6 days (SRNT Subcommittee on Biological Verification, 2002). Breath samples may be taken remotely using an individually assigned mobile CO breath monitor, and urine samples may be provided remotely by mail or tested at home.

***Fagerström Test for Nicotine Dependence* (FTND):** This 6-item questionnaire is widely used to characterize the level of dependence of cigarette smokers (Heatherton, Kozlowski, Frecker, & Fagerström, 1991).

***Smoking History Questionnaire* (SHQ):** This questionnaire permits collecting smoking data such as age of smoking initiation, parent and peer smoking behavior, history of quit attempts, and current level of smoking (DiClemente et al., 1991).

***Smoking Abstinence Self-Efficacy* (SASE):** This questionnaire provides a measure of a smoker's confidence in his or her ability to not smoke in 20 challenging hypothetical situations (DiClemente et al., 1985).

***The Wisconsin Smoking Withdrawal Scale* (WSWS):** The WSWS is a 28-item questionnaire designed to assess withdrawal DSM-IV symptoms and other signs of withdrawal (Welsch et al., 1999), and provides 7 construct subscales (anger, anxiety, sadness, concentration, craving, sleep, and hunger). The WSWS subscales have shown good reliability and have been validated in smoking abstinence and nicotine replacement therapy studies (Shiffman et al., 2004).

***Questionnaire on Smoking Urges* (QSU):** This 32-item questionnaire of smoking craving provides two dimensions (anticipation of pleasure from smoking, and anticipation of relief from negative affect and withdrawal), and has shown sensitivity to smoking cessation (Tiffany & Drobes, 1991).

Smoking Cessation Contemplation Ladder: This single item measure is designed to assess an individual's readiness to consider smoking cessation (Biener & Abrams, 1991). Participants are asked to rate their readiness to consider quitting smoking on a scale from 1 (i.e., no thought of quitting), to 10 (i.e., taking action to quit).

Other measures:

***General Self-Efficacy Scale* (GSES):** This is a validated 17-item questionnaire which assesses general self-efficacy (Sherer et al., 1982).

***Brief Symptom Inventory* (BSI):** This questionnaire is a well-validated measure of acute psychological distress (Derogatis, 1993).

***Positive and Negative Affect Schedule Expanded Form* (PANAS-X):** This is a well-validated questionnaire assessing positive and negative affect as two general factors (Watson and Clark 1994, 1997).

***Beck Depression Inventory* (BDI):** The BDI is a well-validated 21-item self-report measure of depressive mood and symptoms (Beck et al., 1961).

***State-trait Anxiety Inventory* (STAI):** The STAI (Form Y; Spielberger, 1983) is a widely used self-report measure of state (temporary) and trait (recurring) anxiety consisting of 40 items assessing apprehension, tension, worry, and nervousness.

***Connectedness to Nature Scale* (CNS):** The CNS is a 14-item assessment of individuals' general feelings of connection to the natural world and other (nonhuman) living organisms (Mayer & Frantz, 2004).

***Tellegen Absorption Scale* (TAS):** The TAS is a 34-item true-false questionnaire used to measure sensitivity to altered states of consciousness (Tellegen, 1982), which may be a marker for sensitivity to psilocybin (Studerus et al., 2012) and which may increase after psilocybin.

***Measure of Actualization of Potential* (MAP):** This questionnaire assesses the construct of self-actualization, a process through which one's potential is developed in congruence with one's self-perception and experience (Lefrancois et al., 1997; Leclerc et al., 1998, 1999).

TCI Self-transcendence subscale: The self-transcendence subscale of the Temperament and Character Inventory-Revised (Cloninger et al., 1993; Cloninger, 1999) is a 26-item self-report measure used to assess an individual's sense of connectedness to their surroundings, a stable personality trait correlated with related personality constructs such as openness (McCrae, 2009).

***Five Facet Mindfulness Questionnaire* (FFMQ):** This is a questionnaire scale assessing mindfulness in daily activities (Baer et al., 2006).

Outcome measures to be assessed in the study are listed in the following table.

Time of Measure (approximate)	Measure
Completed daily by participant at home from 2 weeks before the Target Quit Date until end of study treatment.	• Smoking diary
The morning of psilocybin session (before psilocybin administration)	• Pre-Session Questionnaire<br>• Staff Rating of Likely Experience
Throughout psilocybin session (10 min. before capsule, and 30, 60, 90, 120, 180, 240, 300 min. after capsule)	• Within-session Monitor Rating Form<br>• Heart rate / Blood pressure<br>• Video and audio recordings
About 7 hours after psilocybin administration	• Mystical Experience Questionnaire (MEQ)<br>• Hallucinogen Rating Scale (HRS)<br>• Post-session Monitor Rating Form
The day after psilocybin session	• Next-day Monitor Rating / Headache Interview
At End of Treatment (8 weeks after TQD)	• COVID-19 Protocol Feedback
Longitudinal measures (timing specified by superscripted number below)<br><br>[1] At baseline screening, at TQD, at all study treatment meetings, and at 3, 6, and 12 month follow ups<br><br>[2] At baseline screening, 8 weeks after TQD, and 3, 6, and 12 month follow ups<br><br>[3] At baseline screening<br><br>[4] At baseline screening, and 3, 6, and 12 month follow ups<br><br>[5] At baseline screening and 1 week after TQD<br><br>[6] At 1 week after TQD<br><br>[7] At 8 weeks after TQD, 3, 6, and 12 month follow ups<br><br>[8] At baseline screening, at each treatment meeting, and at 3, 6, and 12 month follow ups<br><br>[9] At baseline screening, week 2, week 5 of treatment, and 3-month follow-up*<br><br>[10] At baseline screening, 1 week after TQD, 8 weeks after TQD, and 6 month follow up<br><br>[11] At baseline screening, 8 weeks after TQD, 6 and 12 month follow up<br><br>[12] At baseline screening, at TQD, at 1 through 8 weeks after TQD, 3, 6, and 12 month follow up<br><br>[13] At baseline screening, 1 week after TQD, and 6 month follow up<br><br>[14] At baseline screening, 6, and 12 month follow ups<br><br>[15] At baseline screening, 2 weeks after TQD, and 6 month follow up<br><br>[16] At baseline screening, 4 weeks after TQD	Smoking related:<br>• Carbon monoxide (CO) reading [8]<br>• Urine cotinine sample [4]<br>• Smoking History Questionnaire [4]<br>• Smoking Abstinence Self-Efficacy (SASE) [8]<br>• Wisconsin Smoking Withdrawal Scale (WSWS) [12]<br>• Questionnaire on Smoking Urges (QSU) (32 item) [8]<br>• Fagerström Test for Nicotine Dependence (FTND) [3]<br>• Smoking Cessation Contemplation Ladder [8]<br>• Timeline Follow-Back (TLFB) [1]<br>Other measures:<br>• General Self-Efficacy Scale (GSES) [10]<br>• Brief Symptom Inventory (BSI) [2]<br>• PANAS-X [2]<br>• Beck Depression Inventory (BDI) [2]<br>• State-Trait Anxiety Inventory (STAI) [2]<br>• Connectedness to Nature Scale (CNS) [10]<br>• Tellegen Absorption Scale [10]<br>• Toronto Alexithymia Scale [10]<br>• TCI Self-transcendence subscale [10]<br>• MAP (Measure of Actualization of Potential) [10]<br>• Five Facet Mindfulness Questionnaire [10]<br>• Schwartz Value Scale [10]<br>• Purpose In Life Test (PIL) [2]<br>• Delay / Health discounting & Elasticity of Demand [13]<br>• Zimbardo Time Perspective Inventory (ZTPI) [10]<br>• Future Time Perspective (FTP) [11]<br>• Visual Effects Questionnaire [4]<br>• Hood Mysticism Scale (lifetime) [14]<br>• Blood markers: Cortisol, ACTH, IL-4, IL-6, IL-10, INF-gamma, TNF-alpha, Gs-alpha [5]<br>• Persisting Effects [6]<br>• States of Consciousness Retrospective Questionnaire [7]<br>• Adverse Events form [1]<br>• Open-ended clinical Interview [7]<br>• Monitor Rating of Enduring Effects [7]<br>• Saliva samples for cortisol [5]<br>• Functional Neuroimaging: MRI scanning [9 †]

[†] MRI scanning procedures will occur only for those individuals who are deemed MRI eligible and consent to do so (n=60).
*3-month follow-up MRI Scan 3 only for MRI participating volunteers showing biologically verified 7-day point-prevalent abstinence at that time.

***Schwartz Value Scale* (SVS):** This 57-item questionnaire is a multidimensional self-report measure assessing the relative importance of various life values (Schwartz, 1992, 1994).

***Purpose in Life Test* (PIL):** This 20-item questionnaire assesses a participant's sense of meaningfulness in life (Crumbaugh and Maholick, 1964).

Delay discounting: This measure assesses impulsivity by asking participants to make hypothetical choices between smaller immediate rewards and larger later rewards (e.g., would you rather receive $500 now or $1000 after waiting 1 year?). This measure has been found to relate to nicotine dependence in several studies (Bickel & Johnson, 2003). A delay discounting assessment based on health outcomes will also be administered (Baker et al., 2003).

***Zimbardo Time Perspective Inventory* (ZTPI):** This validated questionnaire assess the construction of psychological time, and includes 56 questions scored on a 5-point scale with subscales assessing past negative, present hedonistic, future, past positive, and present fatalistic dimensions (Zimbardo & Boyd, 1999).

***Future Time Perspective* (FTP):** Participants will complete task I from this questionnaire designed to assess future time orientation (Wallace, 1956; Petry et al., 1999). Participants are asked to list 10 events that may happen to the participants during the rest of their lives. After each written response, the participant is asked what age he or she might be when each event will occur. Unknown to the participant, the dependent measure is not the events listed, but the average number of years in the future in which the events will occur. Drug dependent individuals have been found to provide shorter distances into the future compared to non-drug dependent individuals on this task (Petry et al., 1999).

***Visual Effects Questionnaire*:** This form was used in our first psilocybin study (Griffiths et al., 2006) to assess any persistent perceptual effects resulting from sessions (e.g., seeing auras).

Hood Mysticism Scale (lifetime): This 32-item questionnaire was developed to assess primary mystical experiences. The Mysticism Scale has been extensively studied, demonstrates cross-cultural generalizability, and is well-regarded in the field of the psychology of religion (Hood et al. 2001; Spilka et al. 2003). A total score and three factors are scored: Interpretation (corresponding to three mystical dimensions described by Stace [1960]: noetic quality, deeply felt positive mood, and sacredness); Introvertive Mysticism (corresponding to the Stace dimensions of internal unity, transcendence of time and space, and ineffability); Extrovertive Mysticism (corresponding to the dimension of the unity of all things/all things are alive). Items are rated on a 9-point scale. The lifetime version of the questionnaire instructs participants to answer questions with reference to their experiences over the course of their lifetime. This scale was shown sensitive to the effects of psilocybin in our previous study (Griffiths et al., 2006).

***Blood markers of stress/immune function*:** Cortisol, ACTH, IL-4, IL-6, IL-10, INF-gamma, TNF-alpha: To assess these markers 10 cc blood samples will be taken at baseline (a total of 30 cc blood is taken at baseline, including 20 cc for general medical screening), and at 1 week after TQD. The blood draw at 1-week after TQD will be optionally completed if participants are able and willing to come to BPRU in person for blood draw procedures. It has been hypothesized that hallucinogen facilitated mystical experiences may be accompanied by decreased stress and may improve immune function (Roberts, 1999; Roberts, 2006), however smoking cessation may be a

confounding factor. We have therefore included these easily obtained and inexpensive assays as an exploratory step to determine if changes are observed within subjects and between treatment conditions.

<u>Gs-alpha protein in blood:</u> The Gs-alpha protein has been hypothesized to play a role in mood altering effects of drugs (Donati et al., 2015). Thus, from pre-post blood samples we will extract red blood cells (RBC) and platelets from cells and prepare membranes from both fractions. For RBC, we will determine the extent to which Gs-alpha is ensconced in lipid rafts pre- and post-psilocybin treatment. For both RBC and platelets, we will determine the proportion of Gs-alpha that is palmitoylated. The former studies will be done by immunodetection and the latter by mass/spec analysis.

Saliva samples for cortisol assessment: In addition to blood cortisol collection as described above, we will also collect saliva samples for cortisol analysis (e.g., Lee et al., 2007; Varadhan et al., 2008). Diurnal amplitude of cortisol, which takes into account a morning and evening measure of cortisol within a 24 hour period, is known to to be a meaningful index of stress (e.g., Varadhan et al., 2008). Collecting these morning and evening levels via saliva allows the participant to collect samples at home, as has been successfully performed in previous research at Johns Hopkins (e.g., Varadhan et al., 2008). At baseline assessment, and 1 week after TQD, participants will be sent home with commercially available Salivette swabs (Sarstedt Inc, Newton, North Carolina). Participants will use these swabs to collect one sample in the evening before bedtime but at least 1 hour after eating, and another sample the next morning within 30 minutes of waking but before brushing teeth. For each sample, the swab is chewed lightly for 45 seconds and returned to its container. The two labeled samples will be returned to the laboratory on the next scheduled visit. Because identical procedures will be performed at baseline, and 1 week after TQD, a total of 4 swabs (2 per occasion for evening and morning) will be obtained.

Open Ended Clinical Interview: Brief interviews (approx. 60 min.) will be conducted during which participants will be asked to share any positive or negative effects from their study participation which have not been covered by other questionnaires. These interviews will be conducted by study team members during regularly scheduled counseling meetings, and may be audio recorded for purposes of qualitative data analysis.

States of Consciousness Questionnaire: (Pahnke, 1969; Richards, 1975; Richards et al., 1977): This scale was designed to assess mystical experiences, was used as a primary outcome measure in the Good Friday Experiment (double blind study of psilocybin; Pahnke, 1963; Doblin, 1991), and has been shown to be sensitive to hallucinogens, including psilocybin in our previous study (Pahnke, 1969; Turek et al., 1974; Richards, 1975; Richards et al., 1977; Griffiths et al., 2006).

Persisting Effects Questionnaire: This 145-item questionnaire was designed to measure changes in attitudes, moods, behavior, and spiritual experience, and has demonstrated sensitivity to the intermediate and long-term effects of psilocybin (Griffiths et al., 2006, 2011). The questionnaire has been modified for this study to additionally assess attributions volunteers make retrospectively to their treatment experience, and how it may have facilitated smoking cessation.

Monitor Rating of Enduring Effects: This questionnaire will assess the monitor's observations of persisting effects of study participation on participant attitudes and behaviors.

***States of Consciousness Retrospective Questionnaire*:** This questionnaire has been used in our previous psilocybin research (Griffiths et al., 2006) for purposes of long-term follow-up. Some items on this questionnaire comprise the States of Consciousness Questionnaire, and are completed retrospectively for the experience. Other items comprise the previously described Persisting Effects Questionnaire. For these items, volunteers are asked to rate any current persisting effects that they attribute to the experience. Within the Retrospective Questionnaire, volunteers are also asked to provide written descriptions of what was most memorable and what was most spiritually significant about the experience.

Adverse Events Form: After TQD participants will be asked at each study treatment meeting to report on how they have been feeling since their last in-person meeting. In the case of any adverse events (AEs), these will be documented using an AE form, which assesses the nature of AE, duration, severity, and what course of action was taken to address the event.

COVID-19 Protocol Feedback: At the end of treatment (~8 weeks after TQD) participants will be asked to complete a brief questionnaire designed by our team to assess the perceived impact of COVID-19 related study procedures (e.g., mandatory COVID-19 testing, masks / PPE, virtual meetings) on key elements of their study experience (e.g., psilocybin session, relationship with study team, quit attempt, sense of safety).

Functional Neuroimaging (fMRI; for MRI participating volunteers only):
Smoking cessation and withdrawal have been well documented to affect brain function, including resting state functional connectivity, and smoking related cue-reactivity (McLernon et al., 2007; Sutherland et al., 2012). In order to examine the underlying neurobiological mechanisms associated with psilocybin-facilitated smoking cessation, and identify potential brain based mediating factors predictive of treatment outcomes, up to 60 participants will undergo two to three brain scans collecting fMRI data while performing the tasks detailed below. The first scanning session will take place in week 2 of treatment, after a 24-hour period of smoking abstinence. The second will occur in approximately week 5 of treatment (i.e., one day after the psilocybin session, or immediately before initiating NRT), after another 24-hour period of smoking abstinence. Participants who provide biologically verified (i.e., breath CO), and self-report seven-day point prevalence abstinence at 3 months post-TQD will receive a final follow-up MRI scan at that time. Functional neuroimaging will be conducted at NIDA IRP by trained MRI operators under the supervision of Dr. Amy C. Janes. In addition to these scanning sessions, MRI participating volunteers will also be given the option to participate in a preliminary MRI scan through a NIDA-approved sub-study protocol. Either the primary study scans or the preliminary study scan will include a clinical scan to be examined for incidental findings.

State of the art, multimodal imaging will be performed on a Siemens 3T Trio scanner, located at the NIDA-IRP (intramural research program). Participants' heads will be placed inside the RF coil and held in place by a head restraint that generally consists of soft foam padding. For most scanning experiments, a blipped, gradient-echo, echo-planar image (EPI) pulse sequence (e.g. TE = 27ms) is used with a TR of about 2 seconds. Specific imaging parameters (e.g. number of slices, slice angle, TR, TE, flip angle etc.) may be changed in order to maximize signal integrity. A "watchdog" program runs continuously on the MRI computer during all pulse sequences and ensures that no scanning parameter ever exceeds FDA guidelines.

Structural Images: A set of high resolution structural MR images will be collected. The images will be acquired using a T1-weighed three-dimensional MPRAGE sequence and will be used for the display of functional data. Completion time: ~5 min.

Resting Scan. An EPI scan to assess resting-state fluctuations (RSF) will be conducted. These scans will provide us with information relating to trait and perhaps state connectivity between different prefrontal and limbic brain regions. Completion time: ~10 min.

Cue-elicited Craving: Participants are presented two kinds of stimuli, smoking-related picture cues and neutral pictures (see Fig. 1). The smoking-related pictures are selected from Mucha's study (Mucha et al., 1999) and the International Smoking Image Series (Gilbert and Rabinovich, 1999) and similar smoking and neutral images from open source internet sites. The content of neutral cues are paired to smoking-related cues, but eliminate any smoking-related information.

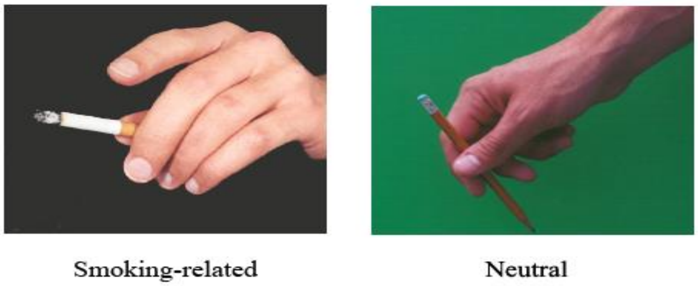

Fig. 1: Sample stimuli. One representative pair is showed above.

Amygdala Reactivity Task: Participants will complete a simple perceptual task previously shown to produce robust bilateral amygdala activity (Pezawas et al., 2005; Hariri et al., 2002; Foland-Ross et al., 2010), as well as top-down prefrontal regulation of amygdala reactivity (Hariri et al., 2002; Foland-Ross et al., 2010). This blocked fMRI paradigm consists of two experimental conditions: a face matching condition, and a sensorimotor control condition. During the emotion matching condition, participants view an array of 3 faces and their task is to select (via button press) one of the two faces that matches the identity of the target face. During the sensorimotor control task, participants view an array of 3 geometric shapes (circles, vertical and horizontal ellipses) and select one of the two shapes (bottom) that matches the target shape (top). Each block begins with a brief instruction screen ("match face" or "match shape"). During each emotion block different images selected from a standard set of pictures of facial affect (Lang et al., 2001), are presented. During the control blocks, different geometric shapes are presented in a pseudo-random order. Completion time: ~7min

Multisource Interference Task (MSIT): The MSIT is a cognitive control task that reliably and robustly activates the anterior cingulate cortex and fronto-parietal executive control and attention network (Bush & Shin, 2006). During each trial participants view three numbers (e.g., 2 1 1) and are required to identify the "oddball" number which differs from the other two (i.e., 2) as quickly as possible by pressing the corresponding number on a 3 button response box (1, 2, 3). On some trials the location of the oddball is the same as the location on the response box (e.g., 2 2 3) and sometimes it is different (e.g., 2 1 1). This latter trial type produces a slowing in response time that reflects conflict produced by flanker stimuli and the need to respond to stimulus identity (i.e., press 2) and suppress the response to stimulus order (i.e., press 1). Completion time 15min.

Central executive function of attentional switching within verbal working memory task*:* Our previous work (Garavan et al, 2000) revealed widespread activation associated with attention switching within working memory (WM). Subjects will perform a verbal WM counting task. In each trial, subjects will be presented with a series of large and small squares and, using a response device, must report final counts at the end of each trial. The order in which the squares are presented dictates whether an update of the same count is required (e.g., two large squares in succession requires two updates of the “large square” count) or whether subjects must switch attentional focus to update the “other” count (e.g., a large square followed by a small square requires an update of the “large square” count followed by a switch to the “small square” count and an update of it). A parametric manipulation will produce trials with different numbers of switches, thus, for each particular trial length there will be a LOW, MEDIUM, and HIGH switching density trials ~ 10 min.

N-back task: The n-back visual working memory task (Kane et al., 2007) will be administered to assess working-memory performance. During the n-back task participants are presented with a series of single letters in a pseudorandom order and are required to decide whether the current stimulus matches that which appeared n items previously (Kane et al., 2007). ~20min

Dot Probe Task: A dot-probe task will be administered outside of the MRI scanner to assess for attentional bias to smoking-related stimuli (Ehrman et al., 2002). Each trial of the task begins with a fixation cross followed by a brief (~500ms) presentation of two images, one smoking-related and one neutral image either side of the fixation cross. Immediately after the offset of these images a target (dot probe) appears either in the position where the smoking-related image was, or where the neutal image was. Participants are required to respond as quickly as possible to the dot probe and the latency to respond provides a behavioral measure of how much attention was directed to either the preceding smoking-related or neutral image. For example, if relatively greater attention is directed to smoking-related images, responding will be faster on trials where the probe appears behind the smoking-related image and slower on trials where the probe appears behind the neutral image. Completion time: ~5min

Measures taken the morning of psilocybin session prior to drug administration (for psilocybin condition only):

Pre-Session Questionnaire: To assess the psychological “set” of the volunteer before the session, subjects will complete a brief questionnaire before psilocybin administration on which they will rate various dimensions of psychological set (e.g., the extent of pre-session apprehension, preoccupation with thoughts and problems not related to the session, positive expectation).

Staff Rating of Likely Experience**:** Study staff will complete a questionnaire before the session rating their expectations about the likely nature of the drug experience for that subject (e.g. likelihood that the experience will be interpreted as being spiritually significant or personally meaningful, or will involve a period of significant fear). This form was used in our previous psilocybin study (Griffiths et al., 2006).

Measures taken throughout psilocybin session (10 min. before capsule, and 30, 60, 90, 120, 180, 240, 300 min. after capsule; psilocybin condition only):

Within-session Monitor rating form: At the same timepoints at which the heart rate and blood pressure will be taken, the two study staff will independently complete the Monitor Rating Form, which we used in our first psilocybin study. The form involves rating or scoring 26 dimensions of the subject's behavior and mood (e.g., yawning, minutes sleeping, minutes speaking, nausea/vomiting, motor activity, sedation, anxiety/fear, paranoia, crying, sorrow/sadness, joy/intense happiness, peace/harmony, overall drug effect).

Heart rate and blood pressure: At 10 minutes before and 30, 60, 90, 120, 180, 240, and 300 minutes after psilocybin administration, heart rate and blood pressure will be monitored for safety and because psilocybin is known to produce modest but reliable elevations in blood pressure (Griffiths et al., 2006; Gouzoulis-Mayfrank et al., 1999b; Hasler et al., 2004).

Video and audio recordings: Video and audio recordings, which will be taken throughout the psilocybin session, will be reviewed by investigators. Video and audio recordings will permit an objective assessment of session activity and will provide a rich source of additional data about subject comments and behavior during the session that may be useful in characterizing the effects of psilocybin. Video and audio recordings will be treated as confidential research materials and will be stored in locked offices.

<u>Measures taken about 7 hours after psilocybin administration:</u>

Mystical Experience Questionnaire **(MEQ; MacLean et al., 2012):** This 30 item scale is an abbreviated version of the 100 item States of Consciousness Questionnaire (Pahnke, 1969; Richards, 1975; Richards et al., 1977). The MEQ was derived via exploratory factor analysis, and is intended to characterize the impact of single hallucinogen-occasioned drug sessions for common elements of mystical experience (e.g., unity, sacredness, transcendence of time/space, ineffability).

Hallucinogen Rating Scale **(Strassman et al. 1994; Riba et al. 2001):** This questionnaire, which was designed to show sensitivity to the hallucinogen N,N-dimethyltryptamine, consists of six subscales assessing various aspects of hallucinogen effects (intensity, somaesthesia,, affect, perception, cognition, and volition). This scale was shown sensitive to the effects of psilocybin in our previous study (Griffiths et al., 2006).

Post-session Monitor Rating Form**:** On this form, which was used in our previous study of psilocybin (Griffiths et al., 2006), monitors will rate the qualitative nature of the session at the conclusion of each session.

<u>Measure taken the day after psilocybin session (psilocybin condition only):</u>

Next-day Monitor Rating Form**:** On this form, which was used in our previous study of psilocybin (Griffiths et al., 2006), study staff will rate the qualitative nature of the session the day after each session after meeting with the volunteer.

Headache Interview Form: This 14-item interview assesses incidence, duration, and severity of headache during or immediately after psilocybin sessions, as headaches have been found to occur in some individuals post-psilocybin (Johnson et al., 2012).

c. Statistical plan including sample size justification and interim data analysis.

This is a pilot comparative efficacy study with a randomized control condition. Demographics and smoking history at baseline will be examined for between group differences using Fisher's exact tests and independent-samples t-tests. The primary outcome variable will be a between group comparison of the percent of volunteers who show biologically verified (via carbon monoxide and cotinine measures) and self-reported smoking abstinence post-TQD, allowing for a grace period for initiation of prolonged abstinence within 14 days after the target-quit date in line with current recommendations for best practices in smoking cessation clinical trials (Piper et al., 2020). For this primary analysis, positive cotinine samples will be counted as smoking abstinent if the person indicates on the TLFB that they have used non-smoking forms of nicotine. This comparison will be performed using a logistic regression analysis, with treatment condition as the predictor variable and prolonged abstinence as the examined outcome. Although the study sample will be small, these results will provide useful preliminary data indicating whether follow up investigation in this area is warranted. Between groups comparisons of secondary measures will examine differences across treatment conditions on variables such as smoking urges and withdrawal using independent-samples t-tests. We will apply a 2-step approach when assessing between-group differences in these variables. First, we will compare values between all participants still enrolled in the study at 6-months post-TQD, then only among those still smoking at 6 months post-TQD according to 7-day point prevalence criteria. In order to rule out the role of alternative sources of nicotine in achieving smoking abstinence, we will perform a secondary analysis where participants who stopped smoking tobacco cigarettes and initiated alternative forms of nicotine use (e.g., e-cigarettes, smokeless tobacco) will be coded as non-abstinent.

Neuroimaging data will be analyzed to account for differences in BOLD signal and functional connectivity related to task parameters between groups. Resting state MRI will be assessed between groups and within-subjects as a function of relapse status to examine whether characteristics of resting state functional connectivity (rsFC) are associated with treatment success (vs. failure). Additionally, behavioral performance on each of the fMRI tasks assessing working memory, attention, processing speed, inhibitory control processes, cognitive control, reward responsiveness, amygdala, striatal, and impulsive decision making (e.g., reaction time, error rate, hit rate, reward bias) will provide task-related parameters necessary for analysis of BOLD and resting state data. Self-reported craving, withdrawal symptoms and mood/affect will also be employed as regressors in the analysis of task and resting BOLD data. No interim data analyses are planned.

d. Early stopping rules.

The investigators will monitor safety data and relevant literature and report information to the IRB that might increase the risk assessment of the study.

8. Risks
a. Medical risks, listing all procedures, their major and minor risks and expected frequency.

<u>Smoking Withdrawal:</u> Participants who quit smoking will likely experience nicotine withdrawal symptoms, which are expected to last approximately 2-4 weeks and peak within the first week after quitting. Symptoms might include craving cigarettes, restlessness, irritability,

increased appetite, increased eating, dizziness, difficulty concentrating, nausea, anxiety, sleep disturbance, and depressed mood (Hughes, 2007). Long-term smoking is associated with increased health risks such as cancer and cardiovascular disease. Therefore the risk of discomfort resulting from nicotine withdrawal is outweighed by the long-term health benefits likely with sustained abstinence.

MRI Scan Risks (for MRI participating volunteers only): Participants may find the tasks that they are asked to perform during, before and after MRI scanning sessions boring, difficult, and/or frustrating. In order to reduce discomforts associated with performing the emotional and cognitive tasks, scanning sessions and task sessions outside of the scanner are organized in such a way that participants will not perform tasks for extended periods of time and will be given a chance to rest between tasks. In addition, the tasks are designed to make performance neither too difficult nor too easy. Ideally, individual adjustments of task difficulty enable participants to remain actively engaged in the task. Participants receive extensive training before the actual experimental task begins.

When used on appropriately qualified individuals, MRI presents minimal risk as long as technical scan parameters remain within FDA guidelines. There is no exposure to x-rays or radioactivity. The radio waves used have produced burns (most of these minor) in about one in a million exams. The magnet may move metal implants in the body, the motion of which could be painful and harmful. Metal implants may also cause burns from the radio frequency energy used. While inside the magnet, subjects may experience an acute panic attack due to claustrophobia. Subjects may also experience mild, remittable discomfort from lying in the scanner. To ensure adherence to FDA guidelines for MRI, a trained MRI operator who has been instructed in these guidelines performs all machine manipulations. Other preventative measures include assuring that all equipment to be used during imaging sessions is MR compatible, that participants are familiar with the MRI environment (using the mock scanner), and that participants are aware of how to signal the MR operator if they need to do so during the session. Furthermore, participants are screened prior to each MRI session for any MR contraindications, including metal implants, pregnancy, fear of small, enclosed spaces, and inability to lie still for prolonged periods of time.

Some subjects may experience temporary, reversible shifts in hearing threshold after MRI. The sound generated by an MR system usually consists of a series of repetitive pulses. Acoustic noise is a result of the mechanical vibration produced by the gradient coils when the large currents are applied to them to create time varying imaging gradient fields. The relevant safety parameters required to characterize such a noise are the peak impulse sound pressure level (L-peak) and the time integral of the A-weighted sound pressure level (Leq). In MR applications, the peak impulse sound pressure level is dependent upon the peak amplitude of the individual pulses, while the time integral of the A-weighted sound pressure level is dependent upon the continuous exposure to a series of such pulses. Based on Occupational Safety and Health Administration (OSHA, Occupational Noise Exposure – 1910.95, www.osha.gov) regulations: 1) exposure to impulsive or impact noise should not exceed 140 dB peak sound pressure level, and 2) exposure to continuous noise for 2 hours per day should not exceed 100 dB. Note that these regulations are based on employees' daily exposure to a noisy environment over their entire working career, whereas we use them here as a comparison for participants' occasional exposure to MRI scanner noise, thus making these values extremely conservative measures. Measurements within our MRI scanner (provided by Siemens, the manufacturer) indicate that the maximum noise level produced by our standard fMRI pulse sequence is 116.5 dB. Based upon our typical echo spacing, this sound is in the 3-4 kHz range. According to the Occupational Safety and Health Administration regulations (OSHA, occupational noise exposure 1910.95,

www.osha.gov): 1) Exposure to impulsive or impact noise should not exceed 140 dB peak sound pressure level, and 2) Exposure to continuous noise for 2 hours per day should not exceed 100 dB. These guidelines are based on employees' daily exposure to a noisy environment over their entire working career, whereas we use them here as a comparison for participants' occasional exposure to MRI scanner noise. This study consists of a series of scans ranging in length from a few seconds to about ten minutes. Rest periods of up to a few (1-5) minutes occur regularly between scans while the next scan is being set up.

During fMRI experiments, MRI compatible headphones with tubes for audio input and/or earplugs with or without tubes for audio input are routinely used for hearing protection. In addition, a vacuum pillow or other padding used for head stabilization may also attenuate noise. Based on the manufactures specifications, the earplugs with and without audio tubes have noise reduction ratings of approximately 25dB and 33 dB. The headphones have a noise reduction rating of 29 dB. The vacuum pillow / padding foam provides additional noise reduction (values not available, but estimated at 5-15 dB). The noise reduction rating is a conservative measure that averages over frequencies and attempts to account for improper protection use. The mean noise attenuations at 3150 Hz, a frequency near that of our scanner, are actually 35dB, 47dB for the 2 types of earplugs and 40dB for the headphones used. Thus, the use of any two of these devices in combination reduces the noise level to levels much lower than that required by OSHA for lifetime exposure, and provides effective hearing protection for the participants. Nevertheless, some subjects may experience temporary, reversible shifts in hearing threshold after MRI.

Incidental Findings (for MRI participating volunteers only)**:** Sometimes there are unexpected findings on an MRI scan. Some findings are of unknown significance or importance to participants' health, which may make them anxious and lead to further tests that may be unnecessary. We will inform participants about any finding that may require further evaluation or care. If needed, we will refer participants to a health care provider. If something unexpected is found, it may make it harder or more expensive for participants to buy life insurance or disability insurance. The costs for any care that will be needed to diagnose or treat an incidental finding would not be paid for by this research study. These costs would be the participants' responsibility.

NRT Adverse Effects: The most commonly reported adverse effects of nicotine transdermal patch use include skin irritations, gastrointestinal complaints, heart palpitations, chest pains, and insomnia (Mills et al., 2010). Discontinuation rates due to adverse effects for nicotine patch are generally lower than those of other smoking cessation treatments in clinical trials (Aubin et al., 2008; Moore et al., 2009). Current data indicates that NRT can be safely administered over the counter, and that it does not pose a known risk in terms of human abuse liability (West et al., 2000). A complete list of adverse effects will be provided to all participants (FDA label, PDR.net).

Psilocybin Adverse Effects: Preclinical and clinical data show psilocybin to have a safe profile of physiological toxicity, as described below (in the section Psilocybin Pharmacology and Toxicology). Psilocybin and related hallucinogenic compounds were investigated in humans in numerous clinical research studies in the 1950s and 1960s. The incidence of adverse effects in these studies was very low, especially when participants were carefully screened, prepared, supervised, and followed up (Cohen 1960; Bergman, 1971; Strassman 1984). In previously described reviews of relative dependence potential and lethality for 20 commonly used

psychoactive drugs, Gable (1993, 2004) concluded that heroin was associated with the greatest relative toxicity while psilocybin was associated with the least.

Acute Psychological Effects of Psilocybin: The most likely potential adverse effect of psilocybin is anxiety, or possibly panic, during the period of acute drug action. The preparation and close supportive supervision provided to participants will minimize both the probability of such transient effects and, should they occur, the risk of any harmful consequences. Some individuals have been reported to develop paranoid and psychotic states while under the influence of hallucinogens, including psilocybin (Strassman 1992; IND 39,258, Strassman personal communication; Nielen et al., 2004). It is generally believed that individuals with previous psychiatric conditions, particularly psychosis or mania, are at an increased risk for hallucinogen-induced psychosis (Abraham et al., 1996). Furthermore, the risk that psilocybin could precipitate in vulnerable individuals enduring psychiatric illness such as schizophrenia is very low, but cannot be excluded completely (Abraham et al., 1996). For this reason, all potential participants with personal or family histories of schizophrenia, psychotic disorders, or bipolar I or II disorder will be excluded from participation. Nevertheless, while we believe the risk to be minimal, it is possible that some participants may develop a brief psychotic reaction while under the influence of psilocybin. Such a reaction will be handled as described below under medical emergency procedures. To our knowledge, there have been no reports of persistent psychotic states associated with psilocybin (in contrast to LSD, which has a different pharmacological profile) (Abraham et al. 1996).

In a previous human trial (Strassman 1992; IND 39,258, Strassman personal communication), one adverse psychological episode, involving transient anxiety and paranoia, was reported to FDA. The participant recovered quickly, and showed no long-term deleterious consequences of the episode. Our research team has been in personal communication with Dr. Strassman about this participant’s experience, and consistent with his recommendations we have designed our protocol to minimize the likelihood of a similar event. In other recent studies of psilocybin in humans (Hasler et al. 1997, 2004) including our studies, no adverse reactions other than the expected occasional episodes of fear or anxiety during the time of drug effect have been reported. Such transient episodes of fear or anxiety respond well to reassurance and have not required pharmacological or physician intervention.

Psilocybin Pharmacology and Toxicology: Psilocybin and its analogues (psilocin, CEY-19, and CZ-74) are all psychoactive indoles with side-chains that place them within the general class of tryptamines. These compounds are analogues of hydroxytryptamine (5-HT, serotonin, a naturally-occurring neurotransmitter). The psychopharmacological mechanism of psilocybin and its analogues is similar to LSD and mescaline in that all these compounds seem to act as post-synaptic agonists at 5HT2A and 5HT2C receptors (Sanders-Bush & Mayer 1996). In healthy human participants psilocybin increased thyroid stimulating hormone, adrenocorticotropic hormone, and cortisol, and moderately elevated blood pressure (Hasler et al. 2004). Psilocybin subjective effects in healthy participants were blocked by the 5HT2A antagonist ketanserin as well as by the mixed 5HT2/D2 antagonist risperidone (Vollenweider et al. 1998). Studies using positron emission tomography (PET) showed that psilocybin increased frontal glucose metabolism in healthy participants, suggesting that the behavioral effects of psilocybin involve the frontal cortex (Vollenweider et al. 1997; Gouzoulis-Mayfrank et al. 1999a). In a recent review of the pharmacology of psilocybin, Passie et al. (2002) concluded that psilocybin exhibits low toxicity and is physiologically well-tolerated.

Animal studies suggest very low levels of general medical toxicity. The LD50 in rodents is approximately 2,000 to 3,000 times the usual human dose on a mg/kg basis (Usdin & Efron 1972; Smith et al., 1996), comparing favorably with drugs such as aspirin and caffeine. Psilocybin and related hallucinogenic compounds were investigated in humans in numerous clinical research studies in the 1950s and 1960s. The incidence of adverse effects in these studies was low, especially when participants were carefully screened, prepared, supervised, and followed up (Cohen 1960; Strassman 1984). Gable (1993) reviewed over 350 research reports on the non-medical use of 20 psychoactive drugs (including alcohol and nicotine) in humans to compare these drugs in terms of their dependence potential and acute toxicity as rated by 24 toxicologists who had published significant research in the area of abusable drugs. Results showed that the extremes were occupied by heroin (having the highest levels of acute toxicity and dependence potential) on the one hand and by psilocybin (having the lowest levels of both) on the other. In a recent follow up review and analysis of 3000 citations, Gable (2004) calculated safety ratios for 20 psychoactive compounds, comparing lethal doses to commonly used doses, and found heroin was associated with the greatest toxicity and psilocybin the least.

<u>Physiological Safety Data in our Previous Studies of Psilocybin in Healthy Participants:</u>
Physiological effects of psilocybin consisted of moderately increased heart rate and blood pressure. The mean increase in heart rate was 19.8 beats per minute, the mean increase in systolic blood pressure was 31.4 mm Hg, and the mean increase in diastolic blood pressure was 17.9 mm Hg. In terms of psychological adverse effects, 4 of the 36 volunteers reported the psilocybin session to be dominated by anxiety and dysphoria. An additional 4 participants reported that a significant portion of her/his psilocybin session was characterized by anxiety or dysphoria. These effects were easily managed with reassurance, and did not persist beyond the acute effects of the drug during the session. No volunteer reported the psilocybin session to have decreased her/his sense of well-being or life satisfaction. Minor adverse effects that may be associated with psilocybin administration are dizziness, nausea, vomiting, incontinence, and muscle twitching. During the period of drug action, there may be an increased risk of fall or other injury due to incoordination, misjudgment, or other effects of psilocybin. To mitigate this risk we have implemented new safeguards for session monitors as described in “steps taken to minimize risks.” Self-report data from online case reports after using psilocybin mushrooms recreationally (www.erowid.org; www.bluelight.ru; www.drugs-forum.com) and a summary report of psilocybin side-effects from previously published psilocybin studies (Studerus et al., 2011) suggest that next-day leg or muscle pain is a possible effect. There may be an association between psilocybin administration and transient headache starting about 7 hours after administration (Johnson et al., 2012; Studerus et al., 2011).

<u>Non-Medical Use of Psilocybin:</u> Varieties of mushrooms containing psilocybin as their primary active chemical are currently in use in religious rituals of native Americans (mainly in Mexico and Central and South America); this use is documented as early as the sixteenth century and probably reaches back into pre-Columbian times (Hofmann 1983). As reported in books, articles, and Internet reports, contemporary Western individuals and groups make use of psilocybin mushrooms with “spiritual” intent in settings and rituals inspired by indigenous use and in settings of contemporary design. However, along with other hallucinogens popular in contemporary American culture, psilocybin also continues to be a drug used for casual, unsupervised experimentation.

In the United States, although psilocybin (in the form of psilocybin-containing mushrooms) is the second most commonly used of the classic hallucinogens, which include

LSD, psilocybin, and mescaline (SAMHSA 2004b), psilocybin has not resulted in significant public health problems and medical emergencies resulting from its use are very rare (psilocybin is mentioned in only 0.1% of drug-related emergency department visits) (SAMHSA 2004a). The incidence of prolonged adverse psychological reactions to LSD, which is both a more potent and a longer-acting hallucinogen than psilocybin, was found to be very low in a follow-up survey of 1,250 non-patient participants who received LSD under supervised research or therapeutic conditions (Cohen 1960). The single adverse event occurred with a participant who was an identical twin of a schizophrenic, and thus who would have been screened out of our present study. The adverse event, involving anxiety and depersonalization, resolved within five days.

Concern that Psilocybin Administration Will Increase Drug Abuse: A potential concern about the proposed trial is the utilization of a drug with a known history of abuse (psilocybin) in the treatment of dependence on another drug of abuse (tobacco). Like many classes of psychoactive drugs, classical hallucinogens such as psilocybin are sometimes used in a manner that jeopardizes the safety or well-being of the individual or others (e.g., driving while impaired; a pattern of use that interferes with work, school, or relationships). Under such circumstances hallucinogens are said to be *abused*. However, hallucinogens are not typically considered drugs of *dependence* in that they do not engender compulsive drug seeking (National Institute on Drug Abuse, 2001, 2006; O'Brien, 2006), consistent with the observation that they are not reliably self-administered in nonhuman animals (Poling and Bryceland, 1979; Griffiths et al., 1980; Fantegrossi et al., 2004). Further, they are not associated with a known withdrawal syndrome (O'Brien, 2006). Therefore, there is little risk that exposing human volunteers to hallucinogens will leave participants physically or psychologically dependent on these compounds. In our previous (Griffiths et al., 2006) and ongoing studies with psilocybin, exposing individuals with either no history of hallucinogen use or a history of minimal use (e.g. 1 - 3 times total and not within the last 5 years) in the context of a supervised and controlled research setting has not resulted in reported instances of subsequent illicit hallucinogen abuse.

Perceptual Effects: Another type of potential adverse effect that has been associated with LSD use is a phenomenon of long-lasting perceptual disturbances (i.e. flashbacks). Such effects are extremely rare and associated primarily with LSD use (Strassman 1984; Halpern and Pope 1999; Abraham et al., 2002; Espiard et al., 2005). However, participants will be monitored for persisting visual effects as potential adverse effects of psilocybin.

COVID-19 related risks: COVID-19 is a respiratory virus spread by respiratory droplets, mainly from person-to-person. This can happen between people who are in close contact with one another (less than 6 feet). It is also possible that a person can get COVID-19 by touching a surface or object (such as a doorknob or counter surface) that has the virus on it, then touching their mouth, nose or eyes. For most people, the new coronavirus causes only mild or moderate symptoms, such as fever and cough. For some, especially older adults and people with existing health problems, it can cause more severe illness, including pneumonia. While we are still learning about this virus, the information we have right now suggests that about 3 of 100 people who are infected might die from the virus. Individuals over 60 and with chronic conditions such as cancer, diabetes and lung disease have the highest rates of severe disease from the infection.

b. Steps taken to minimize the risks.

This study proposes a combination of careful screening, preparation, supervision, and follow-up designed to minimize any risks of psilocybin and NRT administration and their aftereffects. Many of these procedures have been used successfully in our previous and ongoing trials with psilocybin. Existing safety data indicate that research using psilocybin in a controlled setting with appropriate screening and preparation will expose participants to minimal risk. Likewise, administration of NRT to carefully screened and prepared individuals under medical supervision will minimize the risk of adverse events. In this study, volunteers will be carefully screened medically and psychologically, and will be carefully monitored throughout the course of treatment. We expect the risk of psychiatric adverse events during psilocybin sessions to be reduced further by the provisions made to help participants feel psychologically at ease and physically safe. The session room will be furnished to be inviting and comfortable. The presence throughout the session of the one or more monitors, with whom the participant will have engaged in a period of trust-building, will provide further reassurance. As a safety measure, after psilocybin has been administered and throughout the period of drug action, physical support or guidance may be provided if we judge participants to be at risk for falls or dangerous behavior.

Study Staff Overseeing Conduct of Psilocybin Sessions: Study staff will have significant human relation skills and have had self-described experience with altered states of consciousness induced by means such as meditation, yoga, or relaxation techniques. Each participant will be introduced to study staff team members before the psilocybin administration in a preparation session to develop rapport. One or more study staff members will be present in the session room with the participant during the psilocybin session. Each study staff member will be specially trained and knowledgeable about the medical and psychological markers of potential adverse reactions to the drug session. Study staff members will also be interviewed by the principal investigator before each drug session. If the study staff, participant, or investigator believes that the session is contraindicated for any reason, the session will be cancelled or postponed.

Medical Emergency Procedures: The physician will be on the Bayview campus and immediately available via pager to come to the research site or for telephone consultation for at least 3 hours or until the peak effects of psilocybin have subsided, whichever is longer. We are confident that this duration of medical coverage is sufficient based on the known time-course of psilocybin, and based on our previous studies of psilocybin (Griffiths et al., 2006, 2011). Acute anxiety, agitation or panic will be handled with reassurance. In the unlikely event that these symptoms do not respond to reassurance, or in the unlikely event that the volunteer experiences psychosis, clinical judgment will determine the most appropriate medical treatment. Oral benzodiazepine will be available for on-site treatment of acute anxiety if needed (e.g., oral diazepam, 5 - 10 mg). An oral anti-psychotic will be available for on-site treatment of psychosis (e.g., oral tablets or orally disintegrating tablets of risperidone, 0.5 - 1 mg). (*Note*: The use of emergency medications for acute anxiety or psychosis will be at the judgment of the treating physician; use of specific medications is not protocolized). In research with psilocybin (our ongoing studies; Strassman 1992; IND 39,258; Strassman personal communication), similar precautions were taken; however, there were no reactions requiring pharmacological or physician intervention. In the unlikely event of a medical or psychiatric emergency that takes place after drug administration and that cannot be safely managed by staff with reassurance or pharmacological intervention, the study team will contact the adjacent Bayview Medical Center Emergency facility. If appropriate, participants will be transferred to the Emergency Department for continued care when symptoms have been sufficiently stabilized.

Cardiovascular Monitoring During Psilocybin Session: During a psilocybin session, if systolic pressure is >170, diastolic >100, or heart rate >110, vital signs will be checked every 5 minutes until systolic pressure is <170, and diastolic <100 and heart rate <110. If blood pressure is >170 to 200 systolic or >100 to 110 diastolic for more than 30 minutes (i.e. after 7 consecutive elevated readings at 5 minute intervals), session monitors will contact the study physician. If there is no evidence that blood pressure is trending upwards, the study physician can authorize changing the frequency of blood pressure assessments to 15 minute intervals. When blood pressure has decreased to (or below) 170 systolic and 100 diastolic, blood pressure assessments will resume at originally scheduled timepoints (e.g. at post-administration minutes 30, 60, etc.).

During a drug session, if blood pressure is >200 systolic or >110 diastolic for three consecutive readings (i.e., 15 minutes), the participant will be treated with sublingual nitroglycerin 0.4mg. If blood pressure readings do not decrease below these thresholds after 5 minutes, the same dose of nitroglycerin will be administered. A third dose of nitroglycerin will be given after another 5 minutes, if readings remain elevated above these levels (maximum dose is 0.4mg x 3). If the need to administer nitroglycerin arises due to sustained elevated blood pressure the physician will administer the drug unless a nurse or nurse practitioner is available at BPRU to administer the drug by order of the physician. If blood pressure remains >200 systolic or >110 diastolic, at the judgment of the treating physician, the participant will be transported to the Emergency Department.

Aversive Reactions to Sublingual Nitroglycerin: The following effects have occurred in clinical trials: headache, hypotension, cutaneous vasodilation with flushing, dizziness and weakness, as well as other signs of cerebral ischemia associated with postural hypotension. Also possible are nausea, vomiting, restlessness, pallor, perspiration and collapse, drug rash and/or exfoliative dermatitis, paresthesia, rhinitis, peripheral edema, asthenia, and abdominal pain.

Continuation of Data Collection: To the extent possible without interfering with appropriate medical response to any adverse effects, we will continue collecting data from volunteers even if adverse effects occur that warrant stopping study participation. Thus, "stopping study participation," means stopping or delaying any study procedures that would interfere with appropriate medical response and care. The study data collection procedures entail minimal risk -- interviews and questions and ratings. They represent the scientific and societal benefit aspects of this study and we believe they should be continued to the extent possible.

Exiting the Drug Session: Participants will remain under observation at least 7 hours and until subjective and objective drug effects have subsided. Participants will be released into the custody of a close friend or family member who has agreed to take charge of the study participant as a condition of leaving the testing facility. In the unlikely circumstance that the friend or family member is unable to pick up the volunteer from the laboratory as scheduled, a staff member will escort the participant home by taxi. Both the study staff and medical personnel will be on call for 24 hours following the drug session in order to assist with any post-session adverse reactions. Although we expect that all drug effects will have subsided and that participants will be ready to leave BPRU 7 to 10 hours after drug administration, participants will be kept overnight and under observation the next day if we judge it to be indicated.

Post-session Headache: Because preliminary observations suggest there may be an association between psilocybin administration and transient headache starting about 7 hours after

administration, on the first meeting after each session, study staff will conduct a formal interview with the volunteer about post-session headache onset, duration and severity.

Persisting visual perceptual effects: To assess for possible persisting visual perceptual effects after psilocybin administration, a questionnaire is included to document the occurrence of various visual phenomena such as changes in color intensity, after-images, and apparent movement of stationary objects. This questionnaire is completed at screening, approximately 1 week after the psilocybin session and at approximately 3, 6, and 12 months after the psilocybin session.

COVID-19 related risks: Participants will complete a pre-visit COVID-19 symptom questionnaire within 24 hours of their visit, and/or be screened with a symptom questionnaire upon arrival at the Behavioral Pharmacology Unit (BPRU). The screening questionnaire will assess for possible COVID-19 symptoms including:

- Elevated body temperature
- Presence of cough
- Loss of smell or taste
- Presence of fatigue or general malaise
- Presence of headache
- Presence of body aches
- Adherence to social distancing guidelines
- Known contact with an individual who tested positive for COVID-19 in the past 14 days
- Engagement in high-risk behaviors (e.g. participation in large gatherings without social distancing or use of PPE)

The screening questionnaire may be subject to change based on new information about COVID-19. Body temperature measurement will also occur upon arrival at the BPRU.

During in-person visits

PPE: Study staff will wear PPE comprised of a surgical mask at a minimum. Face shields will be worn by study staff when confined in a room together at less than 6 feet of distance. Gloves will be worn when any physical contact between staff and participant occurs. Face shields and gloves may be omitted in cases where participants and staff have been vaccinated for COVID-19. Participants will be required to wear a face mask during in-person visits. Surgical masks will be available for participants who do not bring their own. Gloves will also be available. The study team will monitor the data and guidelines on duration of vaccine effectiveness and correlate this to when the vaccine was administered to any given subject when potentially modifying COVID-19 related study safety procedures.

Physical distancing: All or part of some visits may occur using encrypted two-way synchronous audio or video communication in order to minimize face-to-face contact. Staff will maintain at least 6 feet of distance when possible during face-to-face interactions with participants. During interactions that require less than 6 feet of distance (e.g. vital sign measurement), additional PPE such as gloves and face shields may be used.

Cleaning: Equipment will be cleaned between participants. Frequently touched surfaces as specified in the JH SOM Research Visit Guidelines will be cleaned at least every 4 hours when in use (except when it would interfere with 8-hour dosing day procedures).

Measures for psilocybin session day

These sessions often require session facilitator(s) and the participant to be in a session room together for an extended period of time and may require periods of physical contact (e.g. for adjusting blood pressure cuff, therapeutic touch etc.). On dosing day, it is possible that participants may inadvertently remove PPE while under the influence of psilocybin. We will adhere to the following additional safety measures for these sessions:

- COVID-19 testing will be available for participants and facilitators prior to these meetings if there is special concern raised about the possibility of exposure during the final preparation or drug administration day. The availability of testing will be discussed during preparation.

- COVID-19 testing will be performed within 72 hours of each psilocybin session with a negative result, or written proof of such testing with negative result within 72 hours of each psilocybin session must be presented if the participant claims to have been tested. This will be required to move forward with a psilocybin administration session. Participants can alternatively bring proof of full COVID-19 vaccination being completed at least 2 weeks prior to their psilocybin session in lieu of COVID-19 testing. Vaccination status will be verified via official written documentation (e.g., CDC card or healthcare provider records) and copies will be kept on file. Study team members overseeing psilocybin sessions have presently been fully vaccinated.

- Participants will be asked to maintain social distancing procedures and use PPE in public places between the baseline visit and the day after the drug administration session.

- Session facilitators will wear a surgical mask at all times during these two visits. Facilitators will wear a face shield when there is less than 6 feet of space between any two individuals. Face shields may be omitted in cases where participants and staff have been vaccinated for COVID-19.

- Session facilitators will wear gloves when touching the participant (adjusting blood pressure cuff, helping the participant to the bathroom, etc.). Gloves may be omitted in cases where participants and staff have been vaccinated for COVID-19.

- The study team will monitor the data and guidelines on duration of vaccine effectiveness and correlate this to when the vaccine was administered to any given subject when potentially modifying COVID-19 related study safety procedures.

- A washable couch cover will be used and cleaned between participants.

- Headphones will be sanitized between participants, or disposable headphone covers will be used.

Measures for MRI visits

- NIDA IRP will provide point-of-care COVID-19 testing for participants at in-person study visits. Only individuals with negative COVID-19 tests will be allowed to proceed with their scheduled visit. Otherwise they will be advised to quarantine according to CDC guidelines and provide negative COVID-19 test results before resuming in-person study visits as described below.

- NIDA IRP has instituted COVID-19 safety measures consistent with those described above (i.e., physical distancing, PPE and mask use during all visits, pre-visit symptom checks, thorough equipment cleaning between participants), which will be used for participant and personnel safety at in-person MRI visits.

Additional precautions:

- To the extent feasible and agreeable to research subjects, telemedicine/virtual visits may be performed particularly for the delivery of preparatory/integration sessions. This will be accomplished via encrypted synchronous audio/visual communication such as Zoom or Doximity.

- Disinfecting UV light devices may be used in session rooms between participants.

Pre-visit screening:

- If the screening questionnaire completed prior to arrival is clinically concerning for active COVID-19, participants will be provided information on how to obtain SARS-CoV-2 testing. A negative test will be required prior to any in-person visit.

- If clinically concerning symptoms or elevated body temperature are identified on arrival to the BPRU, participants will be provided with information about where to obtain SARS-CoV-2 testing or clinical care as needed. JH SOM guidelines for care of persons under investigation (PUIs) will be followed.

SARS-CoV-2 testing:

- <u>If a staff member tests positive</u>: In the event a study staff member tests positive for active SARS-CoV-2 viral infection, they should immediately discontinue all in-person study activities until cleared to resume work according to current CDC guidelines (accessible at: https://www.cdc.gov/coronavirus/2019-ncov/hcp/return-to-work.html). To the extent the staff's study activities can be performed remotely or without contact with subjects, such work may continue uninterrupted.

- <u>If a session facilitator tests positive</u>: If a SARS-CoV-2 test returns positive for a facilitator, a suitable alternate will be found for the drug administration session. In the event a facilitator tests positive for active SARS-CoV-2 viral infection, they will immediately discontinue all in-person study activities and should follow guidelines above for study staff testing positive for SARS-CoV-2'. Staff will return to work per JH occupational health recommendations.

  - When a facilitator-paired subject has been assigned and a facilitator subsequently tests positive for SARS-CoV-2, a replacement facilitator may be assigned so that the subject may continue progressing through the study.

- Participant tests positive: In the event that a participant tests positive for SARS-CoV-2 prior to the drug dosing day, their study participation will be suspended. A decision about whether the participant can resume study participation or be re-screened will be made by the study team. Reassessment of such participants may begin when the subject has recovered from SARS-CoV-2 infection. Current CDC guidelines for SARS-CoV-2 recovery will be followed to determine SARS-CoV-2 recovery (accessible at: https://www.cdc.gov/coronavirus/2019-ncov/community/strategy-discontinue-isolation.html); however, given the increased risk of transmission between subjects and facilitators during in-person visits, the subject will be required to have negative SARS-CoV-2 test results prior to being re-screened.

Dosing day

- If a participant inadvertently removes his or her PPE during the dosing day, this situation will be treated by staff in a similar manner to when eyeshades or headphones are removed. Staff will assess the participant's mental status and reasons for removing PPE. Staff will address issues as much as possible (e.g. discomfort) and encourage the participant to wear PPE.

The measures outlined above will be adhered to until both JH SOM leadership and the study team agree that risk of SARS-CoV-2 transmission is low enough to warrant resumption of routine in-person practices.

c. Plan for reporting unanticipated problems or study deviations.

The investigators will report adverse events to the IRB according to the Johns Hopkins and federal guidelines.

d. Legal risks such as the risks that would be associated with breach of confidentiality.

While breach of confidentiality could be embarrassing and disturbing, study records involve no documentation of specific illegal acts.

e. Financial risks to the participants.

There will be no financial risks to participants.

9. Benefits
a. Description of the probable benefits for the participant and for society.

The probable benefits from the study for participants is a possible cessation or reduction in cigarette smoking and nicotine dependence. Furthermore, for participants in the psilocybin condition, some mental health and religious professionals have reported that hallucinogens, under specific circumstances of administration, can provide unusual opportunities for deep

reflection. However, no such benefit from the drug session can be promised to volunteers in this study. All volunteers may benefit from the general review of life values and direction that will occur in meetings with the study staff over a period of several months. Society will benefit from gaining a more detailed understanding of the effects of psilocybin on smoking cessation or for other forms of substance dependence. Additionally, the proposed neuroimaging portion of the study will produce novel findings with regards to the intermediate (i.e., next-day) effects of psilocybin, and how these may contribute to longer-lasting behavioral and psychological changes, including smoking cessation.

10. Payment and Remuneration

a. Detail compensation for participants including possible total compensation, proposed bonus, and any proposed reductions or penalties for not completing the protocol.

Volunteers will not receive monetary compensation for the treatment portion of the study (i.e, treatment meetings, follow-ups). However, MRI participating volunteers (n=60) will receive an hourly compensation of $20/hr. for all MRI scanning days, including participation in the preliminary NIDA sub-study protocol (possible total compensation=$480). Funding for this compensation will be provided by NIDA IRP. In order to match potential earnings between all MRI participating volunteers, those who do not undergo a paid 3-month follow-up MRI scan (i.e., who are smoking at that time) will be compensated at a $20/hr. rate for their time during the 3-month follow-up. Funding for this compensation will be provided by the research study budget. No penalties will be assessed for not completing the protocol. Volunteers will receive free parking vouchers for all scheduled study visits.

11. Costs

a. Detail costs of study procedure(s) or drug (s) or substance(s) to participants and identify who will pay for them.

Other than travel to and from the study site, there are no costs to participants. All treatment counseling and pharmacotherapy will be provided at no cost to the participant. Furthermore, participants and family or friends who pick up participants after psilocybin sessions will be provided with free parking vouchers for all in-person meetings.

References:

Abraham, H. D., Aldridge, A. M., & Gogia, P. (1996). The psychopharmacology of hallucinogens. Neuropsychopharmacology, 14, 285-298.

Abraham, H. D., McCann, U. D., & Ricaurte, G. H. (2002). Psychedelic drugs. In K. L. Davis, D. Charney, J. T. Coyle, C. Nemeroff, Neuropsychopharmacology: The Fifth Generation of Progress, (pp. 1545-1556). Philadelphia, PA: Lippincott Williams & Wilkins.

Abuzzahab F S, Anderson B J (1971) A review of LSD treatment in alcoholism. Int Pharmacopsychiat 6: 223-235

Albaugh BJ, Anderson PO. (1974). Peyote in the treatment of alcoholism among American Indians. Am J Psychiatry, 131, 1247-50.

Aubin, H. J., Bobak, A., Britton, J. R., Oncken, C., Billing, C. B., Gong, J., ... & Reeves, K. R. (2008). Varenicline versus transdermal nicotine patch for smoking cessation: results from a randomised open-label trial. *Thorax*, *63*(8), 717-724.

Baer, R. A., Smith, G. T., Hopkins, J., Krietemeyer, J., & Toney, L. (2006). Using self-report assessment methods to explore facets of mindfulness. Assessment, 13, 27-45.

Baker, F., Johnson, M. W., & Bickel, W. K. (2003). Delay discounting in current and never-before cigarette smokers: similarities and differences across commodity, sign, and magnitude. *J Abnorm Psychol*, *112*(3), 382-392.

Beck, AT, Ward, CH, Mendelson, M, Mock J, Erbaugh, J. An inventory for measuring depression. *Arch Gen Psychiatry*. 1961;4(6):561-571.

Bergman RL. (1971). Navajo peyote use: its apparent safety. Am J Psychiatry, 128(6):695-9.

Bickel, W.K. & Johnson, M.W. (2003). Delay discounting: A fundamental behavioral process of drug dependence. In G. Loewenstein, D. Read & R.F. Baumeister (Eds.) Time and Decision. Russell Sage Foundation, New York.

Biener, L., & Abrams, D. B. (1991). The Contemplation Ladder: validation of a measure of readiness to consider smoking cessation. *Health Psychology*, *10*(5), 360.

Blum K, Futterman SL, Pascarosa P. (1977). Peyote, a potential ethnopharmacologic agent for alcoholism and other drug dependencies: possible biochemical rationale. Clin Toxicol., 11, 459-72.

Bush, G, Shin, LM. (2006). The Multi-source Interference Task: an fMRI task that reliably activates the cingulo-frontal-parietal cognitive/attention network. Nature Protocols., 1, 308-313.

Calabrese JD. (1997). Spiritual healing and human development in the Native American church: toward a cultural psychiatry of peyote. Psychoanal Rev., 84, 237-55

Chwelos N, Blewett D B, Smith C M, Hoffer A (1959) Use of d-lysergic acid diethylamide in the treatment of alcoholism. Quart J Stud Alcohol 20: 577-590

Cloninger, C. R., Svrakic, D. M., & Przybeck, T. R. (1993). A psychobiological model of temperament and character. *Archives of General Psychiatry, 50*(12), 975-990. doi:10.1001/archpsyc.1993.01820240059008

Cloninger, C. R. (1999). A new conceptual paradigm from genetics and psychobiology for the science of mental health. *Australian and New Zealand Journal of Psychiatry, 33*(2), 174-186. doi:10.1046/j.1440-1614.1999.00533.x

Cohen, S. (1960). Lysergic Acid Diethylamide: Side effects and complications. Journal of Nervous and Mental Disease, 130, 30-40.

Cole, C., Crum, R.M., Ford, D.E. (2006). Associations between spirituality and substance abuse symptoms in the Baltimore Epidemiologic Catchment Area follow-up. Journal of Addictive Diseases, 25, 125-132.

Cools, R., Clark, L., Owen, A. M., & Robbins, T. W. (2002). Defining the neural mechanisms of probabilistic reversal learning using event-related functional magnetic resonance imaging. *The journal of neuroscience, 22*(11), 4563-4567.

Covey, L.S., Glassman, A.H., Jiang, H., Fried, J., Masmela, J., LoDuca, C., Petkova, E., Rodriguez, K. (2007). A randomized trial of bupropion and/or nicotine gum as maintenance treatment for preventing smoking relapse. Addiction, 102, 1292-1302.

Crumbaugh, J. C., & Maholick, L. T. (1964). An experimental study in existentialism: The psychometric approach to Frankl’s concept of noogenic neurosis. Journal of Clinical Psychology, 20, 200-207.

Derogatis, L. R. (1993). BSI Brief Symptom Inventory. National Computer Systems, Inc.

DiClemente CC, Prochaska JO (1985). Processes and stages of change: Coping and competence in smoking behavior change. In S. Shiffman & T.A. Wills (Eds.), Coping and Substance Abuse (pp. 319-343). New York: Academic Press.

DiClemente CC, Prochaska JO, Fairhurst SK, Velicer WF, Velasquez MM, Rossi JS. (1991). The process of smoking cessation: an analysis of precontemplation, contemplation, and preparation stages of change. J Consult Clin Psychol, 59, 295-304.

DiClemente CC, Prochaska JO, Gibertini, M (1985). Self-efficacy and the stages of self-change of smoking. Cognitive Therapy and Research, 9, 181-200.

Dobkin de Rios M, Grob C S, Baker J R (2002) Hallucinogens and redemption. J Psychoactive Drugs 34: 239-48

Doblin, R. (1991). Pahnke's "Good Friday Experiment": A Long-Term Follow-Up and Methodological Critique. The Journal of Transpersonal Psychology, 23.

Donati, R. J., Schappi, J., Czysz, A. H., Jackson, A., & Rasenick, M. M. (2015). Differential effects of antidepressants escitalopram versus lithium on Gs alpha membrane relocalization. *BMC neuroscience*, *16*(1), 40.

Duke, R. B., & Keeler, M. H. (1968). The effects of psilocybin, dextro-amphetamine and placebo on performance of the trail making test. Journal of Clinical Psychology, 24, 316-317.

Ehrman, RN, Robbins, SJ, Bromwell, MA, Lankford, ME, Moterosso, JR, O'Brien, CP. (2002). Comparing attentional bias to smoking cues in current smokes, former smokes, and non-smokers using a dot-probe task. Drug and Alcohol Dependence., 67, 185-191.

Espiard, M., Lecardeur, L., Abadie, P., Halbecq, I., & Dollfus, S. (2005). Hallucinogen persisting perception disorder after psilocybin consumption: a case study. European Psychiatry, 20, 458-460.

Fantegrossi W E, Woods J H, Winger G (2004) Transient reinforcing effects of phenylisopropylamine and indolealkylamine hallucinogens in rhesus monkeys. Behav Pharmacol 15: 149-157

Fiore, M. C., Kenford, S. L., Jorenby, D. E., Wetter, D. W., Smith, S. S., & Baker, T. B. (1994). Two studies of the clinical effectiveness of the nicotine patch with different counseling treatments. *CHEST Journal*, *105*(2), 524-533.

Foland-Ross LC, Altshuler LL, Bookheimer SY, Lieberman MD, Townsend J, Penfold C, Moody T, Ahlf K, Shen JK, Madsen SK, Rasser PE, Toga AW, Thompson PM. Amygdala reactivity in healthy adults is correlated with prefrontal cortical thickness. J Neurosci. 2010, 30(49):16673-8.

Gable, R. S. (1993). Toward a comparative overview of dependence potential and acute toxicity of psychoactive substances used nonmedically. American Journal of Drug and Alcohol Abuse, 19(3), 263-281.

Gable, R. S. (2004). Comparison of acute lethal toxicity of commonly abused psychoactive substances. Addiction, 99, 686-696.

Garavan, H., Ross, T. J., Li, S. J., & Stein, E. A. (2000). A parametric manipulation of central executive functioning. *Cerebral Cortex*, *10*(6), 585-592.

Garcia-Romeu, A., Griffiths, R. R., & Johnson, M. W. (2015). Psilocybin-occasioned Mystical Experiences in the Treatment of Tobacco Addiction. *Current Drug Abuse Reviews, 7*(3), 157-164. DOI: 10.2174/1874473708666150107121331

Garrity JF. (2000). Jesus, peyote, and the holy people: alcohol abuse and the ethos of power in Navajo healing. Med Anthropol Q, 14, 521-42.

Gilbert, D. G., & Rabinovich, N. E. (1999). International Smoking Image Series, Version 1.2. *Carbondale, Integrative Neuroscience Laboratory, Department of Psychology, Southern Illinois University*.

Gonzales, D., Redtomahawk, D., Pizacani, B., Bjornson, W.G., Spradley, J., Allen, E., Lees, P. (2007). Support for spirituality in smoking cessation: results of pilot survey. Nicotine & Tobacco Research, 9, 299-303.

Gouzoulis-Mayfrank, E., Schreckenberger, M., Sabri, O., et al. (1999a). Neurometabolic effects of psilocybin, 3,4-methylenedioxyethylamphetamine (MDE), and d-methamphetamine in healthy volunteers Neuropsychopharmacology, 20, 565-581.

Gouzoulis-Mayfrank, E., Thelen, B., Habermeyer, E., et al. (1999b). Psychopathological, neuroendocrine and automomic effects of 3,4-methylenedioxyethylamphetamine (MDE), psilocybin and d-methamphetamine in healthy volunteers. Psychopharmacology, 142, 41-50.

Griffiths R R, Bigelow G E, Henningfield J E (1980) Similarities in animal and human drug-taking behavior. Advances in Substance Abuse 1: 1-90

Griffiths, R. R., Johnson, M. W., Richards, W. A., Richards, B. D., McCann, U., & Jesse, R. (2011). Psilocybin occasioned mystical-type experiences: immediate and persisting dose-related effects. *Psychopharmacology*, *218*(4), 649-665.

Griffiths, R. R., Richards, W. A., Johnson, M. W., McCann, U. D., & Jesse, R. (2008). Mystical-type experiences occasioned by psilocybin mediate the attribution of personal meaning and spiritual significance 14 months later. *Journal of psychopharmacology*, *22*(6), 621-632.

Griffiths, R. R., Richards, W. A., McCann, U., & Jesse, R. (2006). Psilocybin can occasion mystical-type experiences having substantial and sustained personal meaning and spiritual significance. *Psychopharmacology*, *187*(3), 268-283.

Grinspoon, L, Bakalar J B (1979) Psychedelic Drugs Reconsidered. Basic Books, New York.

Grob C S (2005) Psilocybin research with advanced-stage cancer patients. Multidisciplinary Association for Psychedelic Studies (MAPS) Bulletin 15: 8

Halpern J H (1996) The use of hallucinogens in the treatment of addiction. Addiction Research 4: 177-189

Halpern J H, Pope H G (1999) Do hallucinogens cause residual neuropsychological toxicity? Drug Alcohol Depend 53: 247-56

Hariri AR, Tessitore A, Mattay VS, Fera F, Weinberger DR. The amygdala response to emotional stimuli: a comparison of faces and scenes. Neuroimage. 2002, 17:317-23.

Hasler, F., Bourquin, D., Brenneisen, R., Bar, T., & Vollenweider, F. X. (1997). Determination of psilocin and 4-hydroxyindole-3-acetic acid in plasma by HPLC-ECD and pharmacokinetic profiles of oral and intravenous psilocybin in man. Pharmaceutica Acta Helvetiae, 72, 175-184.

Hasler, F., Grimberg, U., Benz, M.A., Huber, T., & Vollenweider, F. X.. (2004). Acute psychological and physiological effects of psilocybin in healthy humans: a double-blind, placebo-controlled dose-effect study. Psychopharmacology, 172, 145-56.

Heatherton TF, Kozlowski LT, Frecker RC, Fagerström KO. (1991). The Fagerström Test for Nicotine Dependence: a revision of the Fagerström Tolerance Questionnaire. Br J Addict, 86, 1119-11127.

Hofmann, A. (1983). LSD: My Problem Child. Los Angeles, CA: J. P. Tarcher.

Hofmann, A., Heim, R., Brack. A., & Kobel, H. (1958). Psilocybin, ein psychotroper Wirkstoff aus dem mexikanischen Rauschpilz Psilocybe mexicana Heim. Experientia, 14(3), 107-109.

Hofmann, A., & Troxler, F. (1959). Identifizierung von Psilocin. Experientia, 15(3), 101-102.

Hollister, L. E. (1961). Clinical, biochemical and psychologic effects of psilocybin. Archives Internationales de Pharmacodynamie et de Therapie, 130, 42-52.

Hollister LE, Shelton J, Krieger G. (1969). A controlled comparison of lysergic acid diethylamide (LSD) and dextroamphetamine in alcoholics. Amer. J. Psychiat.. 1969;125:1352-1357.

Hood RW Jr, Ghorbani N, Watson PJ, Ghramaleki AF, Bing MN, Davison HK, Morris RJ, Williamson WP (2001) Dimensions of the mysticism scale: confirming the three-factor structure in the United States and Iran. J Sci Study Relig 40:691–705

Horita, A. (1963). Some biochemical studies on psilocybin and psilocin. Journal of Neuropsychiatry, 4, 270-273.

Hughes, J.R. (2007). Effects of abstinence from tobacco: valid symptoms and time course. Nicotine Tob Res, 9, 315-27.

Hughes, J.R., Keely, J.P., Niaura, R.S., Ossip-Klein, D.J., Richmond, R.L., Swan, G.E. (2003). Measures of abstinence in clinical trials: issues and recommendations. Nicotine & Tobacco Research, 5, 13-25.

Hughes, J.R., Benowitz, N., Hatsukami, D., Mermelstein, Shiffman (2004). Clarification of SRNT workgroup guidelines for measures in clinical trials of smoking cessation therapies. Nicotine & Tobacco Research, 6, 863-864.

Isbell, H. (1959). Comparison of the reactions induced by psilocybin and LSD-25 in man. Psychopharmacologia, 1, 29-38.

Johnson, M. W., Garcia-Romeu, A., Cosimano, M. P., & Griffiths, R. R. (2014). Pilot study of the 5-HT2AR agonist psilocybin in the treatment of tobacco addiction. *Journal of Psychopharmacology, 28*(11): 983-992. DOI: 0269881114548296.

Johnson, M. W., Sewell, R. A., et al. (2012). "Psilocybin dose-dependently causes delayed, transient headache in healthy volunteers." *Drug Alcohol Depend* 123(1-3):132-140.

Kane MJ, Conway AR, Miura TK, Colflesh GJ. Working memory, attention control, and the N-back task: a question of construct validity. J Exp Psychol Learn Mem Cogn. 2007;33:615-22

Kurland A, Savage C, Pahnke W N, Grof S, Olsson J E (1971) LSD in the treatment of alcoholics. Pharmakopsychiatr Neuropsychopharmakol 4: 83-94

Landon, M., & Fischer, R. (1970). On similar linguistic structures in creative performance and psilocybin-induced experience. Confinia Psychiatrica. 13, 115-138.

Lang, P.J.B.M. Cuthbert, B.N. International affective pictures system (IAPS): Technical manual and affective ratings. NIMH Center for the Study of Emotion and Attention (2001).

Lee, B.K., Glass, T.A., McAtee, M.J., Wand, G.S., Bandeen-Roche, K., Bolla, K.I., Schwartz, B.S. (2007) Associations of Salivary Cortisol With Cognitive Function in the Baltimore Memory Study. Arch Gen Psychiatry, 64, 810-8.

Leary, T., Litwin, G. H., & Metzner, R. (1963). Reactions to psilocybin administered in a supportive environment. Journal of Nervous and Mental Disease, 137, 561-573.

Leclerc, G., Lefrançois, R. Dubé, M., Hébert, R., & Gaulin, P. (1998). The self-actualization concept: a content validation. Journal of Social Behavior and Personality, 13, 69-84.

Leclerc, G., Lefrançois, R. Dubé, M., Hébert, R., & Gaulin, P. (1999). Criterion validity of a new measure of self-actualization. Psychological Reports, 85, 1167-1176.

Lefrançois, R., Leclerc, G., Dubé, M., Hébert, R., & Gaulin, P. (1997). The development and validation of a self-report measure of self-actualization. Social Behavior and Personality, 25, 353-366.

Leuner, H. (1962). Die Experimentelle Psychose. Berlin, Germany: Springer Verlag.

Leuner, H. (1981). Halluzinogene: Psychische Grenzzustände in Forschung und Psychotherapie. Bern, Switzerland: Hans Huber Verlag.

Ludwig A, Levine J, Stark L, Lazar R (1969) A clinical study of LSD treatment in alcoholism. Am J Psychiatry 126: 59-69

MacLean, K. A., Leoutsakos, J. M. S., Johnson, M. W., & Griffiths, R. R. (2012). Factor Analysis of the Mystical Experience Questionnaire: A Study of Experiences Occasioned by the Hallucinogen Psilocybin. *Journal for the scientific study of religion*, *51*(4), 721-737.

Malitz, S., Esecover, H., Wilkens, B., & Hoch, H. (1960). Some Observations on psilocybin: a new hallucinogen in volunteer subjects. Comprehensive Psychiatry, 1, 8-17.

Mangini M (1998) Treatment of alcoholism using psychedelic drugs: a review of the program of research. J Psychoactive Drugs 30: 381-418

Marks, D.F. (2005). Overcoming Your Smoking Habit: A Self-help Guide using Cognitive Behavioral Techniques. London: Robinson.

Mayer, F. S., & Frantz, C. M. (2004). The connectedness to nature scale: A measure of individuals' feeling in community with nature. *Journal of Environmental Psychology*, *24*(4), 503-515.

McGlothlin W H, Arnold D O (1971) LSD revisited A ten-year follow-up of medical LSD use. Arch Gen Psychiatry 24: 35-49

McClernon, F. J., Kozink, R. V., & Rose, J. E. (2007). Individual differences in nicotine dependence, withdrawal symptoms, and sex predict transient fMRI-BOLD responses to smoking cues. *Neuropsychopharmacology*, *33*(9), 2148-2157.

McCrae RR. (2009). The five-factor model of personality traits: Consensus and controversy. In P. J. Corr & G. Matthews (Eds.), *The Cambridge Handbook of Personality Psychology* (pp. 148-161). Cambridge: Cambridge University Press.

Menninger, K.A. (1971). Discussion: Navajo peyote use: Its apparent safety, by Robert Bergman. American Journal of Psychiatry, 128, 55.

Metzner, R., Litwin, G., & Weil, G. (1965). The relation of expectation and mood to psilocybin reactions: a questionnaire study. Psychedelic Review, 5, 3-39.
Miller WR. (2004). The phenomenon of quantum change. J Clin Psychol, 60, 453-460.

Miller, W.R., C'de Baka, J. (2001). Quantum Change: When Epiphanies and Sudden Insights Transform Ordinary Lives. New York: Guilford Press

Mills, E. J., Wu, P., Lockhart, I., Wilson, K., & Ebbert, J. O. (2010). Adverse events associated with nicotine replacement therapy (NRT) for smoking cessation. A systematic review and meta-analysis of one hundred and twenty studies involving 177,390 individuals. *Tob Induc Dis*, *8*(8), 1-15.

Mills, E. J., Wu, P., Lockhart, I., Thorlund, K., Puhan, M., & Ebbert, J. O. (2012). Comparisons of high-dose and combination nicotine replacement therapy, varenicline, and bupropion for smoking cessation: A systematic review and multiple treatment meta-analysis. *Annals of medicine*, *44*(6), 588-597.

Moore, D., Aveyard, P., Connock, M., Wang, D., Fry-Smith, A., & Barton, P. (2009). Effectiveness and safety of nicotine replacement therapy assisted reduction to stop smoking: systematic review and meta-analysis. *BMJ: British Medical Journal*, 867-871.

Moreno F A, Wiegand C B, Taitano E K, Delgado P L (2006) Safety, tolerability, and efficacy of psilocybin in 9 patients with obsessive-compulsive disorder. J Clin Psychiatry 67: 1735-1740

Mucha, R. F., Geier, A., & Pauli, P. (1999). Modulation of craving by cues having differential overlap with pharmacological effect: evidence for cue approach in smokers and social drinkers. *Psychopharmacology*, *147*(3), 306-313.

National Institute on Drug Abuse (2001) Hallucinogens and dissociative drugs. National Institute on Drug Abuse, Research Report Series NIH Publication No 01-4209

National Institute on Drug Abuse (2006) LSD, NIDA Infofacts. National Institute on Drug Abuse, Rockville, MD

Nielen, R. J., Van der Heijden, F. M., Tuinier, S., & Verhoeven, W. M. (2004). Khat and mushrooms associated with psychosis. The World Journal of Biological Psychiatry, 5, 49-53.

O'Brien C P (2006) Drug addiction and drug abuse. In: Brunton L L, Lazo J S, Parker K L (eds), Goodman & Gilman's The Pharmacological Basis of Therapeutics 11th ed. McGraw-Hill, New York, pp 607-627

Pahnke, W. (1963). Drugs and mysticism: An analysis of the relationship between psychedelic drugs and the mystical consciousness. Thesis presented to the President and Fellows of Harvard University for the Ph.D. in Religion and Society.

Pahnke, W. N. (1969). Psychedelic drugs and mystical experience. International Psychiatry Clinics, 5(4), 149-162.

Pahnke et al. (1970). The experimental use of psychedelic (LSD) psychotherapy. JAMA, 212, 1856-1863.

Pahnke, W., Salzman, C., Katz, R. 1966. Report on a Pilot Project Investigating the Psychopharmacological Effects of Psilocybin in Normal Volunteers. [unpublished]

Pascarosa P, Futterman S, Halsweig M. (1976). Observations of alcoholics in the peyote ritual: a pilot study. Ann N Y Acad Sci, 273, 518-524.

Passie, T., Seifert, J., Schneider, U., & Emrich, H. M. (2002). The pharmacology of psilocybin. Addiction Biology, 7, 357-364.

Perkins KA, Conklin CA, Levine MD (2008). Cognitive-Behavioral Therapy for Smoking Cessation: A Practical Guidebook to the Most Effective Treatments. New York: Routledge

Petry NM, Bickel WK, Arnett M. (1999). Shortened time horizons and insensitivity to future consequences in heroin addicts. Addiction, 93, 729-38.

Pezawas, L., Meyer-Lindenberg, A., Drabant, E. M., Verchinski, B. a, Munoz, K. E., Kolachana, B. S., Egan, M. F., et al. 5-HTTLPR polymorphism impacts human cingulate-amygdala interactions: a genetic susceptibility mechanism for depression. Nature neuroscience, (2005). 8, 828–34.

Piedmont, R.L. (2004). Spiritual transcendence as a predictor of psychosocial outcome from an outpatient substance abuse program. Psychology of Addictive Behaviors, 18, 213-222.

Piderman, K.M, Schneekloth, T.D., Pankratz, V.S., Maloney, S.D., Altchuler, S.I. (2007). Spirituality in Alcoholics during treatment. The American Journal of Addictions, 16, 232-237.

Piper, M. E., Bullen, C., Krishnan-Sarin, S., Rigotti, N. A., Steinberg, M. L., Streck, J. M., & Joseph, A. M. (2020). Defining and measuring abstinence in clinical trials of smoking cessation interventions: an updated review. Nicotine and Tobacco Research, 22(7), 1098-1106.

Poling A, Bryceland J (1979) Voluntary drug self-administration by nonhumans: a review. J Psychedelic Drugs 11, 185-190.

Posner, K., Brown, G. K., Stanley, B., Brent, D. A., Yershova, K. V., Oquendo, M. A., ... & Mann, J. J. (2011). The Columbia–Suicide Severity Rating Scale: initial validity and internal consistency findings from three multisite studies with adolescents and adults. *American journal of psychiatry*, *168*(12), 1266-1277.

Prochaska JO, Velicer WF, DiClemente CC, Fava J. (1988). Measuring processes of change: applications to the cessation of smoking. J Consult Clin Psychol, 56, 520-8.

Reiss, S., Peterson, R. A., Gursky, D. M., & McNally, R. J. (1986). Anxiety sensitivity, anxiety frequency and the prediction of fearfulness. *Behaviour research and therapy*, *24*(1), 1-8.

Riba J, Rodriguez-Fornells A, Urbano G, Morte A, Antonijoan R, Montero M, Callaway J C, Barbanoj M J (2001) Subjective effects and tolerability of the South American psychoactive beverage Ayahuasca in healthy volunteers. Psychopharmacology 154: 85-95

Richards, W. A. (1975). Counseling, peak experiences and the human encounter with death: An empirical study of the efficacy of DPT-assisted counseling in enhancing the quality of life of persons with terminal cancer and their closest family members. Thesis presented to The Catholic University of America, Washington D.C. Ann Arbor: Xerox University Microfilms, No.75-18, 531.

Richards, W. A., Rhead, J. C., DiLeo, F. B., Yensen, R., & Kurland, A. A. (1977). The peak experience variable in DPT-assisted psychotherapy with cancer patients. Journal of Psychedelic Drugs, 9, 1-10.

Rinkel, M., Atwell, C. R., DiMascio, A., & Brown, J. (1960). Experimental Psychiatry, V. Psilocybine, A New Psychotogenic Drug. New England Journal of Medicine, 262, 295-299.

Roberts, T. B. (1999). Do entheogen-induced mystical experiences boost the immune system? Psychedelics, peak experiences, and wellness. Advances in Mind-Body Medicine, 15, 139-147.

Roberts, T. B. (2006). Psychedelic horizons. Charlottesville, VA: Imprint Academic.

Roy C. (1973). Indian peyotists and alcohol. Am J Psychiatry, 130, 329-30.

Rümmele, W., & Gnirss, F. (1961). Untersuchungen mit Psilocybin, ein psychotropen Substanz aus Psilocybe Mexicana, Schweizerische Archive für Neurologie, Neurochirurgie und Psychiatrie, 87, 365-385.

Rynearson, R. R., Wilson M. R., & Bickford R. G. (1968). Psilocybin-induced changes in psychological function, electroencephalogram, and light-evoked potentials in human subjects. Mayo Clinic Proceedings, 43, 191-204.

SAMHSA (2006a). Table 1.128B – Specific hallucinogen, inhalant, needle, and heroin use in lifetime, by age group: percentages, 2002 and 2003. Office of Applied Studies, National Survey on Drug Use and Health, 2002 and 2003. URL http://oas.samhsa.gov/nhsda/2k3tabs/PDF/Sect1peTabs128to132.pdf

SAMHSA (2006b). Table 4.2 – Top 150 drugs mentioned in ED drug abuse episodes alphabetically, 2002. Office of Applied Studies, Drug Abuse Warning Network, 2002 (03.2003 update). http://dawninfo.samhsa.gov/old_dawn/pubs_94_02/edpubs/2002detailed/default.asp

Savage C, McCabe O L (1973) Residential psychedelic (LSD) therapy for the narcotic addict: a controlled study Arch Gen Psychiatry 28: 808-814

Schwartz, S. H. (1992). Universals in the content and structure of values: Theoretical advances and empirical tests in 20 countries. In. M.P. Zanna (Ed.) Advances in experimental social psychology (Vol. 25, pp. 1-65) New York: Academic Press.

Schwartz, S. H. (1994). Are there universals in the content and structure of values? Journal of Social Issues, 50, 19-45.

Shiffman, S., Dresler, C.M., Hajek, P., Gilburt, S.J., Targett, D.A., Strahs, K.R. (2002). Efficacy of a nicotine lozenge for smoking cessation. Arch Intern Med, 162, 1267-1276.

Shiffman S, West R, Gilbert D; SRNT Work Group on the Assessment of Craving and Withdrawal in Clinical Trials (2004). Recommendation for the assessment of tobacco craving and withdrawal in smoking cessation trials. Nicotine Tob Res, 6, 599-614.

Schultes, R. E. (1940). Teonanacatl: The narcotic mushroom of the Aztecs. American Anthropologist, 42, 429-443.

Sherer, M., Maddux, J. (1982). The self-efficacy scale: construction and validation. Psychological reports, 51, 663-671.

Silagy, C., Lancaster, T., Stead, L., Mant, D., & Fowler, G. (2000). Nicotine replacement therapy for smoking cessation. *The Cochrane Library*.

Smart R G, Storm T, Baker E F W, Solursh L (1966). A controlled study of lysergid in the treatment of alcoholism: The effects on drinking behavior. Quart J Stud Alcohol 27: 469-485

Smith, A., Budavari, S., O'Neil, M. J., Heckelman, P. E., & Kinneary, J. F. (Eds.) (1996). Merck Index: An Encyclopedia of Chemicals, Drugs, and Biologicals (12th ed.). Chapman & Hall.

Spielberger, C. D. (1983). Manual for the State–Trait Anxiety Inventory STAI (Form Y). Palo Alto, CA: Mind Garden

Spilka B, Hood RW, Hunsberger B, Gorsuch R (2005) The psychology of religion: an empirical approach, 3rd edn. Guilford, New York

SRNT Subcommittee on Biological Verification (2002). Biological verification of tobacco use and cessation. Nicotine & Tobacco Research, 4, 149-159.

Stace, W. T. (1960). Mysticism and Philosophy. Philadelphia, PA: Lippincott Williams & Wilkins.

Stapleton, J., West, R., Hajek, P., Wheeler, J., Vangeli, E., Abdi, Z., ... & Sutherland, G. (2013). Randomized trial of nicotine replacement therapy (NRT), bupropion and NRT plus bupropion for smoking cessation: effectiveness in clinical practice. *Addiction*, *108*(12), 2193-2201.

Stead, L. F., Perera, R., Bullen, C., Mant, D., Hartmann-Boyce, J., Cahill, K., & Lancaster, T. (2012). Nicotine replacement therapy for smoking cessation. *Cochrane Database Syst Rev*, *11*(11).

Strassman, R. (1984). Adverse reactions to psychedelic drugs: A review of the literature. The Journal of Nervous and Mental Disease, 172(10), 577-595.

Strassman, R. (1992). Application for Investigational New Drug Permit for Psilocybin. Food and Drug Administration, IND # 39,258.

Strassman, R. (1998). Letter dated 8 March 1998.

Strassman R J (2001) DMT: The Spirit Molecule. Park Street Press, Rochester, VT

Strassman R J, Qualls C R, Uhlenhuth E H, Kellner R (1994) Dose-response study of N,N-dimethyltryptamine in humans II: subjective effects and preliminary results of a new rating scale. Arch Gen Psychiatry 51: 98-108

Studerus, E., Kometer, M., Hasler, F., & Vollenweider, F. X. (2011). Acute, subacute and long-term subjective effects of psilocybin in healthy humans: a pooled analysis of experimental studies. *Journal of Psychopharmacology, 25,* 1434.

Studerus, E., Gamma, A., Kometer, M., & Vollenweider, F. X. (2012). Prediction of psilocybin response in healthy volunteers. *PloS one, 7*(2), e30800.

Sutherland, M. T., McHugh, M. J., Pariyadath, V., & Stein, E. A. (2012). Resting state functional connectivity in addiction: Lessons learned and a road ahead. *Neuroimage*, *62*(4), 2281-2295.

Swan, G.E., Jack, L.M., Ward, M.M. (1997). Subgroups of smokers with different success rates after use of transdermal nicotine. Addiction, 92, 207-217.

Sykes, C.M., Marks, D.F. (2001). Effectiveness of a cognitive behaviour therapy self-help programme for smokers in London, UK. Health Promotion International, 16, 255-260.

Tellegen, A. (1982). Brief manual for the multidimensional personality questionnaire. Minneapolis, University of Minnesota.

Tiffany ST, Drobes DJ. (1991). The development and initial validation of a questionnaire on smoking urges. Br J Addict, 86, 1467-76.

Turek, I. S., Soskin, R. A., & Kurland, A. A. (1974). Methylenedioxyamphetamine (MDA) Subjective Effects. Journal of Psychedelic Drugs, 6, 7-14.

Usdin, E., & Efron, D. (1972). Psychotropic drugs and related compounds (2nd ed.). Bethesda, MD: National Institutes of Mental Health.

Van Dixhoorn J, Duivenvoorden HJ. (1985). Efficacy of Nijmegen Questionnaire in Recognition of the Hyperventilation Syndrome. J Psychosom Res, 29, 199-206.

Varadhan, R., Walston, J., Cappola, A.R., Carlson, M.C., Wand, G.S., Fried, L.P. (2008). Higher levels of blunted diurnal variation of cortisol in frail older women. J Gerontol A Biol Sci Med Sci., 63, 190-195.

Velicer WF, DiClemente CC, Prochaska JO, Brandenburg N. (1985). Decisional balance measure for assessing and predicting smoking status. J Pers Soc Psychol, 48, 1279-1289.

Vollenweider, F. X., Leenders, K. L., Scharfetter, C., et al. (1997). Positron emission tomography and fluorodeoxyglucose studies of metabolic hyperfrontality and psychopathology in the psilocybin model of psychosis. Neuropsychopharmacology, 16, 357-372.

Vollenweider, F. X., Vollenweider-Scherpenhuyzen, M. F. I., Babler, A., Vogel, H., & Hell, D. (1998). Psilocybin induces schizophrenia-like psychosis in humans via a serotonin-2 agonist action. NeuroReport, 9, 3897-3902.

Vollenweider, F. X., Vontobel, P., Hell, D., & Leenders, K. L. (1999). 5-HT modulation of dopamine release in basal ganglia in psilocybin-induced psychosis in man – a PET study with [^{11}C]raclopride. Neuropsychopharmacology, 20, 424-433.

Wallace, M. (1956). Future time perspective in schizophrenia. J Abnorm Psychol, 52, 240-5

Ware J, Scherbourne C. (1992). The MOS 36-item short-form health survey (SF-36): Conceptual framework and item selection. Med Care. 30, 473–83.

Watson, D., & Clark, L. A. (1994). The PANAS-X manual for the positive and negative affect schedule – expanded form. The University of Iowa, Iowa City, Iowa.

Watson, D., & Clark, L. A. (1997). Measurement and mismeasurement of mood: Recurrent and emergent issues. Journal of Personality Assessment, 68, 267-296.

Welsch SK, Smith SS, Wetter DW, Jorenby DE, Fiore MC, Baker TB. (1999). Development and validation of the Wisconsin Smoking Withdrawal Scale. Exp Clin Psychopharmacol, 7, 354-61.

Zemore, S.E. (2007). A role for spiritual change in the benefits of 12-step involvement. Alcoholisms: Clinical and Experimental Research, 31, 76S-79S.

Zimbardo, P.G., Boyd, J.N. (1999). Putting time in perspective: A valid, reliable individual-differences metric. Journal of Personality and Social Psychology, 77, 1271-1288.
