# Supplementary material for: Psilocybin or Nicotine Patch for Smoking Cessation: A Pilot Randomized Clinical Trial
Source: JAMA Netw Open. 2026 Mar 10;9(3):e260972. doi: 10.1001/jamanetworkopen.2026.0972 (PMC12976795; doi:10.1001/jamanetworkopen.2026.0972)
Supplement: Supplement 3. — Data Sharing Statement [file jamanetwopen-e260972-s003.pdf]

Data Sharing Statement

Johnson. Psilocybin or Nicotine Patch for Smoking Cessation. *JAMA Netw Open*. Published March 10, 2026. doi:10.1001/jamanetworkopen.2026.0972

Data
Additional Information: https://clinicaltrials.gov/study/NCT01943994
Data available: Yes
Data types: Deidentified participant data
How to access data: https://data.mendeley.com/datasets/psdmryynyw/1
When available: With publication

Supporting Documents
Document types: None

Additional Information
Who can access the data: Data will be made available upon reasonable request to the PI
Types of analyses: for specified analyses
Mechanisms of data availability: without investigator support
